# An Orthogonal ‘Clickable’ Xyloside Scaffold in ^4^*C*_1_ and ^1^*C*_4_ Conformation

**DOI:** 10.3390/molecules31142432

**Published:** 2026-07-11

**Authors:** Lorenz Pietsch, Sönke Sdunnus, Thisbe K. Lindhorst

**Affiliations:** Otto Diels Institute of Organic Chemistry, Christiana Albertina University of Kiel, 24118 Kiel, Germany; lpietsch@oc.uni-kiel.de (L.P.); ssdunnus@oc.uni-kiel.de (S.S.)

**Keywords:** carbohydrate chemistry, click chemistry, heterobivalent glycoclusters, inverse chair conformations, carbohydrate recognition, bacterial adhesion

## Abstract

Glycoclusters are important molecular tools for studying carbohydrate–protein interactions. It is advantageous to derive glycoclusters from carbohydrate-based scaffolds. Modulating the chair conformation of the scaffolding monosaccharide between ^4^*C*_1_ and ^1^*C*_4_ enables the variation in the spatial organisation of the resulting glycoclusters, allowing the consequences for carbohydrate recognition in biological systems to be studied. Recently, we introduced a xylose derivative capable of adopting two complementary chair conformations, which was conjugated to glycoligands via Suzuki–Miyaura cross-coupling to afford homobivalent glycoclusters. Here, we expand this approach by further exploring the potential of xylose as a conformationally ‘switchable’ carbohydrate scaffold. Two additional features were introduced, (i) alkyne groups to facilitate conjugation reactions through click chemistry, and (ii) orthogonal protection to enable the synthesis of heterobivalent glycoclusters in addition to homobivalent analogues. This strategy enabled the synthesis of a focused library of homo- and heterobilvalent glycoclusters, which were subjected to preliminary biological evaluation. Notably, inversion of the chair conformation of the xyloside scaffold exerted a significant effect on the potency of the respective compounds as inhibitors of mannose-specific bacterial adhesion.

## 1. Introduction

It is well established that carbohydrate–protein interactions are fundamental to cellular processes. They govern cell–cell recognition under both physiological and pathophysiological conditions and mediate the adhesion of microbes to the glycosylated surface of host cells, known as ‘glycocalyx’ [1,2].

What remains poorly understood, however, are the molecular details of carbohydrate–protein interactions within the complex supramolecular environment of the cell surface. This includes the consequences of multivalency and heteromultivalency as well as the specific effects arising from the relative spatial arrangement of carbohydrate ligands. A comprehensive understanding of the specificity and regulation of these processes has yet to be achieved.

Functional glycomimetics represent an important tool for investigating the molecular basis of carbohydrate–protein interactions, as they enable the systematic investigation of defined aspects of molecular recognition in a bottom-up approach [3,4]. Over the past years, we and others have designed and studied numerous such functional glycomimetics [5,6,7,8,9,10]. More recently, we have turned our attention to a new class of functional glycomimetics, so-called conformational carbohydrate switches [11]. These are glycoconjugates in which inversion of the chair conformation of a central sugar scaffold modulates the three-dimensional architecture of a glycocluster [12].

**Figure 1:**
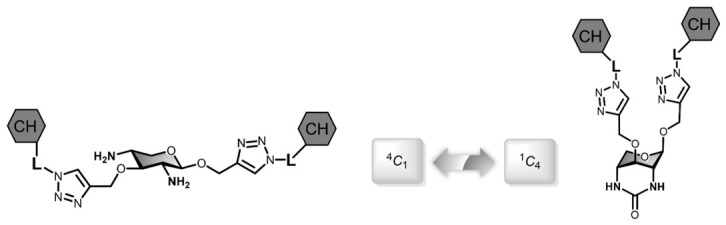
Design concept of a ‘clickable’ xyloside scaffold both in ^4^*C*_1_ and ^1^*C*_4_ conformation. Carbohydrate ligands (CH) can be conjugated to the xyloside via triazole linkages employing various linker units (L), as illustrated. The two amino functionalities at the C-2 and C-4 positions are essential for inducing inversion of the chair conformation from ^4^*C*_1_ to ^1^*C*_4_. Conformational variation of the xyloside scaffold enables the synthesis of glycoclusters with different spatial organisation.

Conformational variation of the sugar ring is biologically relevant, as nature itself translates specific ring conformational changes into biological function. A prominent example is the anticoagulant polysaccharide heparin, where the skew-boat conformation of an iduronic acid residue governs its antithrombotic activity [13]. Against this background, we recently derivatised xylose such that both its ^4^*C*_1_ and ^1^*C*_4_ conformations could be employed as scaffolds for the synthesis of homobivalent glycoclusters [12]. We took inspiration from the pioneering work of Yuasa and Hashimoto, who first explored the xylose scaffold as a conformational switch [14,15,16,17,18,19]. Our goal has since been to further explore the potential of xylose as a conformationally ‘switchable’ carbohydrate scaffold. Whereas Suzuki–Miyaura cross-coupling was previously employed for the conjugation of glycoligands to the xylose scaffold [12], in the present work we introduced alkyne functionalities to render the xyloside scaffold accessible to copper-catalysed azide-alkyne cycloaddition (CuAAC), since click chemistry has proven highly effective for the synthesis of numerous glycoconjugates [20,21,22,23]. Furthermore, to facilitate the construction of heterobivalent glycoclusters based on such a ‘clickable xyloside’, it was equipped with two orthogonally addressable alkyne groups, one of which was protected with a triisopropylsilyl group (TIPS) [24]. In addition, two masked amino functionalities at ring positions 2 and 4 were incorporated according to Yuasa and Hashimoto to enable the inversion of the chair conformation from ^4^*C*_1_ and ^1^*C*_4_ via intramolecular urea formation (Figure 1).

In this work, we describe the synthesis of such clickable xylosides both in ^4^*C*_1_ and ^1^*C*_4_ conformation and their conjugation to carbohydrate ligands via CuAAC reactions. A small library of glycoclusters bearing one or two α-d-mannoside (Man) ligands, as well as a combination of Man and β-d-glucopyranosyl (Glc) residues, was synthesised, demonstrating the modularity and high efficiency of this approach.

For preliminary biological evaluation, these glycoclusters were investigated as inhibitors of lectin-mediated bacterial adhesion. Type 1 fimbriae are adhesive organelles on the surface of bacterial cells, terminated by the lectin domain FimH, which selectively recognises α-d-mannopyranosyl ligands. Type 1 fimbriae-mediated adhesion of *E. coli* is a well-established biological system suitable for investigating the mechanistic details of carbohydrate recognition.

## 2. Results

### 2.1. Synthesis

**Scheme 1:**
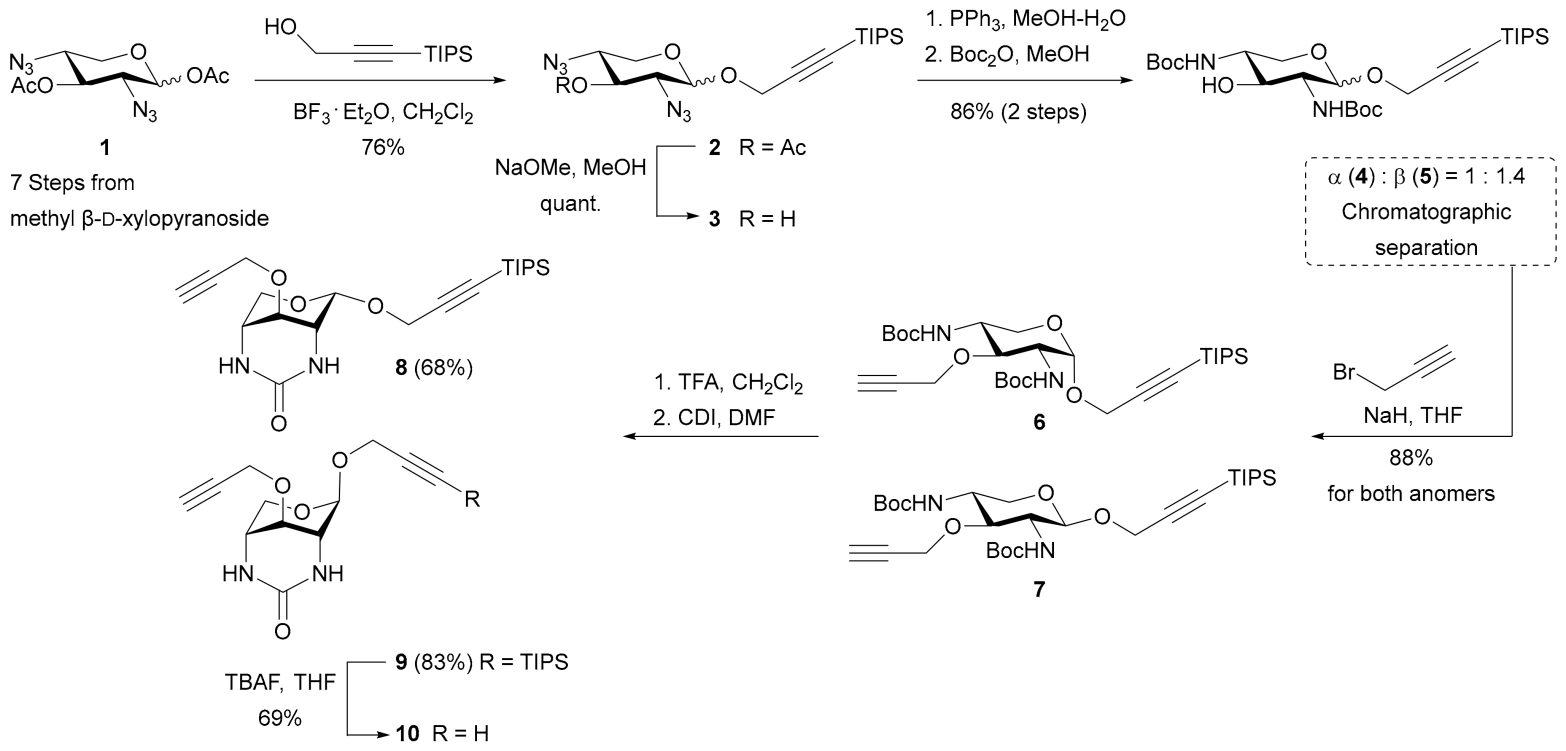
Synthesis of four tetrafunctional ‘clickable’ xylosides, two anomeric ^4^*C*_1_ conformers (**6** and **7**) and two anomeric inverted chair (^1^*C*_4_) conformers (**8** and **9**). CDI: 1,1′-Carbonyldiimidazole.

The synthetic route towards the targeted tetrafunctional ‘clickable’ xylose switch started from the literature-known 2,4-diazido-xylopyranose derivative **1** (Scheme 1), which was synthesised in seven steps from methyl xyloside according to improved procedures published by Yuasa and Hashimoto (see Supplementary Materials Sections S1.1–S1.5) [14]. The subsequent steps aimed at the introduction of two alkyne groups for click chemistry, which can be addressed orthogonally. To this end, first a simple Lewis acid-catalysed glycosidation of **1** with 3-(triisopropylsilyl)propargyl alcohol was carried out in which the anomeric *O*-acetyl group serves as the leaving group. The desired xyloside **2** was obtained in 76% yield and an α:β ratio of 2:3 after purification. Next, the 3-*O*-acetyl group of **2** was quantitatively removed under Zemplén conditions to avoid *O*→*N*-acetyl group migration in the following step. The resulting glycoside **3** was submitted to a two-step reaction sequence, in which both azido groups were reduced to the respective amino functionalities in a Staudinger reduction and subsequently, Boc-protected in the same pot to furnish the anomeric xyloside mixture **4**/**5**. At this stage of the synthesis, the anomers could be separated by column chromatography yielding 36% of the α-d-xyloside **4** and 50% of the β-d-xyloside **5**. In the next step, the 3-OH group was alkylated using propargyl bromide under Williamson etherification conditions to furnish **6** and **7**, both in 88% yield.

The ^1^H NMR spectra of xylosides **4** to **7** exhibit broadened signals attributable to the presence of Boc rotamers within these sugar derivatives. Comparable observations were reported and discussed previously for analogous systems [25,26]. At this stage, it is critical to establish the conformation of the xyloside ring. Variable-temperature NMR measurements performed at different temperatures (see Supplementary Materials Section S2) demonstrated that the observed line broadening originated from carbamate rotamers rather than from conformational dynamics of the xylose ring, which have previously been described for related xylose derivatives [11]. When conducted at low temperature, the NMR spectra reveal coupling constants consistent with a ^4^*C*_1_ chair conformation.

In order to obtain the xylose scaffold in its ^1^*C*_4_ conformation, the amino groups at positions 2 and 4 of the pyranose ring were locked in the form of a cyclic urea derivative. To this end, the Boc protection was removed with trifluoroacetic acid in dichloromethane and the cyclic urea formed with carbonyldiimidazole (CDI) in a one-pot procedure to afford xylosides **8** and **9** in yields of 68% and 83%, respectively. Furthermore, deprotection of the TIPS group in **9** afforded **10**.

**Scheme 2:**
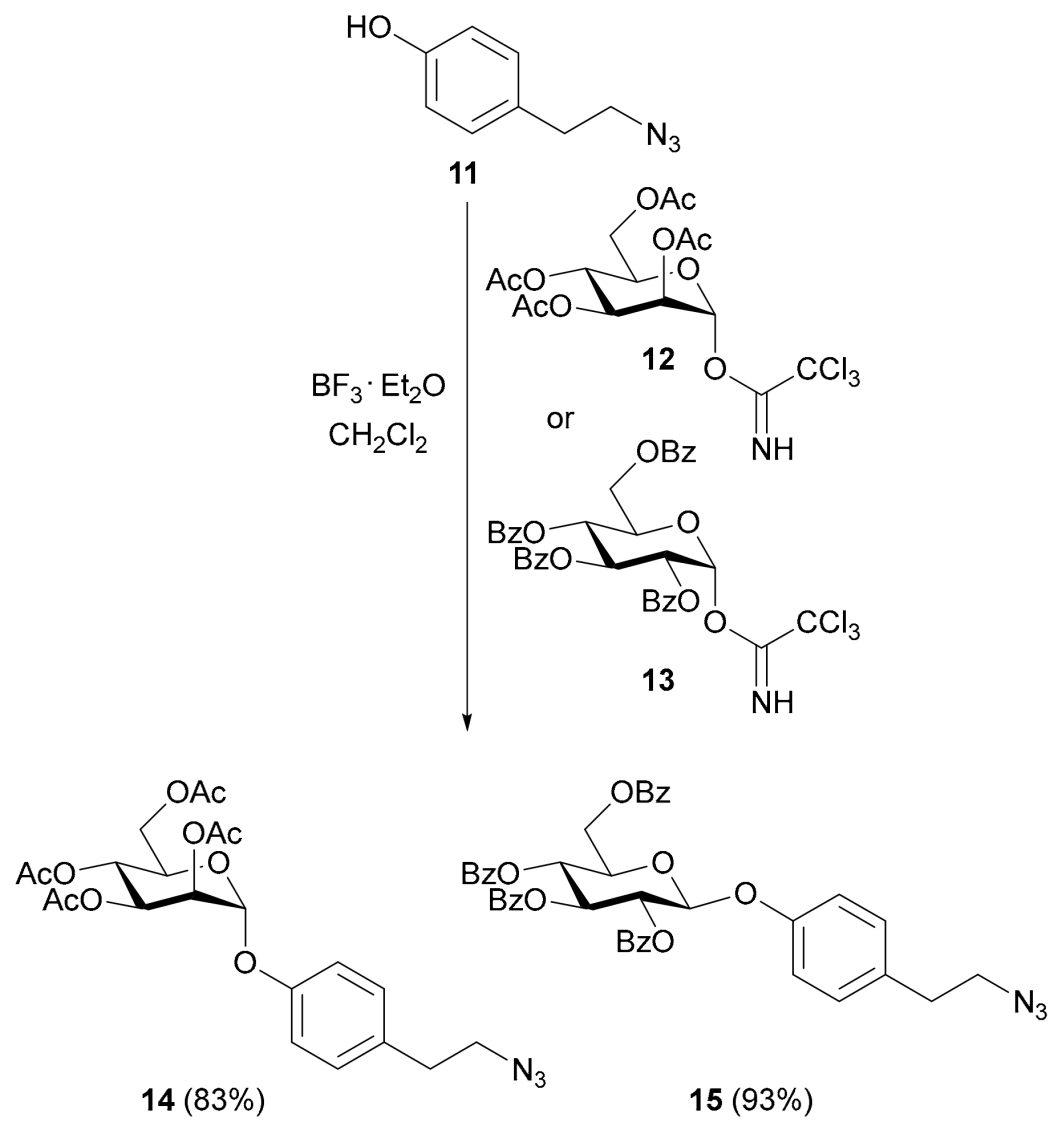
Synthesis of 4-(2-azidoethyl)phenyl mannoside **14** and glucoside **15**.

The alkyne-functionalised xylosides are amenable to further ligation by click chemistry. To explore the synthesis of glycoclusters via CuAAC, we aimed to prepare Man/Man homobivalent glycoclusters and Man/Glc heterobivalent glycoclusters in the context of previous work on the design of inhibitors of type 1 fimbriae-mediated bacterial adhesion [3,12]. As click reaction partners, the literature-known 4-(2-azidoethyl)phenyl mannoside **14** [27] and its *gluco*-configured analogue **15** were selected. These compounds were obtained in a Lewis acid-catalysed glycosylation of 4-(2-azidoethyl) phenol **11** [28,29] with the well-known *O*-glycosyl trichloroacetimidate donors **12** [30] and **13** [31] (Scheme 2). The aglycone moiety was deliberately chosen to be an aryl moiety, as an aryl group at the anomeric centre is known to enhance the affinity of mannosides for the lectin FimH, which mediates adhesion of type 1-fimbriated bacteria [32,33].

Glycoclusters were synthesised employing both the ^4^*C*_1_ conformer **7** and its inverted conformer **10** (cf. Scheme 1) via CuAAC ligation. In contrast, the anomers **6** and **8** (Scheme 1) were not investigated further, as our aim was to maximise the structural difference between the ^4^*C*_1_ and ^1^*C*_4_ conformations of the xylose scaffold. This difference is most pronounced in compounds **7** and **10**, which display the 1- and 3-propargyloxy substituents in a diequatorial arrangement (**7**) and a diaxial arrangement (**10**), respectively.

**Scheme 3:**
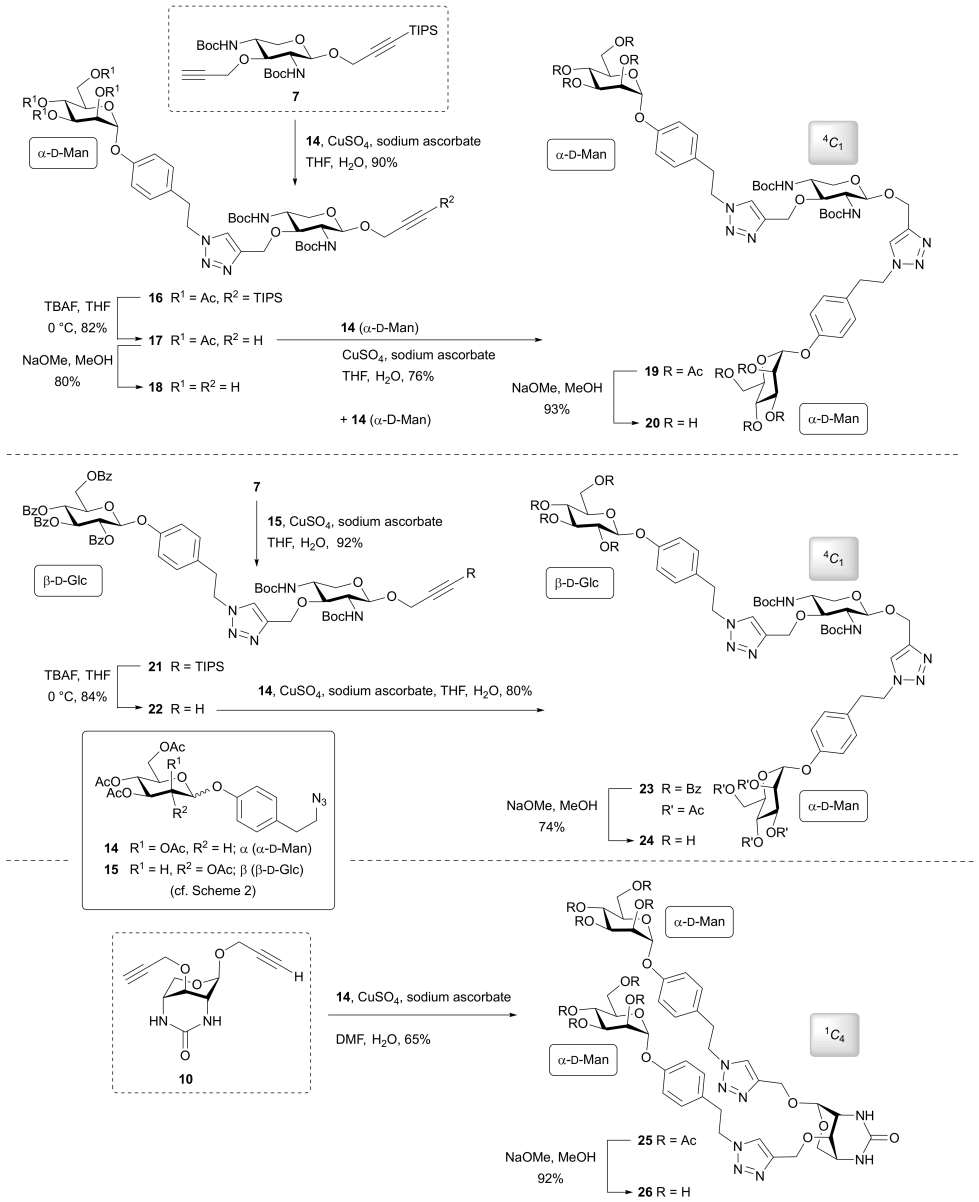
Synthesis of the glycoconjugates **18**, **20**, **24**, and **26** employing click chemistry.

The click reaction of **7** with the mannoside **14** afforded **16** (Scheme 3). After removal of the TIPS protecting group, the resulting alkyne derivative **17** was amenable to a second click reaction. In this case, **17** was again reacted with **14** to give **19**. Final Zemplén deprotection of **17** and **19** provided the monovalent glycoconjugate **18** (bearing one aryl mannoside ligand) and the bivalent Man/Man glycoconjugate **20**, respectively. The analogous procedure afforded **21** from **7** and **15**, and subsequent removal of the TIPS group furnished **22**, which was then subjected to a second CuAAC reaction with **14** to give the heterobivalent Man/Glc glycocluster **23**. Finally, Zemplén deprotection provided the target compound **24**. Ultimately, the di-*O*-propargylated xyloside **10**, present in a ^1^*C*_4_ conformation, was reacted with **14** to afford the corresponding Man/Man glycocluster analogous to **20**, but featuring an inverted chair conformation of the central xyloside scaffold. Owing to the higher solubility of **10** in degassed DMF than in THF, DMF was used as the organic solvent in this reaction.

All of these reactions were high-yielding, demonstrating that a clickable xyloside scaffold provides a straightforward and versatile entry into a broad variety of glycoclusters, in which the orientation of the ligated glycoligands can be modified via the conformation of the central xyloside chair.

To evaluate if these synthetic glycoclusters are amenable to biological testing, a preliminary investigation as inhibitors to type 1 fimbriae-mediated adhesion of *E. coli* bacteria was performed. All four deprotected glycoconjugates, **18**, **20**, **24**, and **26**, have in common that they contain at least one mannose unit as ligand for the type 1 fimbrial lectin FimH. The monovalent Man ligand **18** was compared with the divalent analogue **20**; the divalent Man/Man ligand **20**, built on a xyloside in the ^4^*C*_1_ conformation, was compared with its analogue **26**, constructed on a ^1^*C*_4_ xyloside scaffold; and finally, the effect of an additional β-d-glucosyl residue, as in the Glc/Man glycocluster **24**, was assessed by comparing **24** with **18** and **20**.

### 2.2. Preliminary Biological Evaluation

The glycoclusters **18**, **20**, **24**, and **26** (cf. Scheme 3) were evaluated as inhibitors of the mannose-specific adhesion of live type 1-fimbriated *E. coli* in an adhesion–inhibition assay, in which the test compounds compete with a mannan-coated surface for binding to FimH [34] (see Supplementary Materials Section S3). A GFP (green fluorescent protein)-expressing strain PKL1162 [34] was used, allowing correlation of inhibitory potency with the fluorescence signal arising from bacteria remaining on the mannan surface after inhibition and washing steps. Hence, stronger inhibitors correspond to lower fluorescence intensity.

Serial dilutions of the test compounds were employed on microtitre plates resulting in sigmoidal inhibition curves, from which IC_50_ values were deduced. To ensure inter-assay comparability, a reference compound must be included in every individual assay. Here, methyl α-d-mannopyranoside (MeMan) was used following previous work [12]. MeMan is known as a rather weak inhibitor of FimH-mediated bacterial adhesion. The inhibitory potencies of α-d-mannopyranosides with aromatic aglycon, such as phenyl α-d-mannopyranoside, are about two orders of magnitude higher than that of MeMan [33,35], owing to the aromatic aglycone moiety, which can engage in favourable interactions with the so-called tyrosine gate at the entrance of the FimH carbohydrate recognition domain (CRD) [32,33].

The IC_50_ values and relative inhibitory potencies (RIP), calculated with MeMan defined as unity, are summarised in Table 1.

The inhibitory potencies of all tested glycoclusters, not surprisingly, lie within the range observed for mannoside FimH antagonists carrying an aromatic aglycone moiety [35]. The monovalent Man ligand **18** shows the highest inhibitory potency in the tested series together with the divalent Man/Man glycocluster **26** (RIP ~200). Notably, the other tested divalent Man/Man glycocluster **20** exhibits a significantly weaker inhibitory potency (RIP ~120), nearly a twofold reduction compared to **26**. This difference might be attributed to the mode of ligand presentation since in both **20** and **26** two identical mannoside ligands are conjugated at the 1 and 3 positions of a xyloside scaffold, which, however, adopts different conformations, namely ^4^*C*_1_ and ^1^*C*_4_, respectively. It might be argued that the incorporation of a second Man antenna in **20** introduces steric hindrance, whereas this effect is not pronounced in **26**. The basis of this difference might be found in the conformation of the central xyloside, ^4^*C*_1_ or ^1^*C*_4_, which governs the spatial organisation of the two Man antennae. From the results obtained with the heterobivalent Man/Glc glycocluster **24**, it can be seen that the introduction of a Glc antenna does not improve FimH affinity; rather, it exerts a diminishing effect.

It is intriguing to observe the difference between **20** and **26**. In spite of the glycoligands being conjugated to the xylose scaffold through rather long and flexible spacers, the conformation of the central sugar, ^4^*C*_1_ versus ^1^*C*_4_, still causes a significantly divergent inhibitory potencies. This finding confirms that conformational glycoswitches are powerful glycomimetics, enabling effective modulation of glycoligand presentation simply by flipping the carbohydrate chair conformation. However, the preliminary data obtained in the present series of glycoclusters must be further evaluated in future studies, in which more rigid spacers should also be employed.

We note that click chemistry facilitates the straightforward and modular synthesis of gycoswitches bearing variable carbohydrate ligands, such as the glycoconjugates **18**, **20**, **24**, and **26** described here, and is well suited for the rapid access to further structural variations.

## 3. Materials and Methods

### 3.1. General Information Regarding Synthesis and Spectroscopy

Purchased chemicals were used without further purification. Oxygen-sensitive reactions (such as the conducted click reactions) were performed with degassed solvents and carried out under a nitrogen atmosphere. Dry solvents were purchased or from a Grubbs solvent system (PureSolv MD 5 from Inert, Amesbury, MA, USA). As an exception, pyridine was stored and dried over a mixture of sodium and potassium hydroxide. Employed molecular sieves was freshly activated before usage by heating the Schlenk flask containing molecular sieves under vacuum between 200 and 600 °C for at least 15 min with a Steinel (Herzebrock-Clarholz, Germany) HL 1920 E hot air gun. Reactions that were not carried out in a pressure tube were performed at the pressure prevailing in the laboratory (~1 bar). The room temperature in the laboratory varied between 14 and 35 °C but most of the time, reactions were performed around 20 °C. Flash chromatography on silica gel columns was performed with silica gel 60 M with a particle size between 0.040 and 0.063 mm. Automated flash chromatography was carried out on two different machines: puriFlash450 and puriFlash5.020 which has an ELSD (Evaporative Light Scattering Detector, Interchim^®^, Montluçon, France) helping to identify non-aromatic compounds. In case of automated purification, prepacked silica gel columns (different sizes 12 g, 25 g, 40 g and 80 g (particle size 30 µm) were used from Interchim^®^ (Montluçon, France). For reversed phase flash chromatography either 12 g or 25 g columns with C18-AQ, particle size 15 µm were used from Interchim^®^. Full characterisation of the synthesised compounds by NMR spectroscopy was carried out with the help of 2D spectral data (COSY, HSQC, HMBC) measured on an AVANCE 600 spectrometer (Bruker, Billerica, MA, USA). Thin layer chromatography (TLC) was carried out to monitor reaction processes and for characterisation of the respective compounds. The following plates were used: ALUGRAM^®^ Xtra SIL G UV 254 from Macherey Nagel (Düren, Germany). First, the TLC plate was analysed with a UV-lamp from CAMAG^®^ (Muttenz, Switzerland) (λ_1_ = 254 nm, λ_1_ = 365 nm) followed by visualisation using staining solutions like 10% sulfuric acid in ethanol or potassium permanganate stain which was prepared freshly every month by mixing 1.5 g KMnO_4_, 10 g K_2_CO_3_, and 1.3 mL of an aqueous 10% NaOH solution in 200 mL water. Infrared spectra were recorded on either a FT-IR spectrometer from PerkinElmer (Shelton, CT, USA) with a Golden-Gate-ATR unit or on a Spektrum Two FT-IR spectrometer from PerkinElmer (Shelton, CT, USA) with a UATR unit. An MCP 5100 polarimeter from Anton Paar (Graz, Austria) was used to measure optical rotation. The sample concentration *c* in the respective solvent is given in [$c=\frac{g}{{100\;\mathrm{cm}}^{3}}$] with $[\alpha {]}_{D}^{20}$ is given in [$\frac{mL}{g\times dm}$]. Optical rotations were measured at 589 nm. The laboratory freeze-dryer from CHRIST (Osterode am Harz, Germany) is an ALPHA 2-4 LD plus operating at −80 °C to −90 °C with a pressure of ~ 0.003 ± 0.003 mbar. ESI mass spectra were recorded on either a Thermo Scientific Q Exative plus (mass spectrometer) (Waltham, MA, USA) or a SCIEX (Marlborough, MA, USA) X500R QTOF with an ESI Turbo V Twinsprayer source. In some cases, an HPLC separation (Nexara from Shimadzu, Kyoto, Japan) was performed prior to the measurement. The collection of the mass spectral data and NMR spectral data were recorded by the employees of the respective department. NMR spectra were recorded at the Otto Diels Institute of Organic Chemistry and mass spectrometry was carried out at the Otto Diels Institute of Organic Chemistry or at the Department of Pharmaceutical Biology at Kiel University.

### 3.2. Synthesis

*Glycoconjugate* **18**. The protected glycoconjugate **17** (37.4 mg, 40.7 µmol) was suspended in dry methanol (3.0 mL) under a nitrogen atmosphere and sodium methoxide (5.4 m in methanol, 12.5 µL, 67.5 µmol) was added dropwise. The reaction mixture was stirred at room temperature for 2 h. It was neutralised by addition of Amberlite^®^ (Thermo Fisher Scientific, Fair Lawn, NJ, USA)--IRC 120 H^+^ resin. The resin was separated and the crude product was purified on reversed phase silica gel by automated flash chromatography (H_2_O:MeCN, 100:0 → 0:100) to obtain the title compound **18** after lyophilisation (24.5 mg, 32.7 µmol, 80%); $[\alpha {]}_{D}^{20}$ = +27.027 (c = 0.10, MeOH); ^1^H NMR (600 MHz, CD_3_OD, 298 K): δ = 7.70 (s, 1H, C=CH_Triazole_), 7.08–7.00 (m, 4H, CH*_m_*_-Phenol_, CH*_o_*_-Phenol_), 5.43 (d, ^3^*J*_1,2_ = 1.8 Hz, 1H, H-1_Man_), 4.79–4.74 (m, 2H, N-C-CH_2at C-3_), 4.61–4.54 (m, 1H, H-1_Xylose_), 4.57 (t, ^3^*J*_CH2,CH2_ = 7.3 Hz, 2H, CH_2_-N), 4.34–4.25 (m, 2H, N-C-CH_2at C-1_), 3.98 (dd, ^3^*J*_2,3_ = 3,4 Hz, ^3^*J*_1,2_ = 1.8 Hz, 1H, H-2_Man_), 3.90 (br dd, ^2^*J*_5ax,5eq_ = 11.2 Hz, ^3^*J*_4,5eq_ = 3.3 Hz, 1H, H-5eq_Xylose_), 3.88 (dd, ^3^*J*_3,4_ = 9.5 Hz, ^3^*J*_2,3_ = 3.5 Hz, 1H, H-3_Man_), 3.77–3.68 (m, 3H, H-4_Man_, H-6a_Man_, H-6b_Man_), 3.61–3.53 (m, 3H, H-5_Man_, H-3_Xylose_, H-4_Xylose_), 3.42–3.36 (m, 1H, H-2_Xylose_), 3.22 (br dd, ^2^*J*_5ax,5eq_ = 11.2 Hz, ^3^*J*_4,5ax_ = 9.7 Hz, 1H, H-5ax_Xylose_), 3.15 (t, ^3^*J*_CH2,CH2_ = 7.2 Hz, 2H, CH_2_-CH_2_-N), 1.49–1.32 (m, 18H, 2 × Boc) ppm; ^13^C NMR (151 MHz, CD_3_OD, 298 K): δ = 158.1 (C=O_Boc_), 157.0 (C*_ipso_*_-Phenol_), 146.7 (C=CH_Triazole_), 132.5 (C*_p_*_-Phenol_), 130.9 (C*_m_*_-Phenol_), 124.8 (C=CH_Triazole_), 118.1 (C*_o_*_-Phenol_), 101.0 (C-1_Xylose_), 100.4 (C-1_Man_), 80.8 (C-3_Xylose_), 80.4 (2 × C_q_ Boc), 75.4 (C-5_Man_), 72.5 (C-3_Man_), 72.1 (C-2_Man_), 68.4 (C-4_Man_), 66.4 (N-C-CH_2 at C-3_), 64.9 (C-5_Xylose_), 62.7 (C-6_Man_), 57.3 (C-2_Xylose_), 56.3 (N-C-CH2_at C-1_), 53.0 (C-4_Xylose_), 52.9 (CH_2_-N), 36.8 (CH_2_-CH_2_-N), 28.9 (Boc), 28.8 (Boc) ppm; IR (ATR): *ν̃* = 3345, 2980, 2936, 1688, 1512, 1163, 1061, 1009, 864 cm^−1^; ESI-HRMS: *m/z* = 748.33777 [C_35_H_51_N_5_O_13_ − H]^−^ (calculated *m/z* = 748.33996 [C_35_H_51_N_5_O_13_ − H]^−^).

*Glycoconjugate* **20**. The protected glycoconjugate **19** (36.4 mg, 25.8 µmol) was suspended in methanol (3.0 mL) and sodium methoxide (5.4 m in methanol, 15 µL, 81 µmol) was added dropwise. The reaction mixture was stirred at room temperature for 85 min. It was neutralised by addition of Amberlite^®^-IRC 120 H^+^ resin. The resin was filtered off and the filtrate was concentrated under reduced pressure. The crude product was purified on reversed phase silica gel by automated flash chromatography (H_2_O:MeCN, 100:0 → 0:100) to obtain the title compound **20** after lyophilisation (25.8 mg, 24.0 µmol, 93%); $[\alpha {]}_{D}^{20}$ = 39.342 (c = 0.1398, MeOH); ^1^H NMR (600 MHz, CD_3_OD, 298 K): δ = 7.73 (s, 1H, C=CH_Triazole at C-1_), 7.69 (s, 1H, C=CH_Triazole at C-3_), 7.08–6.99 (m, 8H, 2 × CH*_m_*_-Phenol_, 2 × CH*_o_*_-Phenol_), 5.45–5.41 (m, 2H, 2 × H-1_Man_), 4.84–4.61 (m, 4H, N-C-CH2 _at C-1_, N-C-CH2 _at C-3_), 4.61–4.54 (m, 4H, 2 × CH_2_-N), 4.47 (br d, ^3^*J*_1,2_ = 7.2 Hz, 1H, H-1_Xylose_), 4.00–3.97 (m, 2H, 2 × H-2_Man_), 3.95–3.86 (m, 3H, H-5eq_Xylose_,2 × H-3_Man_), 3.77–3.68 (m, 6H,4 × H-6_Man_, 2 × H-4_Man_), 3.63–3.56 (m, 3H, H-4_Xylose_, 2 × H-5_Man_), 3.52 (br t, ^3^*J*_2,3_ = ^3^*J*_3,4_ = 8.9 Hz, 1H, H-3_Xylose_), 3.46–3.41 (m, 1H, H-2_Xylose_), 3.23 (br dd, ^2^*J*_5eq.5ax_ = 11.6 Hz ^3^*J*_4,5ax_ = 10.0 Hz 1H, H-5a_Xylose_), 3.14 (t, ^3^*J*_CH2,CH2_ = 7.2 Hz, 4H, 2 × CH_2_-CH_2_-N), 1.41 (s, 9H, Boc), 1.37 (s, 9H, Boc) ppm; ^13^C NMR (151 MHz, CD_3_OD, 298 K): δ = 157.9, 157.0 (2 × C*_ipso_*_-Phenol_), 146.7 (C=CH_Triazole at C-3_), 145.5 (C=CH_Triazole at C-1_), 132.5 (2 × C*_p_*_-Phenol_), 130.9 (2 × C*_m_*_-Phenol_), 125.4 (C=CH_Triazole at C-1_), 124.8 (C=CH_Triazole at C-3_), 118.1 (2 × C*_o_*_-Phenol_), 102.3 (C-1_Xylose_), 100.3 (2 × C-1_Man_), 80.7 (C-3_Xylose_), 80.5 (C*_q_* Boc), 80.3 (C*_q_* Boc), 75.3 (2 × C-5_Man_), 72.4 (2 × C-3_Man_), 72.0 (2 × C-2_Man_), 68.4 (2 × C-4_Man_), 66.2 (N-C-CH_2 at C-3_), 64.8 (C-5_Xylose_), 62.7 (N-C-CH_2 at C-1_, C-6_Man_), 57.2 (C-2_Xylose_), 52.9 (2 × CH_2_-N, C-4_Xylose_), 36.7 (CH_2_-CH_2_-N), 28.82 (Boc), 28.76 (Boc) ppm. IR (ATR): *ν̃* = 3341, 2936, 1692, 1511, 1230, 1058, 1009, 826 cm^−1^; ESI-HRMS: *m/z* = 1075.48357 [C_49_H_70_N_8_O_19_ + H]^+^ (calculated *m/z* = 1075.48300 [C_49_H_70_N_8_O_19_ + H]^+^).

*Glycoconjugate* **24**. The protected glycoconjugate **23** (34.9 mg, 21.0 µmol) was suspended in dry methanol (2.0 mL) under a nitrogen atmosphere and sodium methoxide (5.4 m in methanol, 10 µL, 54 µmol) was added dropwise. The reaction mixture was stirred at room temperature for 5.5 h. It was neutralised by addition of Amberlite^®^-IRC 120 H^+^ resin. The resin was filtered off and the filtrate was concentrated under reduced pressure. The crude product was purified on reversed phase silica gel by automated flash chromatography (H_2_O:MeCN, 100:0 → 0:100) to obtain the title compound **24** after lyophilisation (16.7 mg, 15.5 µmol, 74%); $[\alpha {]}_{D}^{20}$ = −5.888 (c = 0.1019, MeOH); ^1^H NMR (600 MHz, CD_3_OD, 298 K): δ = 7.73 (s, 1H, C=CH_Triazole at C-1_), 7.69 (s, 1H, C=CH_Triazole at C-3_), 7.08–7.98 (m, 8H, 2 × CH*_m_*_-Phenol_, 2 × CH*_o_*_-Phenol_), 5.43 (d, ^3^*J*_1,2_ = 1.8 Hz, 1H, H-1_Man_), 4.92–4.77 (m, 2H, H-1_Glc_, N-C-CHH′ _at C-1_), 4.76–4.74 (m, 2H, N-C-CH_2 at C-3_), 4.64 (d, ^2^*J*_CHH′,CHH′_= 12.4 Hz, 1H, N-C-CHH′_at C-1_), 4.60–4.53 (m, 4H, 2 × CH_2_-N), 4.47 (br d, ^3^*J*_1,2_ = 7.1 Hz, 1H, H-1_Xylose_), 3.99 (dd, ^3^*J*_2,3_ = 3.4 Hz, ^3^*J*_1,2_ = 1.8 Hz, 1H, H-2_Man_), 3.94–3.86 (m, 3H, H-5eq_Xylose_, H-6a_Man_, H-3_Man_), 3.77–3.67 (m, 4H, H-4_Man_, H-6a_Glc_, H-6b_Glc_, H-6b_Man_), 3.63–3.56 (m, 2H, H-4_Xylose_, H-5_Man_), 3.52 (br t, ^3^*J*_2,3_ = ^3^*J*_3,4_ = 8.6 Hz, 1H, H-3_Xylose_), 3.48–3.35 (m, 5H, H-2_Xylose_, H-2_Glc_, H-3_Glc_, H-4_Glc_, H-5_Glc_), 3.23 (br dd, ^2^*J*_5eq,5ax_ = 11.3 Hz, ^3^*J*_4,5ax_ = 9.9 Hz, 1H, H-5ax_Xylose_), 3.14 (t, ^3^*J*_CH2,CH2_ = 7.2 Hz, 4H, 2 × CH_2_-CH_2_-N), 1.42 (s, 9H, Boc), 1.37 (s, 9H, Boc) ppm; ^13^C NMR (151 MHz, CD_3_OD, 298 K): δ = 158.1 (C*_ipso_*_-Phenol at Glc_), 157.9, 157.0 (C*_ipso_*_-Phenol at Man_), 146.6 (C=CH_Triazole at C-3_), 145.5 (C=CH_Triazole at C-1_), 132.5 (C*_p_*_-Phenol_), 130.9 (C*_m_*_-Phenol_), 130.8 (C*_m_*_-Phenol_), 125.4 (C=CH_Triazole at C-1_), 124.8 (C=CH_Triazole at C-3_), 118.1 (C*_o_*_-Phenol_), 118.0 (C*_o_*_-Phenol_), 102.34 (C-1_Glc_), 102.25 (C-1_Xylose_), 100.3 (C-1_Man_), 80.8 (C-3_Xylose_), 79.1 (C_q_ Boc), 78.9 (C_q_ Boc), 78.1 (C-5_Glc_), 78.0 (C-2_Glc_), 75.3 (C-5_Man_), 74.9 (C-4_Glc_), 72.4 (C-3_Man_), 72.0 (C-2_Man_), 71.4 (C-3_Glc_), 68.4 (C-4_Man_), 66.3 (N-C-CH_2 at C-3_), 64.7 (C-5_Xylose_), 62.7 (N-C-CH_2 at C-1_, C-6_Glc_), 62.5 (C-6_Man_), 57.1 (C-2_Xylose_), 52.9 (CH_2_-N, C-4_Xylose_), 36.7 (CH_2_-CH_2_-N), 28.82 (Boc), 28.72 (Boc) ppm; IR (ATR): *ν̃* = 3344, 2932, 1694, 1512, 1234, 1070, 1017 cm^−1^; ESI-HRMS: *m/z* = 1075.48312 [C_49_H_70_N_8_O_19_ + H]^+^ (calculated *m/z* = 1075.48300 [C_49_H_70_N_8_O_19_ + H]^+^).

*Glycoconjugate* **26**. The protected glycocluster **25** (53.3 mg, 43.1 µmol) was dissolved in dichloromethane (3.0 mL) and methanol (3.0 mL) was added. The mixture was stirred at room temperature and sodium methoxide (5.4 m in methanol, 17.5 µL, 94.5 µmol) was added dropwise and a precipitate was observed. After 5 min additional methanol (3.0 mL) was added followed by dropwise sodium methoxide (5.4 m in methanol, 12.5 µL, 67.5 µmol). The mixture was further stirred at room temperature for 1 h 35 min. It was neutralised by addition of Amberlite^®^-IRC 120 H^+^ resin. The resin was filtered off and the filtrate was concentrated under reduced pressure. The crude product was purified on reversed phase silica gel by automated flash chromatography (H_2_O:MeCN, 100:0 → 0:100) to obtain the title compound **26** after lyophilisation (35.9 mg, 39.8 µmol, 92%); $[\alpha {]}_{D}^{20}$ = +20.214 (c = 0.1682, H_2_O); ^1^H NMR (600 MHz, D_2_O, 298 K): δ = 7.62 (s, 1H, C=CH_Triazole at C-1_), 7.46 (s, 1H, C=CH_Triazole at C-3_), 7.00–6.85 (m, 8H, 4 × CH*_m_*_-Phenol_, 4 × CH*_o_*_-Phenol_), 5.45 (d, ^3^*J*_1,2_ = 1.8 Hz, 1H, H-1_Man_), 5.40 (d, ^3^*J*_1,2_ = 1.9 Hz, 1H, H-1_Man_), 4.80–4.77 (m, 1H, H-1_Xylose_), 4.71–4.55 (m, 8H, 2 × CH_2_-N, 2 × N-C-CH_2_), 4.07 (dd, ^3^*J*_2,3_ = 3,5 Hz, ^3^*J*_1,2_ = 1.8 Hz, 1H, H-2_Man_), 4.05 (dd, ^3^*J*_2,3_ = 3,4 Hz, ^3^*J*_1,2_ = 1.8 Hz, 1H, H-2_Man_), 3.99–3.90 (m, 4H, H-3_Xylose_, H-5a_Xylose_, H-3_Man_, H-3_Man_), 3.74–3.67 (m, 6H, H-4_Man_, H-4_Man_, H-6a_Man_, H-6a′_Man_, H-6b_Man_, H-6b′_Man_), 3.63–3.59 (m, 2H, H-5_Man_, H-5_Man_), 3.53–3.51 (m, 1H, H-2_Xylose_), 3.45–3.43 (m, 1H, H-4_Xylose_), 3.40–3.36 (m, 1H, H-5b_Xylose_), 3.14 (t, ^3^*J*_CH2,CH2_ = 6.5 Hz, 4H, 2 × CH_2_-CH_2_-N) ppm; **^13^**C NMR (151 MHz, D_2_O, 298 K): δ = 159.0 (C=O), 154.24 (C*_ipso_*_-Phenol_), 154.17 (C*_ipso_*_-Phenol_), 143.9 (C=CH_Triazole at C-1_), 143.6 (C=CH_Triazole at C-3_), 131.8 (2 × C*_p_*_-Phenol_), 129.92 (C*_m_*_-Phenol_), 129.89 (C*_m_*_-Phenol_), 125.14 (C=CH_Triazole at C-1_), 125.07 (C=CH_Triazole at C-3_), 117.2 (C*_o_*_-Phenol_), 117.1 (C*_o_*_-Phenol_), 100.0 (C-1_Xylose_), 98.3 (C-1_Man_), 98.2 (C-1_Man_), 73.3 (2 × C-5_Man_), 70.4 (2 × C-3_Man_), 69.9 (2 × C-2_Man_), 68.8 (C-3_Xylose_), 66.5 (2 × C-4_Man_), 61.4 (N-C-CH_2 at C-3_), 60.7 (N-C-CH_2 at C-1_), 60.6 (2 × C-6_Man_), 60.1 (C-5_Xylose_), 51.7 (CH_2_-N), 51.5 (CH_2_-N), 46.8 (C-2_Xylose_), 46.4 (C-4_Xylose_), 34.8 (CH_2_-CH_2_-N), 34.7 (CH_2_-CH_2_-N) ppm; IR (ATR): *ν̃* = 3327, 2931, 1649, 1510, 1223, 1011, 824 cm^−1^; ESI-HRMS: *m/z* = 901.35790 [C_40_H_52_N_8_O_16_ + H]^+^ (calculated *m/z* = 901.35740 [C_40_H_52_N_8_O_16_ + H]^+^).

## 4. Conclusions

Conformational glycoswitches represent a powerful class of glycomimetics that enable facile modulation of the spatial orientation of multiple carbohydrate ligands. Xylosides, for example, can function as a molecular hinge through interconversion between the two chair conformations, ^4^*C*_1_ and ^1^*C*_4_. In this work, we have established a robust synthetic route to a clickable xyloside scaffold bearing two alkyne moieties that can be addressed orthogonally in subsequent CuAAC reactions. This strategy provides straightforward access to a broad range of homo- and heterobivalent glycoclusters via modular click-chemical assembly of ligands and spacers.

Notably, even in systems incorporating long and relatively flexible spacers, the chair conformation of the central xyloside scaffold exerts a pronounced influence on the ligand properties of the resulting glycoclusters, as demonstrated in adhesion–inhibition assays with *E. coli*. It should be emphasised, however, that these experiments are only preliminary and should be supplemented by further studies. The principal value of the present work lies in the highly modular and flexible variation of ligands and spacers, which enables the investigation of the effects of altered spatial organisation in virtually any test system or for any lectin. Our findings with the clickable xyloside scaffold also suggest that the incorporation of more rigid spacers between the scaffold and glycoligands will further enhance the control of spatial orientation and, consequently, advance our knowledge of the boundary conditions of carbohydrate recognition.

## Figures and Tables

**Table 1:** Inhibitory potencies of glycoclusters **18**, **20**, **24**, and **26** determined in adhesion–inhibition assays with type 1-fimbriated *E. coli* (PKL1162).

Glycocluster	IC_50_ ^a^ (µM)	IC_50_ MeMan ^b^ (mM)	RIP ^c^	Average RIP ^d^
**18** (Man; ^4^*C*_1_)	24.3 (±5.1)	5.2 (±0.6)	213.4 (±53.3)	209.9 (±33.2)
	12.9 (±2.4)	2.7 (±0.5)	206.4 (±39.6)	
**20** (Man/Man; ^4^*C*_1_)	33.2 (±3.5)	3.8 (±0.2)	114.7 (±13.6)	118.7 (±10.5)
	53.3 (±9.3)	7.4 (±1.0)	138.4 (±26.4)	
	43.6 (±1.6)	4.5 (±0.3)	103.0 (±10.3)	
**24** (Glc/Man; ^4^*C*_1_)	229.3 (±44.5)	21.7 (±1.1)	94.7 (±23.0)	87.1 (±11.4)
	35.8 (±5.0)	3.8 (±0.2)	106.6 (±15.8)	
	250.3 (±9.8)	15.1 (±1.3)	60.1 (±19.7)	
**26** (Man/Man; ^1^*C*_4_)	23.6 (±2.9)	5.2 (±0.6)	219.6 (±29.0)	201.9 (±18.0)
	19.8 (±2.8)	2.7 (±0.5)	134.1 (±22.6)	
	17.8 (±3.1)	4.5 (±0.3)	252.1 (±39.7)	

^a^ IC_50_ values are average values of duplicate or triplicate measurements from a single plate; fitting errors are given in brackets. ^b^ IC_50_ (MeMan) values are average values of triplicate measurements from a single plate; fitting errors are given in brackets. ^c^ RIP values are based on the inhibitory potency of MeMan tested on the same microplate (IP(MeMan) ≡ 1); RIP (glycocluster) = IC_50_ (MeMan)/IC_50_ (glycocluster). The fitting error from error propagation is given in brackets (see Supplementary Materials Section S3 for details). ^d^ Average RIPs are mean values from two to three independent experiments with error propagation in brackets (see Supplementary Materials Section S3).

## Data Availability

The data supporting this article have been included as part of the Supplementary Materials, which contains additional references [36,37,38,39,40,41,42].

## Supplementary Materials

The following supporting information can be downloaded at https://www.mdpi.com/article/10.3390/molecules31142432/s1, Figure S1: ^1^H NMR spectra of compound **7** recorded at different temperatures. Figure S2: ^1^H NMR spectra of compound **7** in the region where the H-3 proton is located. Figure S3: Representative inhibition curves obtained from the evaluation of glycoclusters **18**, **20**, **24**, and **26** as inhibitors of type 1 fimbriae-mediated bacterial adhesion to mannan. Methyl α-d-mannopyranoside (MeMan) was included as a reference inhibitor on each plate. Sigmoidal concentration–response curves were obtained by nonlinear regression analysis. Error bars represent standard deviations of triplicate or duplicate determinations performed on a single plate. Synthetic procedures, if not mentioned in the main manuscript, are reported as well as the ^1^H NMR and ^13^C NMR spectra (Figures S4–S55) of the synthesised compounds.
