# Supplementary material for: An Orthogonal ‘Clickable’ Xyloside Scaffold in 4C1 and 1C4 Conformation
Source: Molecules. 2026 Jul 11;31(14):2432. doi: 10.3390/molecules31142432 (PMC13414126; doi:10.3390/molecules31142432)
Supplement: Supplementary file 1 [file molecules-31-02432-s001.zip › molecules-4364051-supplementary.pdf]

Electronic Supplementary Information (ESI)

for

An orthogonal 'clickable' xyloside scaffold in $^{4}C_{1}$ and $^{1}C_{4}$ conformation

Lorenz Pietsch, Sönke Sdunnus and Thisbe K. Lindhorst*

Otto Diels Institute of Organic Chemistry, Christiana Albertina University of Kiel, Otto-Hahn-Platz 3-4, 24118 Kiel, Germany

Table of contents

1 Synthetic procedures 3

1.1 Methyl 2,3-*O*-isopropylidene-β-D-xylopyranoside (**S2**) 3

1.2 Methyl 3,4-anhydro-α-L-arabinopyranoside (**S4**) 3

1.3 Methyl 3,4-anhydro-2-*O*-methanesulfonyl-α-L-arabinopyranoside (**S5**) 4

1.4 Methyl 2,4-diazido-2,4-dideoxy-β-D-xylopyranoside (**S6**) and methyl 3,4-diazido-3,4-dideoxy-β-D-arabinopyranoside (**S7**) 5

1.5 1,3-Di-*O*-acetyl-2,4-diazido-2,4-dideoxy-α-β-D-xylopyranose (**1**) 6

1.6 3-(Triisopropylsilyl)propargyl 3-*O*-acetyl-2,4-diazido-2,4-dideoxy-D-xylopyranoside (**2**) 7

1.7 3-(Triisopropylsilyl)propargyl 2,4-diazido-2,4-dideoxy-D-xylopyranoside (**3**) 8

1.8 3-(Triisopropylsilyl)propargyl 2,4-dideoxy-2,4-*N*-Boc-α-D-xylopyranoside (**4**) and 3-(Triisopropylsilyl)propargyl 2,4-dideoxy-2,4-*N*-Boc-β-D-xylopyranoside (**5**) 9

1.9 3-(Triisopropylsilyl)propargyl 2,4-dideoxy-2,4-*N*-Boc-3-*O*-propargyl-α-D-xylopyranoside (**6**) 10

1.10 3-(Triisopropylsilyl)propargyl 2,4-dideoxy-2,4-*N*-Boc-3-*O*-propargyl-β-D-xylopyranoside (**7**) 11

1.11 3-(Triisopropylsilyl)propargyl 2,4-*N*-carbonyl-2,4-diamino-2,4-dideoxy-3-*O*-propargyl-α-D-xylopyranoside (**8**) 12

1.12 3-(Triisopropylsilyl)propargyl 2,4-*N*-carbonyl-2,4-diamino-2,4-dideoxy-3-*O*-propargyl-β-D-xylopyranoside (**9**) 13

1.13 Propargyl 2,4-*N*-carbonyl-2,4-diamino-2,4-dideoxy-3-*O*-propargyl-β-D-xylopyranoside (**10**) 14

1.14 4-(2-Azidoethyl)phenyl 2,3,4,6-tetra-*O*-benzoyl-β-D-glucopyranoside (**15**) 15

1.15 Glycoconjugate **16** 16

1.16 Glycoconjugate **17** 17

1.17 Glycoconjugate **19** 18

1.18 Glycoconjugate **21** 19

1.19 Glycoconjugate **22** 20

1.20 Glycoconjugate **23** 21

1.21 Glycoconjugate **25** 23

2 Conformational analysis 25

3 Biological testing 27

4 NMR Spectra 31

4.1 Compound **S2** 31

4.2 Compound **S4** 32

4.3 Compound **S5** 33

4.4 Compound **S6** 34

4.5 Compound **S7** 35

4.6 Compound **2** ... 36
4.7 Compound **3** ... 37
4.8 Compound **4** ... 38
4.9 Compound **5** ... 39
4.10 Compound **6** ... 40
4.11 Compound **7** ... 41
4.12 Compound **8** ... 42
4.13 Compound **9** ... 43
4.14 Compound **10** ... 44
4.15 Compound **15** ... 45
4.16 Glycoconjugate **16** ... 46
4.17 Glycoconjugate **17** ... 47
4.18 Glycoconjugate **18** ... 48
4.19 Glycoconjugate **19** ... 49
4.20 Glycoconjugate **20** ... 50
4.21 Glycoconjugate **21** ... 51
4.22 Glycoconjugate **22** ... 52
4.23 Glycoconjugate **23** ... 53
4.24 Glycoconjugate **24** ... 54
4.25 Glycoconjugate **25** ... 55
4.26 Glycoconjugate **26** ... 56

5 References ... 57

1 Synthetic procedures

In the following, the synthetic procedures for the new compounds are provided. Compounds **S2**-**S7** were prepared according to the synthetic routes published by Yuasa and Hashimoto [1] and Helm, Ralph and Anderson [2], with slight modifications The characterisation of previously reported compounds is provided for convenience of the reader.

1.1 Methyl 2,3-*O*-isopropylidene-β-D-xylopyranoside (S2)

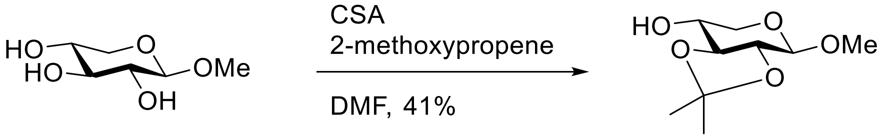

The xyloside **S2** has been previously reported in the literature [2]. The synthetic procedure used here is based on a combination of two reported methods.[2,3] Methyl β-D-xylopyranoside (**S1**) (2.00 g, 12.2 mmol) and camphor-10-sulfonic acid were dissolved in dry dimethylformamide (20 mL) under a nitrogen atmosphere. The mixture was stirred at 60 °C and 2-methoxypropene (3.5 mL, 36.5 mmol) was added dropwise. After stirring for 1 h the mixture was cooled to room temperature and triethylamine (1.0 mL) was added. The solvent was removed under reduced pressure. The crude syrup was coevaporated with toluene (2 x 20 mL). The crude product was dissolved in dichloromethane and loaded onto Celite® and purified on silica gel (cyclohexane:ethyl acetate, 90:10→60:40) to obtain the product **S2** with minor impurities as a colourless syrup (1.01 g, 4.95 mmol, 41%).

ESI-HRMS: *m/z* = 205.10674 $[C_9H_{16}O_5+H]^+$ (calculated *m/z* = 205.10705 $[C_9H_{16}O_5+H]^+$).

^{1}H NMR (600 MHz, $CDCl_3$, 298 K): δ = 4.54 (d, $^3J_{1,2}$ = 7.6 Hz, 1H, H-1), 4.07 (dd, $^2J_{5ax,5eq}$ = 11.6 Hz, $^3J_{4,5eq}$ = 5.3 Hz, 1H, H-5$_{eq}$), 4.06-4.00 (m, 1H, H-4), 3.56-3.50 (m, 1H, H-3), 3.54 (s, 3H, OC<u>H</u>$_3$), 3.32 (dd, $^3J_{2,3}$ = 9.5 Hz, $^3J_{1,2}$ = 7.6 Hz, 1H, H-2), 3.26 (dd, $^2J_{5ax,5eq}$ = 11.6 Hz, $^3J_{4,5ax}$ = 8.1 Hz, 1H, H-5$_{ax}$), 2.35 (d, $^3J_{4,OH}$ = 3.6 Hz, 1H, O<u>H</u>), 1.48-1.44 (m, 6H, C(C<u>H</u>$_3$)$_2$) ppm.

^{13}C NMR (151 MHz, $CDCl_3$, 298 K): δ = 111.9 (<u>C</u>(CH$_3$)$_2$), 102.9 (C-1), 81.1 (C-3), 76.6 (C-2), 69.4 (C-4), 67.4 (C-5), 56.8 (OC<u>H</u>$_3$), 26.9 (C(<u>C</u>H$_3$)$_2$), 26.7 (C(<u>C</u>H$_3$)$_2$) ppm.

1.2 Methyl 3,4-anhydro-α-L-arabinopyranoside (S4)

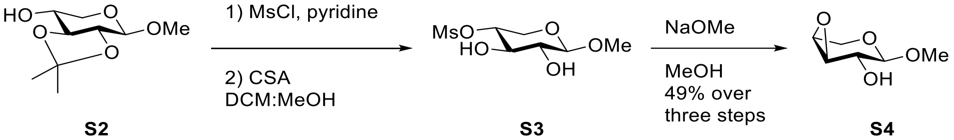

Following an adapted procedure from Yuasa and Hashimoto [1], methyl 2,3-*O*-isopropylidene-β-D-xylopyranoside (**S2**) (1.71 g, 8.37 mmol) was dissolved in dry pyridine (14.5 mL) under a nitrogen atmosphere. The mixture was stirred at room temperature and methanesulfonyl chloride (960 µL, 12.4 mmol) was added dropwise. After stirring for 4 h water (150 mL) was added. The mixture was extracted with dichloromethane (4 x 25 mL). The combined organic layers were dried over $MgSO_4$, it was filtered and the solvent removed under reduced pressure.

The crude syrup was coevaporated with toluene (2 x 10 mL). The crude product (2.45 g) was dissolved in dichloromethane (16 mL) followed by addition of methanol (16 mL). The mixture was stirred at room temperature and camphorsulfonic acid (620 mg) was added carefully. The mixture was further stirred for 30 min followed by addition of triethyl amine (1 mL). The solvent was removed under reduced pressure and the crude product **S3** was loaded onto Celite® and purified on silica gel (cyclohexane:acetone, 4:1→1:3) to obtain the product **S3** with minor impurities (1.41 g).

S3 (1.39 g) was dissolved in dry methanol (54 mL) under a nitrogen atmosphere. The mixture was stirred at 0 °C and NaOMe in methanol (1.16 mL, 5.4 M, 6.3 mmol) was added dropwise. After stirring for 2 h at room temperature acetic acid (340 µL) was added dropwise followed by toluene (15 mL). The solvent was removed under reduced pressure and the crude product was loaded onto Celite® and purified on silica gel (cyclohexane:acetone, 90:10→35:65) to obtain the product **S4** as a colourless solid (603 mg, 4.13 mmol, 49%).

ESI-HRMS: *m/z* = 164.09163 $[C_6H_{10}O_4+NH_4]^+$ (calculated *m/z* = 164.09173 $[C_6H_{10}O_4+NH_4]^+$).

^{1}H NMR (600 MHz, $CDCl_3$, 298 K): δ = 4.24 (d, $^2J_{5ax,5eq}$ = 13.3 Hz, 1H, H-5_{eq}), 4.02 (d, $^3J_{1,2}$ = 6.7 Hz, 1H, H-1), 3.93 (dd, $^2J_{5ax,5eq}$ = 13.5 Hz, $^3J_{5ax,4}$ = 1.2 Hz 1H, H-5_{ax}), 3.70 (dd, $^3J_{1,2}$ = 6.7 Hz, $^3J_{2,OH}$ = 4.0 Hz, 1H, H-2), 3.48 (s, 3H, $OC\underline{H}_3$), 3.25-3.23 (m, 1H, H-3), 3.18-3.15 (m, 1H, H-4), 2.40 (d, $^3J_{2,OH}$ = 4.0 Hz, 1H, $O\underline{H}$) ppm.

^{13}C NMR (151 MHz, $CDCl_3$, 298 K): δ = 103.8 (C-1), 67.0 (C-2), 63.0 (C-5), 56.7 ($O\underline{C}H_3$), 53.9 (C-3), 49.8 (C-4) ppm.

1.3 Methyl 3,4-anhydro-2-*O*-methanesulfonyl-α-L-arabinopyranoside (S5)

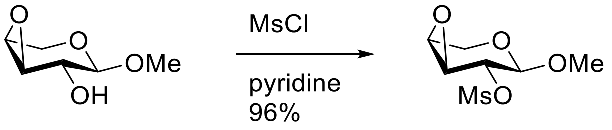

S4 **S5**

Following an adapted procedure from Yuasa and Hashimoto [1], methyl 3,4-anhydro-α-L-arabinopyranoside **S4** (587 mg, 4.02 mmol) was dissolved in dry pyridine (12.0 mL) under a nitrogen atmosphere. The mixture was stirred at room temperature and methanesulfonyl chloride (380 µL, 4.91 mmol) was added dropwise. After stirring for 7 h, methanol (1.0 mL) was added and the mixture was further stirred for 30 min. The mixture was diluted with toluene (10 mL) and the solvent was removed under reduced pressure. The crude product (2.45 g) was dissolved in dichloromethane and loaded onto Celite® and purified on silica gel (cyclohexane:ethyl acetate, 3:1→3:2) to obtain the product **S5** as a colourless solid (865 mg, 3.86 mmol, 96%).

ESI-HRMS: *m/z* = 242.06926 $[C_7H_{12}O_6S+NH_4]^+$ (calculated *m/z* = 242.06928 $[C_7H_{12}O_6S+NH_4]^+$).

^{1}H NMR (600 MHz, $CDCl_3$, 298 K): δ = 4.50 (d, $^3J_{1,2}$ = 6.8 Hz, 1H, H-2), 4.27 (d, $^2J_{5ax,5eq}$ = 13.7 Hz, 1H, H-5a), 4.21 (d, $^3J_{1,2}$ = 6.8 Hz, 1H, H-1), 3.96 (dd, $^2J_{5ax,5eq}$ = 13.6 Hz, $^3J_{5ab,4}$ = 1.0 Hz, 1H, H-5b), 3.07 (s, 3H, $OC\underline{H}_3$), 3.44-3.42 (m, 1H, H-3), 3.22-3.20 (m, 1H, H-4), 3.10 (s, 3H, $C\underline{H}_3SO_2$) ppm.

^{13}C NMR (151 MHz, $CDCl_3$, 298 K): δ = 100.4 (C-1), 74.0 (C-2), 62.8 (C-5), 56.6 (O<u>C</u>H_3), 52.6 (C-3), 49.3 (C-4), 38.4 (<u>C</u>H_3SO_2) ppm.

1.4 Methyl 2,4-diazido-2,4-dideoxy-β-D-xylopyranoside (S6) and methyl 3,4-diazido-3,4-dideoxy-β-D-arabinopyranoside (S7)

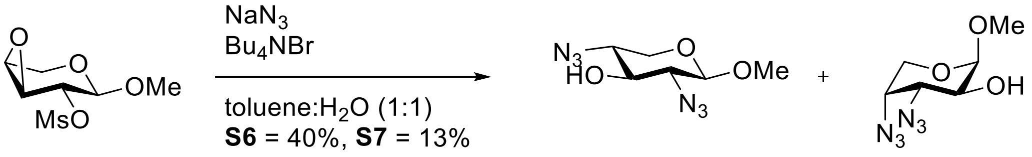

S5 **S6** **S7**

Following an adapted procedure from Yuasa and Hashimoto [1], the arabinoside **S5** (500 mg, 2.23 mmol), sodium azide (1.25 g, 19.2 mmol) and tetrabutylammonium bromide (106 mg, 329 mol) were dissolved in a mixture of toluene (11 mL) and water (11 mL) in a pressure tube (50 mL). The mixture was stirred in the closed pressure tube at 120 °C for 20 h. The reaction mixture was allowed to reach room temperature and was diluted with toluene (50 mL) and a half satd. brine solution (50 mL). The phases were separated and the aqueous layer was extracted with dichloromethane (2 x 10 mL). The combined organic layers were dried over $MgSO_4$, it was filtered and concentrated. The residue was diluted with dichloromethane and loaded onto Celite®. The crude product was purified on silica gel (cyclohexane:acetone, 100:0 → 85:15) to obtain the title compound **S6** as a colourless solid (191 mg, 892 µmol, 40%) and the side product **S7** as colourless fibre (63.8 mg, 298 µmol, 13%).

S6

$\boldsymbol{R}_F$ (cyclohexane:ethyl acetate, 3:1) = 0.33.

^{1}H NMR (600 MHz, $CDCl_3$, 298 K): δ = 4.15 (d, $^3J_{1,2}$ = 7.8 Hz, 1H, H-1), 4.01 (dd, $^2J_{5ax,5eq}$ = 11.9 Hz, $^3J_{4,5eq}$ = 5.4 Hz, 1H, H-5_{eq}), 3.58-3.52 (m, 1H, H-4), 3.56 (s, 3H, C<u>H</u>$_3$), 3.40 (td, $^3J_{2,3}$ = $^3J_{3,4}$ = 9.5 Hz, $^3J_{3,OH}$ = 3.4 Hz, 1H, H-3), 3.29 (dd, $^3J_{2,3}$ = 9.7 Hz, $^3J_{1,2}$ = 7.8 Hz, 1H, H-2), 3.15 (dd, $^2J_{5ax,5eq}$ = 12.0 Hz, $^3J_{4,5ax}$ = 10.8 Hz, 1H, H-5_{ax}), 2.60 (d, $^3J_{3,OH}$ = 3.4 Hz, 1H, O<u>H</u>) ppm.

^{13}C NMR (151 MHz, $CDCl_3$, 298 K): δ = 103.7 (C-1), 74.4 (C-3), 66.3 (C-2), 64.0 (C-5), 61.0 (C-4), 57.4 (<u>C</u>H_3) ppm.

S7

$\boldsymbol{R}_F$ (cyclohexane:acetone, 3:1) = 0.29.

^{1}H NMR (600 MHz, $CDCl_3$, 298 K): δ = 4.78 (d, $^3J_{1,2}$ = 3.6 Hz, 1H, H-1), 3.98 (td, $^3J_{2,3}$ = $^3J_{2,OH}$ = 10.2 Hz, $^3J_{1,2}$ = 3.7 Hz, 1H, H-2), 3.86-3.82 (m, 2H, H-4, H-5a), 3.78 (dd, $^3J_{2,3}$ = 9.9 Hz, $^3J_{3,4}$ = 3.6 Hz, 1H, H-3), 3.73-3.68 (m, 1H, H-5b), 3.46 (s, 3H, OC<u>H</u>$_3$), 2.11 (d, $^3J_{2,OH}$ = 10.3 Hz, 1H, O<u>H</u>) ppm

^{13}C NMR (151 MHz, $CDCl_3$, 298 K): δ = 94.2 (C-1), 68.9 (C-2), 62.0 (C-3), 60.5 (C-5), 60.4 (C-4), 56.6 (O<u>C</u>H_3) ppm.

1.5 1,3-Di-*O*-acetyl-2,4-diazido-2,4-dideoxy-α-β-D-xylopyranose (1)

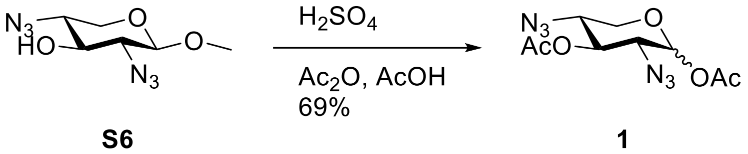

Following an adapted procedure from Yuasa and Hashimoto [1], the methyl xyloside **S6** (966 mg, 4.51 mmol) was dissolved in acetic anhydride (11.8 mL) and stirred at room temperature. Sulfuric acid dissolved in acetic acid was added dropwise (1.2 mL of a stock solution of sulfuric acid (100 µL) dissolved in acetic acid (4.0 mL)). After stirring for 73 min sodium bicarbonate (438 mg, 5.21 mmol) was added followed by methanol (9.0 mL). The mixture was stirred for 30 min at room temperature and was then diluted with dichloromethane (100 mL) and brine (100 mL). The phases were separated and the aqueous layer was extracted with dichloromethane (20 mL). The combined organic layers were dried over $MgSO_4$, it was filtered and the filtrate concentrated. The residue was coevaporated with toluene (3 x 30 mL) and afterwards diluted with dichloromethane and loaded onto Celite®. The crude product was purified on silica gel (cyclohexane:ethyl acetate, 95:5 → 85:15) to obtain the title compound **1** as a colourless syrup (886 mg, 3.12 mmol, 69%).

$\boldsymbol{R}_F$ (cyclohexane:ethyl acetate, 3:1) = 0.48.

ESI-HRMS: *m/z* = 302.12063 $[C_9H_{12}N_6O_5+NH_4]^+$ (calculated *m/z* = 302.12074 $[C_9H_{12}N_6O_5+NH_4]^+$).

1α 82%

^{1}H NMR (600 MHz, $CDCl_3$, 298 K): δ = 6.22 (d, $^3J_{1,2}$ = 3.6 Hz, 1H, H-1), 5.42-5.38 (m, 1H, H-3), 3.89-3.84 (m, 1H, H-5$_{eq}$), 3.73-3.67 (m, 1H, H-4), 3.64-3.59 (m, 1H, H-5$_{ax}$), 3.45 (dd, $^3J_{2,3}$ 10.4 Hz, $^3J_{1,2}$ = 3.6 Hz, 1H, H-2), 2.21 (s, 3H, C$\underline{H}3_{\text{OAc at C-3}}$), 2.19 (s, 3H, C$\underline{H}3_{\text{OAc at C-1}}$) ppm.

^{13}C NMR (151 MHz, $CDCl_3$, 298 K): δ = 169.9 (C=O$_{\text{OAc at C-3}}$), 168.9 (C=O$_{\text{OAc at C-1}}$), 90.7 (C-1), 71.0 (C-3), 61.7 (C-5), 60.6 (C-2), 59.4 (C-4), 21.1 (OAc), 20.9 (OAc) ppm.

1β 18%

^{1}H NMR (600 MHz, $CDCl_3$, 298 K): δ = 5.46 (d, $^3J_{1,2}$ = 8.4 Hz, 1H, H-1), 5.02 (t, $^3J_{2,3}$ = $^3J_{3,4}$ = 10.0 Hz, 1H, H-3), 4.06 (dd, $^2J_{5ax,5eq}$ = 12.1 Hz, $^3J_{4,5eq}$ = 5.5 Hz, 1H, H-5$_{eq}$), 3.67-3.61 (m, 1H, H-4), 3.54 (dd, $^3J_{2,3}$ 10.1 Hz, $^3J_{1,2}$ = 8.4 Hz, 1H, H-2), 3.37 (dd, $^2J_{5ax,5eq}$ = 12.1 Hz, $^3J_{4,5ax}$ = 10.9 Hz, 1H, H-5$_{ax}$), 2.20 (s, 3H, C$\underline{H}3_{\text{OAc at C-3}}$), 2.18 (s, 3H, C$\underline{H}3_{\text{OAc at C-1}}$) ppm.

^{13}C NMR (151 MHz, $CDCl_3$, 298 K): δ = 169.7 (C=O$_{\text{OAc at C-3}}$), 168.7 (C=O$_{\text{OAc at C-1}}$), 93.5 (C-1), 73.0 (C-3), 64.7 (C-5), 62.9 (C-2), 59.2 (C-4), 21.0 (OAc), 20.9 (OAc) ppm.

1.6 3-(Triisopropylsilyl)propargyl 3-*O*-acetyl-2,4-diazido-2,4-dideoxy-D-xylopyranoside (2)

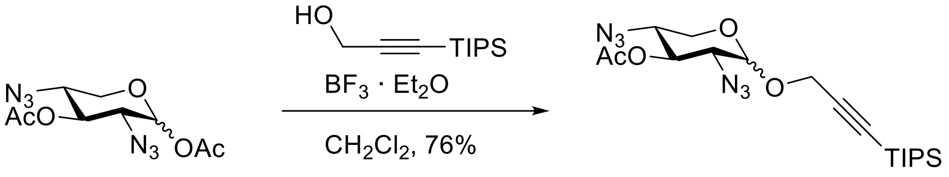

1 **2**

1,3-Di-*O*-acetyl-2,4-diazido-2,4-dideoxy-α-β-D-xylopyranose **1** (373 mg, 1.31 mmol) and 3-(triisopropylsilyl)propargyl alcohol [4] (856 mg, 4.03 mmol) were dissolved in dry dichloromethane (12.0 mL) under a nitrogen atmosphere. At 0 °C, boron trifluoride diethyl etherate (1.5 mL, 11.8 mmol) was added dropwise and the reaction mixture was further stirred at room temperature for 23 h. The reaction mixture was diluted with dichloromethane to a total volume of 100 mL and washed with a satd. aqueous $NaHCO_3$ solution (20 mL). The aqueous layer was extracted with dichloromethane (10 mL). The combined organic layers were dried over $MgSO_4$, it was filtered and the residual loaded onto Celite®. The crude product was purified on silica gel (cyclohexane:ethyl acetate, 100:0 → 85:15) to obtain the product **2** as a colourless syrup (437 mg, 1.00 µmol, 76%).

***R*$_F$** (cyclohexane:ethyl acetate, 3:1) = 0.74.

$[\alpha]_D^{20}$ = 70.329 (c = 0.27, $CHCl_3$).

IR (ATR): $\tilde{\nu}$ = 2943, 2892, 2866, 2104, 1758, 1213, 1043, 678 cm^{-1}.

ESI-HRMS: *m/z* = 454.25873 $[C_{19}H_{32}N_6O_4Si+NH_4]^+$ (calculated *m/z* = 454.25926 $[C_{19}H_{32}N_6O_4Si+NH_4]^+$).

2α 40%

^{1}H NMR (600 MHz, $CDCl_3$, 298 K): δ = 5.43-5.39 (m, 1H, H-3), 5.25 (d, $^3J_{1,2}$ = 3.4 Hz, 1H, H-1), 4.36 (d, $^2J_{C\underline{H}H',CH\underline{H}'}$ = 16.1 Hz, 1H, CH<u>H</u>'C≡C), 4.33 (d, $^2J_{C\underline{H}H',CH\underline{H}'}$ = 16.1 Hz, 1H, C<u>H</u>H'C≡C), 3.78-3.73 (m, 1H, H-5a), 3.65-3.58 (m, 2H, H-4, H-5b), 3.17 (dd, $^3J_{2,3}$ 10.5 Hz, $^3J_{1,2}$ = 3.5 Hz, 1H, H-2), 2.18 (s, 3H, $C\underline{H}3_{\text{OAc at C-3}}$), 1.09-1.06 (m, 21H, TIPS) ppm.

^{13}C NMR (151 MHz, $CDCl_3$, 298 K): δ = 169.9 ($C{=}O_{\text{OAc at C-3}}$), 101.1 ($CH_2\underline{C}{\equiv}C$), 96.5 (C-1), 89.5 ($CH_2C{\equiv}\underline{C}$), 70.8 (C-3), 61.1 (C-2), 60.3 (C-5), 59.9 (C-4) ,56.0 ($\underline{C}H_2C{\equiv}C$), 20.9 (OAc), 18.7 ($TIPS_{CH3}$), 11.2 ($TIPS_{CH}$) ppm.

2β 60%

^{1}H NMR (600 MHz, $CDCl_3$, 298 K): δ = 4.90 (t, $^3J_{2,3}$ = $^3J_{3,4}$ = 9.9 Hz, 1H, H-3), 4.62 (d, $^3J_{1,2}$ = 7.9 Hz, 1H, H-1), 4.46 (d, $^2J_{C\underline{H}H',CH\underline{H}'}$ = 16.1 Hz, 1H, CH<u>H</u>'C≡C), 4.43 (d, $^2J_{C\underline{H}H',CH\underline{H}'}$ = 16.1 Hz, 1H, C<u>H</u>H'C≡C), 4.04 (dd, $^2J_{5ax,5eq}$ = 12.0 Hz, $^3J_{4,5eq}$ = 5.4 Hz, 1H, H-5$_{eq}$), 3.65-3.58 (m, 1H, H-4), 3.41 (dd, $^3J_{2,3}$ 10.1 Hz, $^3J_{1,2}$ = 7.9 Hz, 1H, H-2), 3.22 (dd, $^2J_{5ax,5eq}$ = 12.0 Hz, $^3J_{4,5ax}$ = 10.8 Hz, 1H, H-5$_{ax}$), 2.177 (s, 3H, $C\underline{H}3_{\text{OAc at C-3}}$), 1.09-1.06 (m, 21H, TIPS) ppm.

^{13}C NMR (151 MHz, $CDCl_3$, 298 K): δ = 169.8 ($C{=}O_{\text{OAc at C-3}}$), 100.9 ($CH_2\underline{C}{\equiv}C$), 100.0 (C-1), 89.9 ($CH_2C{\equiv}\underline{C}$), 73.1 (C-3), 63.9 (C-5), 63.7 (C-2), 59.5 (C-4), 57.1 ($\underline{C}H_2C{\equiv}C$), 20.9 (OAc), 18.7 ($TIPS_{CH3}$), 11.2 ($TIPS_{CH}$) ppm.

1.7 3-(Triisopropylsilyl)propargyl 2,4-diazido-2,4-dideoxy-D-xylopyranoside (3)

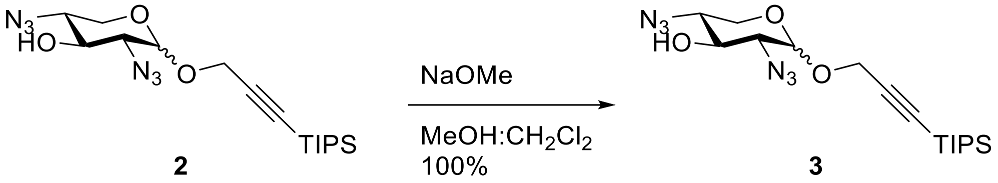

The protected xyloside **2** (348 mg, 798 µmol) was dissolved in a mixture of methanol:dichloromethane (1:1, 15.2 mL) and sodium methoxide (5.4 M in methanol, 30 µL, 162 µmol) was added dropwise. The reaction mixture was stirred at room temperature for 5 h. It was neutralised by addition of Amberlite®-IRC 120 H^+ resin. The resin was filtered off and the filtrate was concentrated under reduced pressure. The residue was coevaporated with toluene (3 x 10 mL) followed by dichloromethane (3 x 10 mL) to obtain the product **3** as a colourless syrup (314 mg, 796 µmol, 100%).

$\boldsymbol{R}_{\mathbf{F}}$ (cyclohexane:ethyl acetate, 3:1) = 0.55.

$[\alpha]_D^{20}$ = 56.976 (c = 0.17, $CHCl_3$).

IR (ATR): $\tilde{\nu}$ = 3442, 2943, 2866, 2106, 1254, 670 cm^{-1}.

ESI-HRMS: *m/z* = 395.22206 $[C_{17}H_{30}N_6O_3Si+H]^+$ (calculated *m/z* = 395.22214 $[C_{17}H_{30}N_6O_3Si+H]^+$).

3α 38%

^{1}H NMR (600 MHz, $CDCl_3$, 298 K): δ = 5.20 (d, $^3J_{1,2}$ = 3.5 Hz, 1H, H-1), 4.34-4.29 (m, 2H, (C$\underline{H}_2$C≡C), 3.99-3.94 (m, 1H, H-3), 3.75-3.69 (m, 1H, H-5a), 3.58-3.52 (m, 2H, H-4, H-5b), 3.27 (dd, $^3J_{2,3}$ 10.1 Hz, $^3J_{1,2}$ = 3.5 Hz, 1H, H-2), 2.60 (d, $^3J_{3,OH}$ = 3.7 Hz, 1H, O$\underline{H}$), 1.12-1.05 (m, 21H, TIPS) ppm.

^{13}C NMR (151 MHz, $CDCl_3$, 298 K): δ = 101.3 ($CH_2\underline{C}$≡C), 96.0 (C-1), 89.3 (CH_2C≡$\underline{C}$), 71.1 (C-3), 63.4 (C-2), 62.0 (C-4), 60.3 (C-5), 55.9 ($\underline{C}H_2$C≡C), 18.7 ($TIPS_{CH3}$), 11.2 ($TIPS_{CH}$) ppm.

3β 62%

^{1}H NMR (600 MHz, $CDCl_3$, 298 K): δ = 4.59 (d, $^3J_{1,2}$ = 7.7 Hz, 1H, H-1), 4.45 (d, $^2J_{C\underline{H}H',CH\underline{H}'}$ = 16.1 Hz, 1H, CH$\underline{H}$'C≡C), 4.42 (d, $^2J_{C\underline{H}H',CH\underline{H}'}$ = 16.1 Hz, 1H, C$\underline{H}$H'C≡C), 4.00 (dd, $^2J_{5ax,5eq}$ = 11.9 Hz, $^3J_{4,5eq}$ = 5.4 Hz, 1H, H-5_{eq}), 3.58-3.52 (m, 1H, H-4), 3.39 (td, $^3J_{2,3}$ = $^3J_{3,4}$ = 9.4 Hz, $^3J_{3,OH}$ = 3.2 Hz), 3.33 (dd, $^3J_{2,3}$ = 9.7 Hz, $^3J_{1,2}$ = 7.8 Hz, 1H, H-2), 3.14 (dd, $^2J_{5ax,5eq}$ = 12.0 Hz, $^3J_{4,5ax}$ = 10.8 Hz, 1H, H-5_{ax}), 2.63 (d, $^3J_{3,OH}$ = 3.2 Hz, 1H, O$\underline{H}$), 1.12-1.05 (m, 21H, TIPS) ppm.

^{13}C NMR (151 MHz, $CDCl_3$, 298 K): δ = 101.2 ($CH_2\underline{C}$≡C), 99.9 (C-1), 89.6 (CH_2C≡$\underline{C}$), 74.4 (C-3), 65.9 (C-2), 64.1 (C-5), 60.9 (C-4), 57.0 ($\underline{C}H_2$C≡C), 18.7 ($TIPS_{CH3}$), 11.2 ($TIPS_{CH}$) ppm.

1.8 3-(Triisopropylsilyl)propargyl 2,4-dideoxy-2,4-*N*-Boc-α-D-xylopyranoside (4) and 3-(Triisopropylsilyl)propargyl 2,4-dideoxy-2,4-*N*-Boc-β-D-xylopyranoside (5)

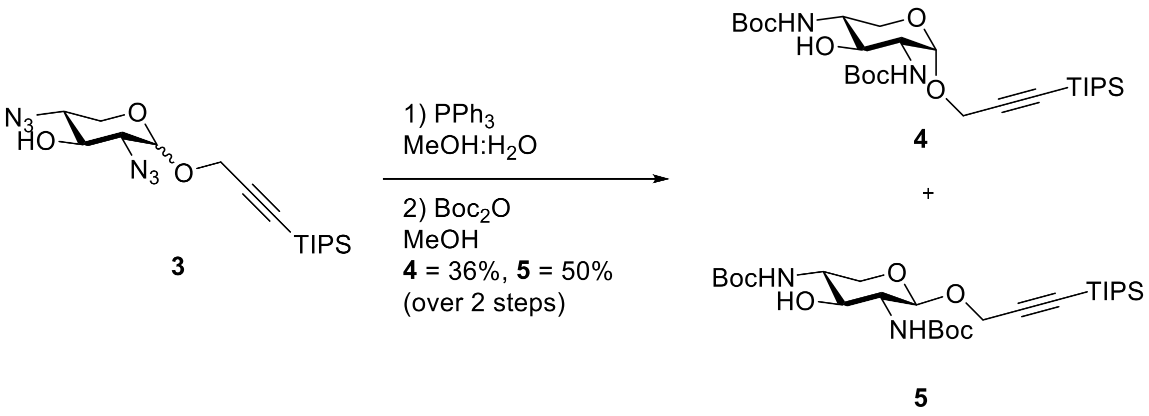

The di-azido xyloside **3** (381 mg, 966 µmol) was dissolved in MeOH:THF:H_2O (15:12:2, 7.6 mL), stirred and PPh_3 (537.0 mg, 2.05 mmol) was added. The mixture was stirred for 7.5 h at room temperature. Afterwards the solvents were removed under reduced pressure. The residue was dissolved in methanol (25 mL) and stirred at room temperature. A solution of Boc_2O (435 mg, 1.99 mmol) dissolved in methanol (15 mL) was added and the mixture was stirred at room temperature for 10 hwas dissolved in dichloromethane and loaded onto Celite®. The crude product was purified on silica gel (cyclohexane:ethyl acetate, 90:10 → 70:30) to obtain the anomers **4** as a colourless powder (189 mg, 349 µmol, 36%) and **5** as a colourless solid (260 mg, 480 µmol, 50%).

3-(Triisopropylsilyl)propargyl 2,4-dideoxy-2,4-*N*-Boc-α-D-xylopyranoside (**4**)

$\boldsymbol{R}_F$ (cyclohexane:ethyl acetate, 3:2) = 0.51.

$[\alpha]_D^{20}$ = 37.142 (c = 0.18, $CHCl_3$).

IR (ATR): $\tilde{\nu}$ = 3317, 2943, 2866, 1682, 1531, 1172, 1036, 678 cm^{-1}.

ESI-HRMS: *m/z* = 565.32763 $[C_{27}H_{50}N_2O_7Si+Na]^+$ (calculated *m/z* = 565.32795 $[C_{27}H_{50}N_2O_7Si+Na]^+$).

^{1}H NMR (600 MHz, $CDCl_3$, 298 K): δ = 5.04 (d, $^3J_{1,2}$ = 3.8 Hz, 1H, H-1), 5.02-4.92 (m, 1H, N$\underline{H}_{C\text{-}2}$), 4.66-4.53 (m, 1H, N$\underline{H}_{C\text{-}4}$), 4.29-4.19 (m, 2H, C$\underline{H}_2$C≡C), 3.95-3.82 (m, 1H, H-5_{eq}), 3.82-3.73 (m, 1H, H-2), 3.68-3.58 (m, 1H, H-4), 3.57-3.50 (m, 1H, H-3), 3.40 (t, $^2J_{5ax,5eq}$ = $^3J_{4,5ax}$ = 10.9 Hz, 1H, H-5_{ax}), 3.46-3.25 (m, 1H, O$\underline{H}$), 1.48-1.39 (m, 18H, 2 x Boc (C(C$\underline{H}_3$)$_3$)), 1.11-1.02 (m, 21H, TIPS) ppm.

^{13}C NMR (151 MHz, $CDCl_3$, 298 K): δ = 157.4 (C=$O_{Boc\ at\ C\text{-}2}$), 156.1, 102.0 ($\underline{C}$≡C-Si), 96.0 (C-1), 88.8 (C≡$\underline{C}$-Si), 80.6 ($\underline{C}$(CH_3)$_3$), 72.9 (C-3), 61.3 (C-5), 55.4 (C-2), 55.3 ($\underline{C}H_2$C≡C), 53.9 (C-4), 28.5 (C($\underline{C}H_3$)$_3$), 28.4 (C($\underline{C}H_3$)$_3$), 18.7 (TIPS), 11.2 (TIPS) ppm.

3-(Triisopropylsilyl)propargyl 2,4-dideoxy-2,4-*N*-Boc-β-D-xylopyranoside (**5**)

***R*F** (cyclohexane:ethyl acetate, 3:2) = 0.37.

$[\alpha]_D^{20}$ = -58.992 (c = 0.14, $CHCl_3$).

IR (ATR): $\tilde{\nu}$ = 3302, 2943, 2866, 1686, 1536, 1172, 1049, 678 cm^{-1}.

ESI-HRMS: *m/z* = 543.34593 $[C_{27}H_{50}N_2O_7Si+H]^+$ (calculated *m/z* = 543.34600 $[C_{27}H_{50}N_2O_7Si+H]^+$).

^{1}H NMR (600 MHz, $CDCl_3$, 298 K): δ = 5.17-4.98 (m, 1H, NH or OH), 4.90-4.79 (m, 1H, NH or OH), 4.77-4.65 (m, 1H, NH or OH), 4.61 (d, $^3J_{1,2}$ = 7.1 Hz, 1H, H-1), 4.44-4.35 (m, 2H, $CH_2C≡C$), 4.31-4.20 (m, 1H, H-5_{eq}), 3.64-3.51 (m, 2H, H-3, H-4), 3.44-3.35 (m, 1H, H-2), 3.19 (br dd, $^2J_{5ax,5eq}$ = 11.1 Hz, $^3J_{4,5ax}$ = 9.3 Hz 1H, H-5ax), 1.49-1.38 (m, 18H, 2 x $C(CH_3)_3$), 1.12-1.04 (m, 21H, TIPS) ppm.

^{13}C NMR (151 MHz, $CDCl_3$, 298 K): δ = 156.0, 101.9 (C≡C-Si), 98.6 (C-1), 89.3 (C≡C-Si), 81.0 ($C(CH_3)_3$), 73.5 (C-3), 64.3 (C-5), 57.4 (C-2), 56.7 ($CH_2C≡C$), 53.1 (C-4), 28.5 $C(CH_3)_3$, 28.4 $C(CH_3)_3$, 18.7 (TIPS), 11.2 (TIPS) ppm.

1.9 3-(Triisopropylsilyl)propargyl 2,4-dideoxy-2,4-*N*-Boc-3-*O*-propargyl-α-D-xylopyranoside (6)

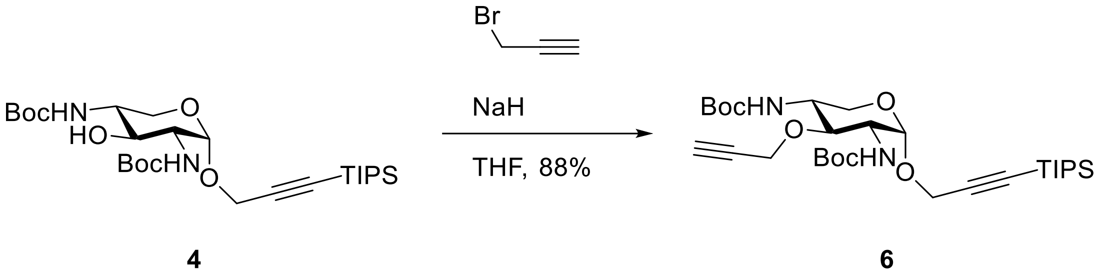

3-(Triisopropylsilyl)propargyl 2,4-dideoxy-2,4-*N*-Boc-α-D-xylopyranoside (**4**) (173 mg, 318 µmol) was dissolved in dry tetrahydrofuran (6.0 mL) under a nitrogen atmosphere. At 0 °C sodium hydride (19.0 mg, 475 µmol, 60% dispersion in mineral oil) was added and it was stirred at 0 °C for 10 min. Propargyl bromide (100 µL, 920 µmol, 9.2 M in toluene) was added dropwise. The reaction mixture was stirred for 10 min at 0 °C. The ice bath was removed and it was further stirred at room temperature for 76 min. Afterwards dichloromethane (70 mL) and water (30 mL) were added. The layers were separated and the aqueous layer was extracted with dichloromethane (15 mL). The combined organic layers were dried over $MgSO_4$, it was filtered and concentrated. The crude product was purified on silica gel (cyclohexane:ethyl acetate, 95:5 → 75:25) to obtain the title compound **6** as a colourless foam (162 mg, 279 µmol, 88%).

***R*F** (cyclohexane:ethyl acetate, 3:2) = 0.79.

$[\alpha]_D^{20}$ = 40.769 (c = 0.13, $CHCl_3$).

IR (ATR): $\tilde{\nu}$ = 3354, 2944, 2870, 1770, 1759, 1694, 1246, 1036, 666 cm^{-1}.

ESI-HRMS: *m/z* = 598.38743 $[C_{30}H_{52}N_2O_7Si+NH_4]^+$ (calculated *m/z* = 598.38820 $[C_{30}H_{52}N_2O_7Si+NH_4]^+$).

^{1}H NMR (600 MHz, $CDCl_3$, 298 K): δ = 5.00 (d, $^3J_{1,2}$ = 3.5 Hz, 1H, H-1), 4.87-4.48 (m, 2H, 2 x N<u>H</u>), 4.40-4.23 (m, 4H, 2 x C<u>H</u>$_2$C≡C), 3.98-3.74 (m, 2H, H-2, H-5$_{eq}$), 3.71-3.53 (m, 2H, H-4, H-3), 3.47 (t, $^2J_{5ax,5eq}$ = $^3J_{4,5ax}$ = 10.7 Hz, 1H, H-5$_{ax}$), 2.49 (s, 1H, C≡C<u>H</u>), 1.53-1.33 (m, 18H, 2 x C(C<u>H</u>$_3$)$_3$), 1.17-0.95 (m, 21H, TIPS) ppm.

^{13}C NMR (151 MHz, $CDCl_3$, 298 K): δ = 155.6 (C=O$_{Boc}$), 155.4 (C=O$_{Boc}$), 102.0 (<u>C</u>≡C-Si), 96.7 (C-1), 88.7 (C≡<u>C</u>-Si), 80.6 (CH$_2$<u>C</u>≡CH), 79.9 (2 x O-<u>C</u>(CH$_3$)$_3$), 77.2 (C-3), 75.1 (CH$_2$C≡<u>C</u>), 62.0 (C-5), 58.5 (<u>C</u>H$_2$C≡CH), 55.2 (<u>C</u>H$_2$C≡C-Si), 53.8 (C-2), 51.5 (C-4), 28.5 (Boc), 18.7 (TIPS), 11.2 (TIPS) ppm.

1.10 3-(Triisopropylsilyl)propargyl 2,4-dideoxy-2,4-*N*-Boc-3-*O*-propargyl-β-D-xylopyranoside (7)

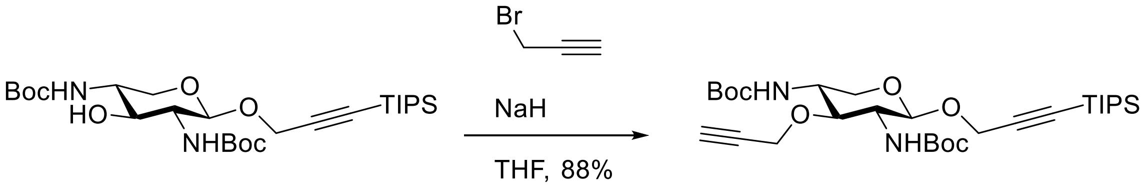

5 **7**

3-(Triisopropylsilyl)propargyl 2,4-dideoxy-2,4-*N*-Boc-β-D-xylopyranoside (**5**) (135 mg, 248 µmol) was dissolved in dry tetrahydrofuran (8.7 mL) under a nitrogen atmosphere. At 0 °C sodium hydride (17.8 mg, 445 µmol, 60% dispersion in mineral oil) was added and it was further stirred at 0 °C for 15 min. Propargyl bromide (48.0 µL, 442 µmol, 9.2 M in toluene) was added dropwise. The reaction mixture was stirred at room temperature for 90 min. Afterwards dichloromethane (50 mL) and water (15 mL) were added. The layers were separated and the aqueous layer was extracted with dichloromethane (25 mL). The combined organic layers were dried over $MgSO_4$, it was filtered and concentrated. The crude product was purified on silica gel (cyclohexane:ethyl acetate, 95:5 → 70:30) to obtain the title compound **7** as a colourless solid (126 mg, 218 µmol, 88%).

***R*$_F$** (cyclohexane:ethyl acetate, 3:2) = 0.74.

$[\alpha]_D^{20}$ = -53.012 (c = 0.17, $CHCl_3$).

IR (ATR): $\tilde{\nu}$ = 3289, 2941, 2865, 1685, 1537, 1173, 1066, 655 cm^{-1}.

ESI-HRMS: *m/z* = 581.36100 $[C_{30}H_{52}N_2O_7Si+H]^+$ (calculated *m/z* = 581.36166 $[C_{30}H_{52}N_2O_7Si+H]^+$).

^{1}H NMR (600 MHz, $CDCl_3$, 298 K): δ = 4.99-4.66 (m, 3H, H-1, 2 x N<u>H</u>), 4.40-4.25 (m, 4H, 2 x C<u>H</u>$_2$C≡C), 4.18 (br dd, $^2J_{5ax,5eq}$ = 11.5 Hz, $^3J_{4,5eq}$ = 3.3 Hz, 1H, H-5$_{eq}$), 3.89-3.75 (m, 1H, H-3), 3.65-3.56 (m, 1H, H-4), 3.53-3.44 (m, 1H, H-2), 3.27 (br dd, $^2J_{5ax,5eq}$ = 11.2 Hz, $^3J_{4,5ax}$ = 7.6 Hz, 1H, H-5$_{ax}$), 2.52-2.44 (m, 1H, C≡C<u>H</u>), 1.45 (s, 9H, C(C<u>H</u>$_3$)$_3$), 1.44 (s, 9H, C(C<u>H</u>$_3$)$_3$), 1.15-0.94 (m, 21H, TIPS) ppm.

^{13}C NMR (151 MHz, $CDCl_3$, 298 K): δ = 155.5 ($C{=}O_{Boc}$), 155.2 ($C{=}O_{Boc}$), 102.3 (C≡C-Si), 98.5 (C-1), 88.3 (C≡C-Si), 80.2 ($CH_2C{\equiv}CH$, 2 x O-$C(CH_3)_3$), 76.4 (C-3), 75.1 ($CH_2C{\equiv}C$), 62.5 (C-5), 58.3 ($CH_2C{\equiv}C$), 56.3 ($CH_2C{\equiv}C$), 54.3 (C-2), 50.6 (C-4), 28.5 (Boc), 28.4 (Boc), 18.7 (TIPS), 11.3 (TIPS) ppm.

1.11 3-(Triisopropylsilyl)propargyl 2,4-*N*-carbonyl-2,4-diamino-2,4-dideoxy-3-*O*-propargyl-α-D-xylopyranoside (8)

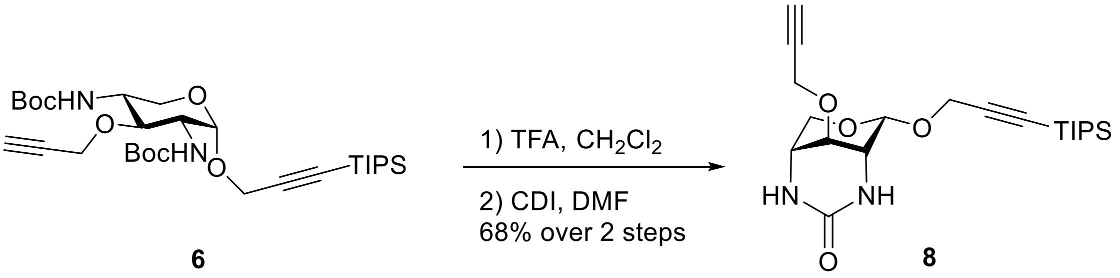

3-(Triisopropylsilyl)propargyl 2,4-dideoxy-2,4-*N*-Boc-3-*O*-propargyl-α-D-xylopyranoside (**6**) (43.1 mg, 74.2 µmol) was dissolved in dichloromethane (5.0 mL) and trifluoroacetic acid (1.0 mL) was added dropwise while stirring at room temperature. The reaction mixture was stirred for 1 h and then all solvents were removed under reduced pressure. The residue was coevaporated with toluene (3 x 5.0 mL) and dichloromethane (5.0 mL) to obtain a colourless solid. Carbonyldiimidazole (16.7 mg, 103 µmol) was added and the solids were suspended in dry DMF (730 µL). The reaction mixture was stirred at room temperature for 160 min and then diluted with ethyl acetate (30 mL). The organic phase was washed brine (3 x 10 mL), separated, dried over $MgSO_4$, it was filtered and concentrated. The residue was dissolved in dichloromethane and loaded onto Celite®. The crude product was purified on silica gel (CH_2Cl_2:MeOH, 100:0 → 90:10) to obtain the title compound **8** as a colourless solid (20.6 mg, 50.7 µmol, 68%).

$\boldsymbol{R}_F$ (dichloromethane:methanol, 9:1) = 0.47.

$[\boldsymbol{\alpha}]_{\mathbf{D}}^{\mathbf{20}}$ = 22.131 (c = 0.12, $CHCl_3$).

IR (ATR): $\tilde{\nu}$ = 3219, 3081, 2944, 2866, 2170, 1686, 1034, 665 cm^{-1}.

ESI-HRMS: *m/z* = 429.21767 $[C_{21}H_{34}N_2O_4Si{+}Na]^+$ (calculated *m/z* = 429.21801 $[C_{21}H_{34}N_2O_4Si{+}Na]^+$).

^{1}H NMR (600 MHz, $CDCl_3$, 298 K): δ = 5.14-5.13 (m, 1H, H-1), 5.02-4.97 (m, 1H, $NH_{at\ C\text{-}4}$), 4.89-4.84 (m, 1H, $NH_{at\ C\text{-}2}$), 4.43 (d, $^2J_{CH2,CH2}$ = 16.1 Hz, 1H, $CHH'C{\equiv}C_{at\ C\text{-}1}$), 4.35 (d, $^2J_{CH2,CH2}$ = 16.1 Hz, 1H, $CH'HC{\equiv}C_{at\ C\text{-}1}$), 4.30-4.22 (m, 3H, $CH_2C{\equiv}C_{at\ C\text{-}3}$, H-3), 4.03 (d, $^2J_{5a,5b}$ = 11.8 Hz, 1H, H-5a), 3.78-3.73 (m, 1H, H-5b), 3.47-3.44 (m, 1H, H-2), 3.39-3.35 (m, 1H, H-4), 2.49 (t, $^4J_{CH2,C\equiv CH}$ = 2.4 Hz, 1H, C≡CH), 1.11-1.04 (m, 21H, TIPS) ppm.

^{13}C NMR (151 MHz, $CDCl_3$, 298 K): δ = 157.8 (C=O), 102.2 (C≡C-Si), 95.0 (C-1), 88.8 (C≡C-Si), 78.8 ($CH_2C{\equiv}CH$), 75.9 ($CH_2C{\equiv}CH$), 71.5 (C-3), 66.6 (C-5), 57.3 ($CH_2C{\equiv}CH$), 55.9 ($CH_2C{\equiv}C$-Si), 51.0 (C-2), 47.7 (C-4), 18.7 (TIPS), 11.3 (TIPS) ppm.

1.12 3-(Triisopropylsilyl)propargyl 2,4-*N*-carbonyl-2,4-diamino-2,4-dideoxy-3-*O*-propargyl-β-D-xylopyranoside (9)

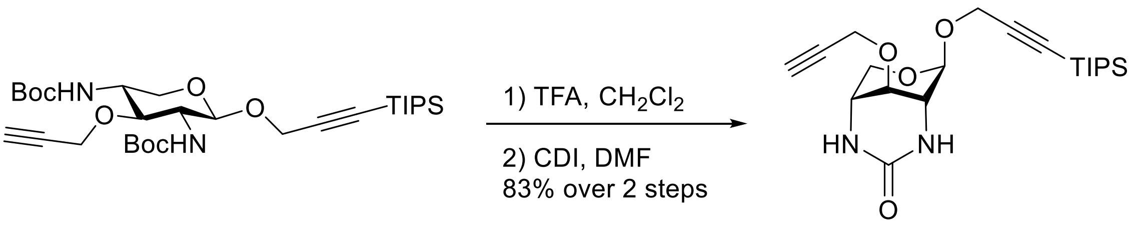

7 **9**

3-(Triisopropylsilyl)propargyl 2,4-dideoxy-2,4-*N*-Boc-3-*O*-propargyl-β-D-xylopyranoside (**7**) (46.5 mg, 80.1 µmol) was dissolved in dichloromethane (5.0 mL) and trifluoroacetic acid (1.0 mL) was added dropwise while stirring at room temperature. The reaction mixture was stirred for 1 h and then all solvents were removed under reduced pressure. The residue was coevaporated with toluene (3 x 5.0 mL) and dichloromethane (5.0 mL) to obtain a colourless syrup. Carbonyldiimidazole (22.1 mg, 136 µmol) was added and the solids were suspended in dry DMF (730 µL). The reaction mixture was stirred at room temperature for 5 h 15 min and then diluted with ethyl acetate (50 mL). The organic phase was washed brine (3 x 10 mL), separated, dried over $MgSO_4$, it was filtered and concentrated. The crude product was purified on silica gel (dichloromethane:MeOH, 100:0 → 90:10) to obtain the title compound **9** as a colourless solid (26.9 mg, 66.2 µmol, 83%).

$\boldsymbol{R}_\mathrm{F}$ (dichloromethane:methanol, 9:1) = 0.47.

$[\boldsymbol{\alpha}]_\mathbf{D}^{\mathbf{20}}$ = -199.113 (c = 0.11, $CHCl_3$).

IR (ATR): $\tilde{\nu}$ = 3234, 2942, 2865, 2178, 1680, 1456, 1128, 1017, 667 cm^{-1}.

ESI-HRMS: *m/z* = 445.19184 $[C_{21}H_{34}N_2O_4Si+K]^+$ (calculated *m/z* = 445.19194 $[C_{21}H_{34}N_2O_4Si+K]^+$).

^{1}H NMR (600 MHz, $CDCl_3$, 298 K): δ = 5.49-5.44 (m, 1H, N$\underline{H}_{\text{at C-4}}$), 5.29-5.25 (m, 1H, N$\underline{H}_{\text{at C-2}}$), 4.94-4.92 (m, 1H, H-1), 4.36 (dd, $^2J_{CH2,CH2}$ = 16.2 Hz, $^4J_{CH2,C≡CH}$ = 2.4 Hz, 1H, CH$\underline{H}$'C≡$C_{\text{at C-3}}$), 4.33-4.28 (m, 2H, C$\underline{H}_2$C≡$C_{\text{at C-1}}$), 4.29 (dd, $^2J_{CH2,CH2}$ = 16.2 Hz, $^4J_{CH2,C≡CH}$ = 2.4 Hz, 1H, CH'$\underline{H}$C≡$C_{\text{at C-3}}$), 4.24-4.17 (m, 2H, H-3, H-5a), 3.51-3.48 (m, 1H, H-2), 3.44-3.39 (m, 2H, H-4, H-5b), 2.48 (t, $^4J_{CH2,C≡CH}$ = 2.4 Hz, 1H, C≡C$\underline{H}$), 1.10-1.02 (m, 21H, TIPS) ppm.

^{13}C NMR (151 MHz, $CDCl_3$, 298 K): δ = 157.3 (C=O), 102.7 ($\underline{C}$≡C-Si), 98.5 (C-1), 88.1 (C≡$\underline{C}$-Si), 78.8 ($CH_2\underline{C}$≡CH), 75.8 (CH_2C≡$\underline{C}$H), 68.6 (C-3), 60.7 (C-5), 56.3 ($\underline{C}H_2$C≡CH), 55.8 ($\underline{C}H_2$C≡C-Si), 48.2 (C-4), 47.6 (C-2), 18.7 (TIPS), 11.3 (TIPS) ppm.

1.13 Propargyl 2,4-*N*-carbonyl-2,4-diamino-2,4-dideoxy-3-*O*-propargyl-β-D-xylopyranoside (10)

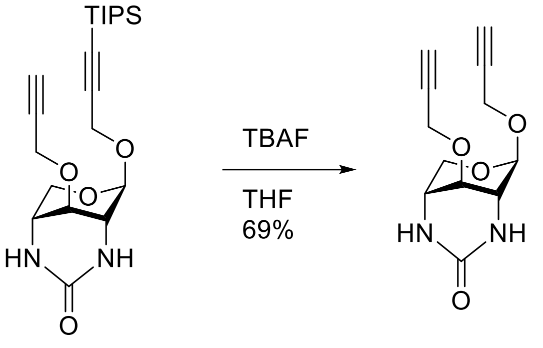

The protected xyloside **9** (53.7 mg, 132 µmol) was dissolved in tetrahydrofuran (9.0 mL) and cooled to 0 °C. Tetrabutylammonium fluoride (1 M in tetrahydrofuran, 140 µL, 140 µmol) was added dropwise. The mixture was further stirred at 0 °C for 36 min. The reaction mixture was diluted with dichloromethane (50 mL) and washed with brine (25 mL). The aqueous layer was separated and extracted with dichloromethane (15 mL). The combined organic layers were dried over $MgSO_4$, it was filtered and concentrated. The crude product was twice purified on silica gel via automated flash chromatography (CH_2Cl_2:MeOH, 100:0 → 90:10) to obtain the title compound **10** as a colourless foam (22.9 mg, 91.5 µmol, 69%).

$\boldsymbol{R}_{\mathbf{F}}$ (dichloromethane:methanol, 9:1) = 0.41.

$[\alpha]_D^{20}$ = -22.472 (c = 0.11, $CHCl_3$).

IR (ATR): $\tilde{\nu}$ = 2927, 1745, 1674, 1511, 1368, 1214, 1034, 819 cm^{-1}.

ESI-HRMS: *m/z* = 289.05864 $[C_{12}H_{14}N_2O_4+K]^+$ (calculated *m/z* = 289.05852 $[C_{12}H_{14}N_2O_4+K]^+$).

^{1}H NMR (600 MHz, CD_3CN, 298 K): δ = 5.36-5.29 (m, 1H, N<u>H</u>$_{at\ C-4}$), 5.29-5.22 (m, 1H, N<u>H</u>$_{at\ C-2}$), 4.75 (s, 1H, H-1), 4.29 (dd, $^2J_{CH2,CH2}$ = 16.0 Hz, $^4J_{CH2,C≡CH}$ = 2.5 Hz, 1H, C<u>H</u>H'C≡C$_{at\ C-3}$), 4.25 (dd, $^2J_{CH2,CH2}$ = 15.9 Hz, $^4J_{CH2,C≡CH}$ = 2.4 Hz, 1H, C<u>H</u>H'C≡C$_{at\ C-1}$), 4.23 (dd, $^2J_{CH2,CH2}$ = 15.8 Hz, $^4J_{CH2,C≡CH}$ = 2.6 Hz, 1H, CH<u>H'</u>C≡C$_{at\ C-1}$), 4.21 (dd, $^2J_{CH2,CH2}$ = 16.0 Hz, $^4J_{CH2,C≡CH}$ = 2.4 Hz, 1H, CH<u>H'</u>C≡C$_{at\ C-3}$), 4.06-4.01 (m, 2H, H-5a, H-3), 3.40-3.37 (m, 1H, H-2), 3.34-3.28 (m, 2H, H-5b, H-4), 2.75 (t, $^4J_{CH2,C≡CH}$ = 2.4 Hz, 1H, C≡C<u>H</u>$_{at\ C-3}$), 2.71 (t, $^4J_{CH2,C≡CH}$ = 2.4 Hz, 1H, C≡C<u>H</u>$_{at\ C-1}$) ppm.

^{13}C NMR (151 MHz, CD_3CN, 298 K): δ = 157.5 (C=O), 100.1 (C-1), 80.39 (CH_2<u>C</u>≡CH), 80.36 (CH_2<u>C</u>≡CH$_{at\ C-1}$), 76.4 (CH_2C≡<u>C</u>H$_{at\ C-3}$), 75.9 (CH_2C≡<u>C</u>H$_{at\ C-1}$), 70.5 (C-3), 61.4 (C-5), 57.1 (<u>C</u>H_2C≡CH$_{at\ C-3}$), 55.4 (<u>C</u>H_2C≡CH$_{at\ C-1}$), 48.5 (C-4), 48.3 (C-2), 41.4 (DMSO) ppm.

1.14 4-(2-Azidoethyl)phenyl 2,3,4,6-tetra-*O*-benzoyl-β-D-glucopyranoside (15)

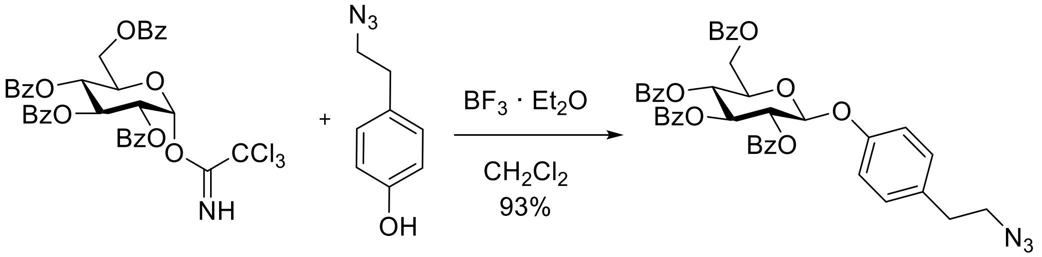

13 **11** **15**

2,3,4,6-Tetra-*O*-benzoyl-α-D-glucopyranosyl trichloroacetimidate (**13**) (1.06 g, 1.44 mmol), 4-(2-azidoethyl)phenol (**11**) [5,6] (194 mg, 1.19 mmol) and freshly activated powdered molecular sieves (4Å, 600 mg) were suspended in dry dichloromethane (7.0 mL) under a nitrogen atmosphere. The reaction mixture was stirred at room temperature for 15 min. At 0 °C boron trifluoride diethyl etherate (15.0 µL, 118 µmol) was added dropwise and further stirred at 0 °C for 30 min. The reaction mixture was quenched with Et_3N (100 µL), filtered over Celite® and the organic layer diluted with dichloromethane and loaded onto Celite®. The crude product was purified on silica gel (cyclohexane:ethyl acetate, 90:10 → 70:30) to obtain the title compound as a colourless foam **15** (823 mg, 1.11 mmol, 93%).

$\boldsymbol{R}_F$ (cyclohexane:ethyl acetate, 3:2) = 0.70.

$[\alpha]_D^{20}$ = +27.562 (c = 0.44, $CHCl_3$).

IR (ATR): $\tilde{\nu}$ = 2954, 2097, 1723, 1260, 1091, 1067, 707 cm^{-1}.

ESI-HRMS: *m/z* = 759.26527 $[C_{42}H_{35}N_3O_{10}+NH_4]^+$ (calculated *m/z* = 759.26607 $[C_{42}H_{35}N_3O_{10}+NH_4]^+$).

^{1}H NMR (600 MHz, $CDCl_3$, 298 K): δ = 8.06-8.02 (m, 2H, OBz_{ortho}), 7.97-7.95 (m, 2H, OBz_{ortho}), 7.95-7.92 (m, 2H, OBz_{ortho}), 7.88-7.84 (m, 2H, OBz_{ortho}), 7.61-7.56 (m, 1H, OBz_{para}), 7.54-7.50 (m, 2H, 2 x OBz_{para}), 7.47-7.41 (m, 3H, OBz_{para}, OBz_{meta}), 7.40-7.34 (m, 4H, 2 x OBz_{meta}), 7.33-7.28 (m, 2H, OBz_{meta}), 7.01-7.63 (m, 4H, $C\underline{H}_{Phenyl}$), 5.98 (t, $^3J_{2,3}$ = $^3J_{3,4}$ = 9.5 Hz, 1H, H-3), 5.79 (dd, $^3J_{2,3}$ = 9.6 Hz, $^3J_{1,2}$ = 7.8 Hz, 1H, H-2), 5.71(dd, $^3J_{4,5}$ = 9.9 Hz, $^3J_{3,4}$ = 9.4 Hz, 1H, H-4), 5.37 (d, $^3J_{1,2}$ = 7.8 Hz, 1H, H-1), 4.67 (dd, $^2J_{6a,6b}$ = 12.1 Hz, $^3J_{5,6}$ = 3.0 Hz, 1H, H-6a), 4.54 (dd, $^2J_{6a,6b}$ = 12.1 Hz, $^3J_{5,6}$ = 6.6 Hz, 1H, H-6b), 4.32 (ddd, $^3J_{4,5}$ = 9.7 Hz, $^3J_{5,6b}$ = 6.6 Hz, $^3J_{5,6a}$ = 3.1 Hz, 1H, H-5), 3.41 (t, $^3J_{CH2,CH2}$ = 7.2 Hz, 2H, CH_2-$C\underline{H}_2$-N_3), 2.78 (t, $^3J_{CH2,CH2}$ = 7.2 Hz, 2H, $C\underline{H}_2$-CH_2-N_3) ppm.

^{13}C NMR (151 MHz, $CDCl_3$, 298 K): δ = 166.2 ($C{=}O_{OBz\ at\ C\text{-}6}$), 165.9 ($C{=}O_{OBz\ at\ C\text{-}3}$), 165.4 ($C{=}O_{OBz\ at\ C\text{-}4}$), 165.2 ($C{=}O_{OBz\ at\ C\text{-}4}$), 155.9 ($\underline{C}_{ipso\text{-}Phenol}$), 133.7 ($OBz_{para}$), 133.5 (2 x OBz_{para}), 133.4 (OBz_{para}), 133.1 ($\underline{C}_{p\text{-}Phenol}$), 130.0 ($OBz_{ortho}$), 129.97 ($OBz_{ortho}$), 129.94 (2 x OBz_{ortho}), 129.90 ($\underline{C}_{m\text{-}Phenol}$), 129.7 ($OBz_{ipso}$), 129.2 ($OBz_{ipso}$), 128.9 ($OBz_{ipso}$), 128.8 ($OBz_{ipso}$), 128.62 ($OBz_{meta}$), 128.55 (2 x OBz_{meta}), 128.5 (OBz_{meta}), 117.6 ($\underline{C}_{o\text{-}Phenol}$), 99.9 (C-1), 72.9 (C-3), 72.7 (C-5), 71.8 (C-2), 69.8 (C-4), 63.4 (C-6), 52.6 ($\underline{C}H_2$-N), 34.7 ($\underline{C}H_2$-CH_2-N) ppm.

1.15 Glycoconjugate 16

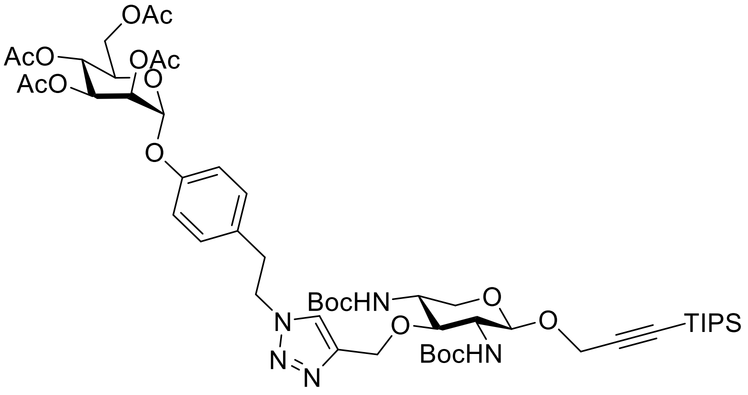

Under a nitrogen atmosphere, 4-(2-azidoethyl)phenyl 2,3,4,6-tetra-*O*-acetyl-α-D-mannopyranoside (**14**) [7] (340 mg, 690 µmol), propargyl xyloside **7** (306 mg, 526 µmol), sodium ascorbate (424 mg, 2.14 mmol) and anhydrous copper(II)sulfate (169 mg, 1.06 mmol) were stirred at room temperature. Degassed tetrahydrofuran (18.3 mL) was added followed by degassed water (4.6 mL). The mixture turned black which changed to orange after stirring for 4 min. After stirring for 220 min the mixture was diluted with dichloromethane (150 mL) and washed with EDTA disodium salt dihydrate solution (50 mL, 10 mM). The organic layer was separated and the aqueous layer was extracted with dichloromethane (25 mL). The combined organic layers were dried over $MgSO_4$, it was filtered and concentrated under reduced pressure to obtain a colourless syrup. The crude product was purified on silica gel (toluene:acetone 90:10 → 60:40) to obtain **16** as a colourless solid (511 mg, 476 µmol, 90%).

$\boldsymbol{R}_\mathrm{F}$ (cyclohexane:ethyl acetate, 3:7) = 0.68.

$[\boldsymbol{\alpha}]_\mathbf{D}^{\mathbf{20}}$ = +6.962 (c = 0.16, $CHCl_3$).

IR (**ATR**): $\tilde{\nu}$ = 3338, 2951, 2870, 1751, 1512, 1366, 1220, 1165, 1038 cm^{-1}.

ESI-HRMS: *m/z* = 1074.53070 $[C_{52}H_{79}N_5O_{17}Si+H]^+$ (calculated *m/z* = 1074.53130 $[C_{52}H_{79}N_5O_{17}Si+H]^+$).

^{1}H NMR (600 MHz, $CDCl_3$, 298 K): δ = 7.50 (s, 1H, C=C$\underline{H}_{Triazole}$), 7.11-7.05 (m, 2H, C$\underline{H}_{m\text{-Phenol}}$), 7.05-7.00 (m, 2H, C$\underline{H}_{o\text{-Phenol}}$), 5.55 (dd, $^3J_{3,4}$ = 9.9 Hz, $^3J_{2,3}$ = 3.7 Hz, 1H, H-3_{Man}), 5.49 (d, $^3J_{1,2}$ = 1.9 Hz, 1H, H-1_{Man}), 5.43 (dd, $^3J_{2,3}$ = 3.7 Hz, $^3J_{1,2}$ = 1.7 Hz, 1H, H-2_{Man}), 5.37 (t, $^3J_{3,4}$ = $^3J_{4,5}$ = 10.0 Hz, 1H, H-4_{Man}), 5.04-4.64 (m, 5H, N-C-C$\underline{H}_2$, H-1_{Xylose}, 2 x N$\underline{H}$), 4.55-4.47 (m, 2H, C$\underline{H}_2$-N), 4.36-4.32 (m, 2H, C$\underline{H}_2$C≡C), 4.30-4.26 (m, 1H, H-$6a_{Man}$), 4.20-4.13 (m, 1H, H-$5a_{Xylose}$), 4.12-4.05 (m, 2H, H-5_{Man}, H-$6b_{Man}$), 3.83-3.69 (m, 1H, H-3_{Xylose}), 3.69-3.55 (m, 2H, H-2_{Xylose}, H-4_{Xylose}), 3.33-3.22 (m, 1H, H-$5b_{Xylose}$), 3.20-3.14 (m, 2H, C$\underline{H}_2$-CH_2-N), 2.20 (s, 3H, OAc), 2.05 (s, 3H, OAc), 2.04 (s, 3H, OAc), 2.03 (s, 3H, OAc), 1.43 (s, 18H, Boc), 1.17-0.95 (m, 21H, TIPS) ppm.

^{13}C NMR (151 MHz, $CDCl_3$, 298 K): δ = 170.7 (C=$O_{OAc\ at\ C\text{-}6}$), 170.2 (C=$O_{OAc\ at\ C\text{-}2}$), 170.1 (C=$O_{OAc\ at\ C\text{-}3}$), 169.9 (C=$O_{OAc\ at\ C\text{-}4}$), 155.4 (C=O_{Boc}), 155.3 (C=O_{Boc}), 154.9 ($\underline{C}_{ipso\text{-Phenol}}$), 145.4 ($\underline{C}$=$CH_{Triazole}$), 131.8 ($\underline{C}_{p\text{-Phenol}}$), 130.0 ($\underline{C}_{m\text{-Phenol}}$), 123.0 (C=$\underline{C}H_{Triazole}$), 117.1 ($\underline{C}_{o\text{-Phenol}}$), 102.4 ($CH_2\underline{C}$≡C), 98.6 (C-$1_{Xylose}$), 96.1 (C-$1_{Man}$), 88.4 ($CH_2C$≡$\underline{C}$), 80.1 (2 x O-$\underline{C}(CH_3)_3$), 77.3 (C-$3_{Xylose}$), 69.6 (C-$3_{Man}$), 69.3 (C-$5_{Man}$), 69.0 (C-$3_{Man}$), 66.1 (C-$4_{Man}$), 64.4 (N-C-$\underline{C}H_2$), 62.3 (C-$5_{Xylose}$, C-$6_{Man}$), 56.2 ($\underline{C}H_2$C≡C), 54.0 (C-$2_{Xylose}$), 51.7 ($\underline{C}H_2$-N), 50.6 (C-$4_{Xylose}$), 36.0 ($\underline{C}H_2$-$CH_2$-N), 28.5 (Boc), 21.0 ($\underline{C}H3_{OAc}$), 20.8 (3 x $\underline{C}H3_{OAc}$), 18.7 (TIPS), 11.3 (TIPS) ppm.

1.16 Glycoconjugate 17

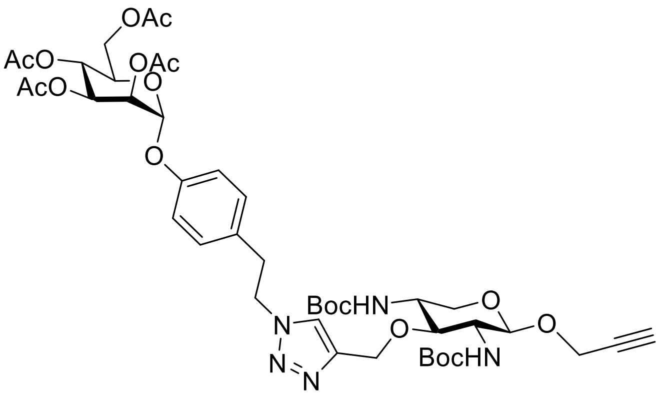

The TIPS-protected xyloside **16** (128 mg, 119 µmol) was dissolved in tetrahydrofuran (8.8 mL) and cooled to 0 °C. Tetrabutylammonium fluoride (1 M in tetrahydrofuran, 120 µL, 120 µmol) was added dropwise. The mixture was further stirred at 0 °C for 31 min. The reaction mixture was diluted with dichloromethane (100 mL) and washed with brine (25 mL). The aqueous layer was separated and extracted with dichloromethane (10 mL). The combined organic layers were dried over $MgSO_4$, it was filtered and concentrated. The residue was dissolved in dichloromethane and loaded onto Celite®. The crude product was purified on silica gel (cyclohexane:ethyl acetate, 70:30 → 30:70) to obtain the title compound **17** as a colourless solid (89.3 mg, 97.3 µmol, 82%).

***R*F** (cyclohexane:ethyl acetate, 3:7) = 0.44.

$[\alpha]_D^{20}$ = +8.064 (c = 0.19, $CHCl_3$).

IR (ATR): $\tilde{\nu}$ = 3344, 2976, 2923, 1748, 1709, 1512, 1366, 1218, 1036, 602 cm^{-1}.

ESI-HRMS: *m/z* = 918.39644 $[C_{43}H_{59}N_5O_{17}+H]^+$ (calculated *m/z* = 918.39787 $[C_{43}H_{59}N_5O_{17}+H]^+$).

^{1}H NMR (600 MHz, $CDCl_3$, 298 K): δ = 7.48 (s, 1H, C=$CH_{Triazole}$), 7.09-7.05 (m, 2H, $CH_{m\text{-Phenol}}$), 7.04-7.00 (m, 2H, $CH_{o\text{-Phenol}}$), 5.55 (dd, $^3J_{3,4}$ = 10.0 Hz, $^3J_{2,3}$ = 3.5 Hz, 1H, H-3_{Man}), 5.49 (d, $^3J_{1,2}$ = 1.9 Hz, 1H, H-1_{Man}), 5.42 (dd, $^3J_{2,3}$ = 3.5 Hz, $^3J_{1,2}$ = 1.9 Hz, 1H, H-2_{Man}), 5.37 (t, $^3J_{3,4}$ = $^3J_{4,5}$ = 10.2 Hz, 1H, H-4_{Man}), 5.00-4.68 (m, 5H, N-C-CH_2, H-1_{Xylose}, 2 x NH), 4.52 (t, $^3J_{CH2,CH2}$ = 7.6 Hz, 2H, CH_2-N), 4.35-4.25 (m, 3H, CH_2C≡C, H-$6a_{Man}$), 4.16-4.05 (m, 3H, H-$5a_{Xylose}$, H-5_{Man}, H-$6b_{Man}$), 3.97-3.80 (m, 1H, H-3_{Xylose}), 3.66-3.56 (m, 1H, H-4_{Xylose}), 3.46-3.38 (m, 1H, H-2_{Xylose}), 3.33-3.24 (m, 1H, H-$5b_{Xylose}$), 3.17 (t, $^3J_{CH2,CH2}$ = 7.6 Hz, 2H, CH_2-CH_2-N), 2.42 (t, $^4J_{CH2,C≡CH}$ = 2.4 Hz, 1H, C≡CH), 2.20 (s, 3H, OAc), 2.05 (s, 3H, OAc), 2.04 (s, 3H, OAc), 2.03 (s, 3H, OAc), 1.46-1.39 (m, 18H, 2 x Boc) ppm.

^{13}C NMR (151 MHz, $CDCl_3$, 298 K): δ = 170.7 (C=$O_{OAc\ at\ C\text{-}6}$), 170.2 (C=$O_{OAc\ at\ C\text{-}2}$), 170.1 (C=$O_{OAc\ at\ C\text{-}3}$), 169.9 (C=$O_{OAc\ at\ C\text{-}4}$), 155.5 (C=O_{Boc}), 155.3 (C=O_{Boc}), 154.9 ($C_{ipso\text{-Phenol}}$), 145.4 (C=$CH_{Triazole}$), 131.8 ($C_{p\text{-Phenol}}$), 130.0 ($C_{m\text{-Phenol}}$), 123.1 (C=$CH_{Triazole}$), 117.1 ($C_{o\text{-Phenol}}$), 98.6 (C-1_{Xylose}), 96.1 (C-1_{Man}), 80.1 (2 x O-$C(CH_3)_3$), 79.0 (CH_2C≡C), 76.9 (C-3_{Xylose}), 75.0 (CH_2C≡C), 69.6 (C-2_{Man}), 69.3 (C-5_{Man}), 69.0 (C-3_{Man}), 66.1 (C-4_{Man}), 64.7 (N-C-CH_2), 62.6 (C-5_{Xylose}), 62.3 (C-6_{Man}), 55.5 (CH_2C≡C), 54.8 (C-2_{Xylose}), 51.8 (CH_2-N), 50.9 (C-4_{Xylose}), 36.0 (CH_2-CH_2-N), 28.5 (2 x Boc), 21.0 ($CH3_{OAc}$), 20.8 (3 x $CH3_{OAc}$) ppm.

1.17 Glycoconjugate 19

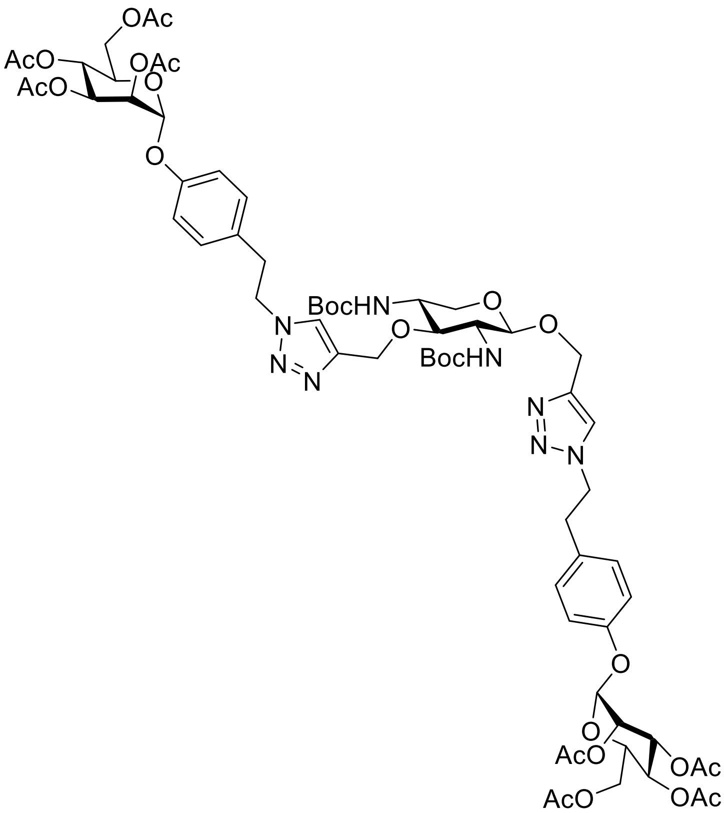

Under a nitrogen atmosphere, 4-(2-azidoethyl)phenyl 2,3,4,6-tetra-*O*-acetyl-α-D-mannopyranoside **14** [7] (72.5 mg, 147 µmol), propargyl xyloside **7** (79.9 mg, 87.0 µmol) and sodium ascorbate (153 mg, 770 µmol) were stirred at room temperature. Degassed tetrahydrofuran (3.0 mL) was added followed by degassed water (760 µL). Anhydrous copper(II)sulfate (42.4 mg, 267 µmol) was added and a black precipitate formed which turned yellow after 1 min. After stirring for 83 min the mixture was diluted with dichloromethane (70 mL) and washed with ethylenediaminetetraacetic acid disodium salt dihydrate solution (15 mL, 10 mM). The organic layer was separated and the aqueous layer was extracted with dichloromethane (15 mL). The combined organic layers were dried over $MgSO_4$, it was filtered and loaded onto Celite®. The crude product was purified on silica gel via automated flash chromatography (dichloromethane:MeOH 100:0 → 90:10). The product showed impurities and was dissolved in dichloromethane and loaded onto Celite®. The crude product was purified on silica gel (cyclohexane:ethyl acetate, 50:50 followed by dichloromethane:MeOH 95:5) to obtain the title compound **19** as colourless solid (93.0 mg, 65.9 µmol, 76%).

$\boldsymbol{R}_\mathrm{F}$ (dichloromethane:methanol, 9:1) = 0.52.

$[\boldsymbol{\alpha}]_\mathbf{D}^{\mathbf{20}}$ = +21.951 (c = 0.12, $CHCl_3$).

IR (**ATR**): $\tilde{\nu}$ = 2975, 1747, 1713, 1511, 1367, 1216, 1036 cm^{-1}.

ESI-HRMS: *m/z* = 1411.56720 $[C_{65}H_{86}N_8O_{27}+H]^+$ (calculated *m/z* = 1411.56752 $[C_{65}H_{86}N_8O_{27}+H]^+$).

^{1}H NMR (600 MHz, $CDCl_3$, 298 K): δ = 7.52 (s, 1H, C=C$\underline{H}_{\text{Triazole A}}$), 7.47 (s, 1H, C=C$\underline{H}_{\text{Triazole B}}$), 7.11-7.06 (m, 4H, 2 x C$\underline{H}_{m\text{-Phenol}}$), 7.04-7.00 (m, 4H, 2 x C$\underline{H}_{o\text{-Phenol}}$), 5.56-5.53 (m, 2H, 2 x H-3_{Man}), 5.49 (d, $^3J_{1,2}$ = 1.9 Hz, 2H, 2 x H-1_{Man}), 5.42 (dd, $^3J_{2,3}$ = 3.5 Hz, $^3J_{1,2}$ = 1.8 Hz, 2H, 2 x H-

2_{Man}), 5.39-5.34 (m, 2H, 2 x H-4_{Man}), 5.07-4.80 (m, 4H , N-C-C<u>H</u>$_2$, H-1_{Xylose}, N<u>H</u>), 4.77-4.66 (m, 3H, N-C-C<u>H</u>$_2$, N<u>H</u>), 4.56-4.47 (m, 4H, 2 x C<u>H</u>$_2$-N), 4.30-4.25 (m , 2H, 2 x H-6a), 4.21-4.14 (m, 1H, H-5_{Xylose}), 4.12-4.04 (m, 4H, 2 x H-6b, 2 x H-5_{Man}), 3.88-3.74 (m, 1H, H-3_{Xylose}), 3.66-3.52 (m, 2H, H-4_{Xylose}, H-2_{Xylose}), 3.33-3.24 (m, 1H, H-5_{Xylose}), 3.20-3.12 (m, 4H, 2 x C<u>H</u>$_2$-CH$_2$-N), 2.20 (s, 3H, OAc$_{\text{at C-2}}$), 2.19 (s, 3H, OAc$_{\text{at C-2}}$), 2.05 (s, 6H, 2 x OAc$_{\text{at C-4}}$), 2.04 (s, 6H, 2 x OAc$_{\text{at C-6}}$), 2.03 (s, 6H, 2 x OAc$_{\text{at C-3}}$), 1.43 (s, 9H, Boc) 1.41 (s, 9H, Boc) ppm.

^{13}C NMR (151 MHz, $CDCl_3$, 298 K): δ = 170.7 (2 x C=O$_{\text{OAc at C-6}}$), 170.18 (C=O$_{\text{OAc at C-2}}$), 170.16 (C=O$_{\text{OAc at C-2}}$), 170.1 (2 x C=O$_{\text{OAc at C-3}}$), 169.9 (2 x C=O$_{\text{OAc at C-4}}$), 154.9 (2 x <u>C</u>$_{ipso\text{-Phenol}}$), 145.2 (2 x <u>C</u>=CH$_{\text{Triazole}}$), 131.8 (<u>C</u>$_{p\text{-Phenol}}$), 131.7 (<u>C</u>$_{p\text{-Phenol}}$), 130.1 (<u>C</u>$_{m\text{-Phenol}}$), 130.0 (<u>C</u>$_{m\text{-Phenol}}$), 123.2 (C=<u>C</u>H$_{\text{Triazole B}}$), 123.0 (C=<u>C</u>H$_{\text{Triazole A}}$), 117.0 (2 x <u>C</u>$_{o\text{-Phenol}}$), 100.0 (C-1_{Xylose}), 96.1 (2 x C-1_{Man}), 76.7 (C-3_{Xylose}), 69.6 (C-2_{Man}), 69.5 (C-2_{Man}), 69.3 (2 x C-5_{Man}), 69.0 (2 x C-3_{Man}), 66.1 (2 x C-4_{Man}), 64.2 (N-C-<u>C</u>H$_{2\text{ at C-3}}$), 62.3 (2 x C-6_{Man}), 62.2 (N-C-<u>C</u>H$_{2\text{ at C-1}}$), 62.0 (C-5_{Xylose}), 53.6 (C-2_{Xylose}), 51.7 (2 x <u>C</u>H$_2$-N), 50.5 (C-4_{Xylose}), 36.1 (<u>C</u>H$_2$-CH$_2$-N), 36.0 (<u>C</u>H$_2$-CH$_2$-N), 28.5 (2 x Boc), 21.0 (2 x <u>C</u>H3$_{\text{OAc}}$), 20.8 (6 x <u>C</u>H3$_{\text{OAc}}$) ppm.

1.18 Glycoconjugate 21

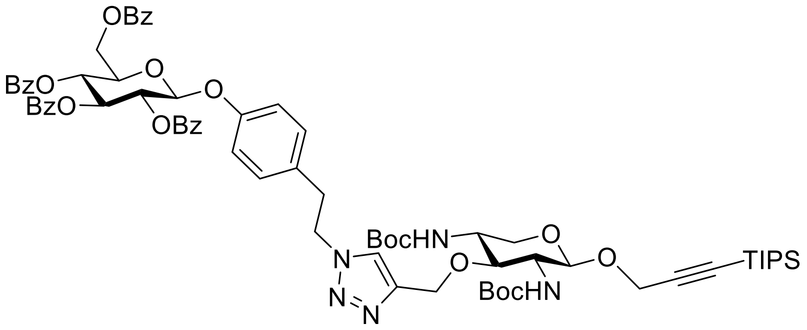

Under a nitrogen atmosphere, 4-(2-azidoethyl)phenyl 2,3,4,6-tetra-*O*-benzoyl-β-D-glucopyranoside **15** (305 mg, 412 µmol), propargyl xyloside **7** (100 mg, 173 µmol), sodium ascorbate (137 mg, 692 µmol) and anhydrous copper(II)sulfate (54.8 mg, 343 µmol) were stirred at room temperature. Degassed tetrahydrofuran (6.0 mL) was added followed by degassed water (1.5 mL). After stirring for 200 min the mixture was diluted with dichloromethane (75 mL) and washed with ethylenediaminetetraacetic acid (EDTA) disodium salt dihydrate solution (15 mL, 0.01 M). The organic layer was separated and the aqueous layer was extracted with dichloromethane (10 mL). The combined organic layers were dried over $MgSO_4$, it was filtered and the residual loaded onto Celite®. The crude product was purified on silica gel (dichloromethane:MeOH 100:0 → 95:5) to obtain the title compound **21** as a colourless solid (209 mg, 158 µmol, 92%).

***R*$_F$** (dichloromethane:MeOH, 19:1) = 0.54.

$[\alpha]_D^{20}$ = +0.667 (c = 0.15, $CHCl_3$).

IR (**ATR**): $\tilde{\nu}$ = 3354, 2948, 2865, 1723, 1602, 1261, 1067, 708 cm^{-1}.

ESI-HRMS: *m/z* = 1322.59349 $[C_{72}H_{87}N_5O_{17}Si+H]^+$ (calculated *m/z* = 1322.59390 $[C_{72}H_{87}N_5O_{17}Si+H]^+$).

^{1}H NMR (600 MHz, $CDCl_3$, 298 K): δ = 8.05-8.01 (m, 2H, OBz$_{\text{ortho}}$), 7.98-7.95 (m, 2H, OBz$_{\text{ortho}}$), 7.95-7.92 (m, 2H, OBz$_{\text{ortho}}$), 7.87-7.84 (m, 2H, OBz$_{\text{ortho}}$), 7.61-7.57 (m, 1H, OBz$_{para}$), 7.55-7.49

(m, 2H, 2 x OBz$_{para}$), 7.48-7.35 (m, 8H, OBz$_{para}$, C=C$\underline{H}_{Triazole}$, 3 x OBz$_{meta}$), 7.33-7.28 (m, 2H, OBz$_{meta}$), 6.98-6.88 (m, 4H, C$\underline{H}_{m\text{-Phenol}}$, C$\underline{H}_{o\text{-Phenol}}$), 5.99 (t, $^3J_{2,3}$ = $^3J_{3,4}$ = 9.5 Hz, 1H, H-3$_{Glc}$), 5.79 (dd, $^3J_{2,3}$ = 9.6 Hz, $^3J_{1,2}$ = 7.7 Hz, 1H, H-2$_{Glc}$), 5.74-5.70 (m, 1H, H-4$_{Glc}$), 5.38 (d, $^3J_{1,2}$ = 7.8 Hz, 1H, H-1$_{Glc}$), 4.98-4.73 (m, 5H, N-C-C$\underline{H}_2$, H-1$_{Xylose}$, 2 x N$\underline{H}$), 4.67 (dd, $^2J_{6a,6b}$ = 12.1 Hz, $^3J_{5,6a}$ = 3.1 Hz, 1H, H-6a$_{Glc}$), 4.55 (dd, $^2J_{6a,6b}$ = 12.1 Hz, $^3J_{5,6b}$ = 6.4 Hz, 1H, H-6b$_{Glc}$), 4.48-4.41 (m, 2H, C$\underline{H}_2$-N), 4.36-4.31 (m, 3H, C$\underline{H}_2$C≡C, H-5$_{Glc}$), 4.18-4.10 (m, 1H, H-5a$_{Xylose}$), 3.82-3.69 (m, 1H, H-3$_{Xylose}$), 3.66-3.54 (m, 2H, H-4$_{Xylose}$, H-2$_{Xylose}$), 3.31-3.21 (m, 1H, H-5b$_{Xylose}$), 3.11 (t, $^3J_{CH2,CH2}$ = $^3J_{CH2,CH2}$ = 7.6 Hz, 2H, C$\underline{H}_2$-CH$_2$-N), 1.47-1.35 (m, 18H, Boc), 1.12-0.98 (m, 21H, TIPS) ppm.

^{13}C NMR (151 MHz, CDCl$_3$, 298 K): δ = 166.2 (C=O$_{OBz\ at\ C\text{-}6}$), 165.9 (C=O$_{OBz\ at\ C\text{-}3}$), 165.4 (C=O$_{OBz\ at\ C\text{-}4}$), 165.2 (C=O$_{OBz\ at\ C\text{-}2}$), 156.1 ($\underline{C}_{ipso\text{-Phenol}}$), 155.4 (C=O$_{Boc}$), 155.3 (C=O$_{Boc}$), 133.7 (OBz$_{para}$), 133.53 (OBz$_{para}$), 133.49 (OBz$_{para}$), 133.47 (OBz$_{para}$), 132.0 ($\underline{C}_{p\text{-Phenol}}$), 130.04 (OBz$_{ortho}$), 129.98 (OBz$_{ortho}$), 129.95 (OBz$_{ortho}$), 129.9 (OBz$_{ortho}$), 129.8 ($\underline{C}_{m\text{-Phenol}}$), 129.7 (OBz$_{ipso}$), 129.2 (OBz$_{ipso}$), 128.9 (OBz$_{ipso}$), 128.8 (OBz$_{ipso}$), 128.62 (OBz$_{meta}$), 128.58 (2 x OBz$_{meta}$), 128.5 (OBz$_{meta}$), 123.0 (C=$\underline{C}H_{Triazole}$), 117.8 ($\underline{C}_{o\text{-Phenol}}$), 102.3 (CH$_2\underline{C}$≡C), 99.8 (C-1$_{Glc}$), 98.5 (C-1$_{Xylose}$), 88.4 (CH$_2$C≡$\underline{C}$), 77.4 (C-3$_{Xylose}$), 72.9 (C-3$_{Glc}$), 72.7 (C-5$_{Glc}$), 71.9 (C-2$_{Glc}$), 69.8 (C-4$_{Glc}$), 64.4 (N-C-$\underline{C}H_2$), 63.3 (C-6$_{Glc}$), 62.3 (C-5$_{Xylose}$), 56.2 ($\underline{C}H_2$C≡C), 54.1 (C-2$_{Xylose}$), 51.7 ($\underline{C}H_2$-N), 50.5 (C-4$_{Xylose}$), 36.1 ($\underline{C}H_2$-CH$_2$-N), 28.5 (Boc), 18.7 (TIPS), 11.3 (TIPS) ppm.

1.19 Glycoconjugate 22

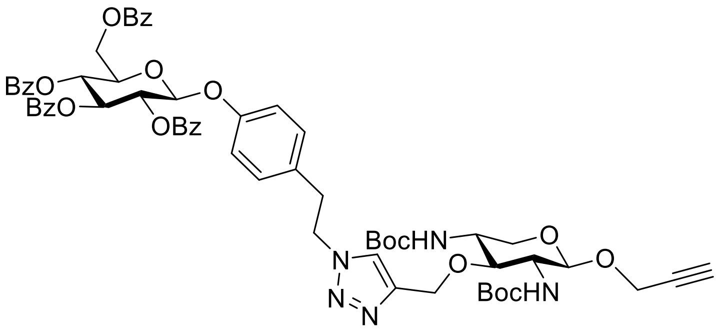

The xyloside **21** (208 mg, 157 µmol) was dissolved in tetrahydrofuran (11.6 mL) and the solution cooled to 0 °C. Tetrabutylammonium fluoride (1 M in tetrahydrofuran, 160 µL, 160 µmol) was added dropwise. The mixture was further stirred at 0 °C for 30 min. The reaction mixture was diluted with dichloromethane (120 mL) and washed with brine (30 mL). The aqueous layer was separated and extracted with dichloromethane (20 mL). The combined organic layers were dried over MgSO$_4$, it was filtered and the residual loaded onto Celite®. The crude product was purified on silica gel (cyclohexane:ethyl acetate, 60:40 → 15:85) to obtain the title compound **22** as a colourless solid (153 mg, 131 µmol, 84%).

***R*$_F$** (cyclohexane:ethyl acetate, 3:7) = 0.70.

$[\alpha]_D^{20}$ = -0.546 (c = 0.18, CHCl$_3$).

IR (ATR): $\tilde{\nu}$ = 3313, 2976, 1723, 1511, 1261, 708 cm^{-1}.

ESI-HRMS: *m/z* = 1164.44535 [C$_{63}$H$_{67}$N$_5$O$_{17}$+H]$^+$ (calculated *m/z* = 1164.44482 [C$_{63}$H$_{67}$N$_5$O$_{17}$+H]$^+$).

^{1}H NMR (600 MHz, CDCl$_3$, 298 K): δ = 8.05-8.01 (m, 2H, OBz$_{ortho}$), 7.98-7.94 (m, 2H, OBz$_{ortho}$), 7.94-7.91 (m, 2H, OBz$_{ortho}$), 7.87-7.83 (m, 2H, OBz$_{ortho}$), 7.61-7.57 (m, 1H, OBz$_{para}$), 7.54-7.50

(m, 2H, 2 x OBz_{para}), 7.47-7.35 (m, 8H, OBz_{para}, C=$CH_{Triazole}$, 3 x OBz_{meta}), 7.33-7.28 (m, 2H, OBz_{meta}), 6.96-6.88 (m, 4H, $CH_{m\text{-}Phenol}$, $CH_{o\text{-}Phenol}$), 5.99 (t, $^3J_{2,3}$ = $^3J_{3,4}$ = 9.5 Hz, 1H, H-3_{Glc}), 5.79 (dd, $^3J_{2,3}$ = 9.6 Hz, $^3J_{1,2}$ = 7.7 Hz, 1H, H-2_{Glc}), 5.72 (t, $^3J_{3,4}$ = $^3J_{4,5}$ = 9.7 Hz, 1H, H-4_{Glc}), 5.38 (d, $^3J_{1,2}$ = 7.7 Hz, 1H, H-1_{Glc}), 5.00-4.73 (m, 5H, N-C-CH_2, H-1_{Xylose}, 2 x NH), 4.67 (dd, $^2J_{6a,6b}$ = 12.1 Hz, $^3J_{5,6a}$ = 3.1 Hz, 1H, H-$6a_{Glc}$), 4.55 (dd, $^2J_{6a,6b}$ = 12.1 Hz, $^3J_{5,6b}$ = 6.5 Hz, 1H, H-$6b_{Glc}$), 4.48-4.41 (m, 2H, CH_2-N), 4.34 (ddd, $^3J_{4,5}$ = 9.7 Hz, $^3J_{5,6b}$ = 6.4 Hz, $^3J_{5,6a}$ = 3.1 Hz, 1 H, H-5_{Glc}), 4.34-4.26 (m, 2H, CH_2C≡C), 4.15-4.08 (m, 1H, H-5_{Xylose}), 3.96-3.82 (m, 1H, H-3_{Xylose}), 3.65-3.56 (m, 1H, H-4_{Xylose}), 3.45-3.36 (m, 1H, H-2_{Xylose}), 3.33-3.24 (m, 1H, H-5_{Xylose}), 3.10 (t, $^3J_{CH2,CH2}$ = $^3J_{CH2,CH2}$ = 7.6 Hz, 2H, CH_2-CH_2-N), 2.42 (t, $^4J_{CH2,C≡CH}$ = 2.4 Hz, 1H, C≡CH), 1.43 (m, 9H, Boc), 1.41 (m, 9H, Boc), ppm.

^{13}C NMR (151 MHz, $CDCl_3$, 298 K): δ = 166.2 (C=$O_{OBz\ at\ C\text{-}6}$), 165.9 (C=$O_{OBz\ at\ C\text{-}3}$), 165.4 (C=$O_{OBz\ at\ C\text{-}4}$), 165.2 (C=$O_{OBz\ at\ C\text{-}2}$), 156.0 ($C_{ipso\text{-}Phenol}$), 155.4 (C=O_{Boc}), 155.3 (C=O_{Boc}), 145.2 (N-C-CH_2), 133.7 (OBz_{para}), 133.53 (OBz_{para}), 133.5 (OBz_{para}), 133.47 (OBz_{para}), 132.0 ($C_{p\text{-}Phenol}$), 130.04 (OBz_{ortho}), 129.98 (OBz_{ortho}), 129.95 (OBz_{ortho}), 129.92 (OBz_{ortho}), 129.86($C_{m\text{-}Phenol}$), 129.7 (OBz_{ipso}), 129.2 (OBz_{ipso}), 128.9 (OBz_{ipso}), 128.8 (OBz_{ipso}), 128.63 (OBz_{meta}), 128.59 (2 x OBz_{meta}), 128.5 (OBz_{meta}), 123.1 (C=$CH_{Triazole}$), 117.8 ($C_{o\text{-}Phenol}$), 99.7 (C-1_{Glc}), 98.6 (C-1_{Xylose}), 79.0 (CH_2C≡C), 76.9 (C-3_{Xylose}), 75.0 (CH_2C≡C), 72.9 (C-3_{Glc}), 72.7 (C-5_{Glc}), 71.9 (C-2_{Glc}), 69.8 (C-4_{Glc}), 64.8 (N-C-CH_2), 63.3 (C-6_{Glc}), 62.7 (C-5_{Xylose}), 55.5 (CH_2C≡C), 54.9 (C-2_{Xylose}), 51.7 (CH_2-N), 51.0 (C-4_{Xylose}), 36.1 (CH_2-CH_2-N), 29.1 (Boc) ppm.

1.20 Glycoconjugate 23

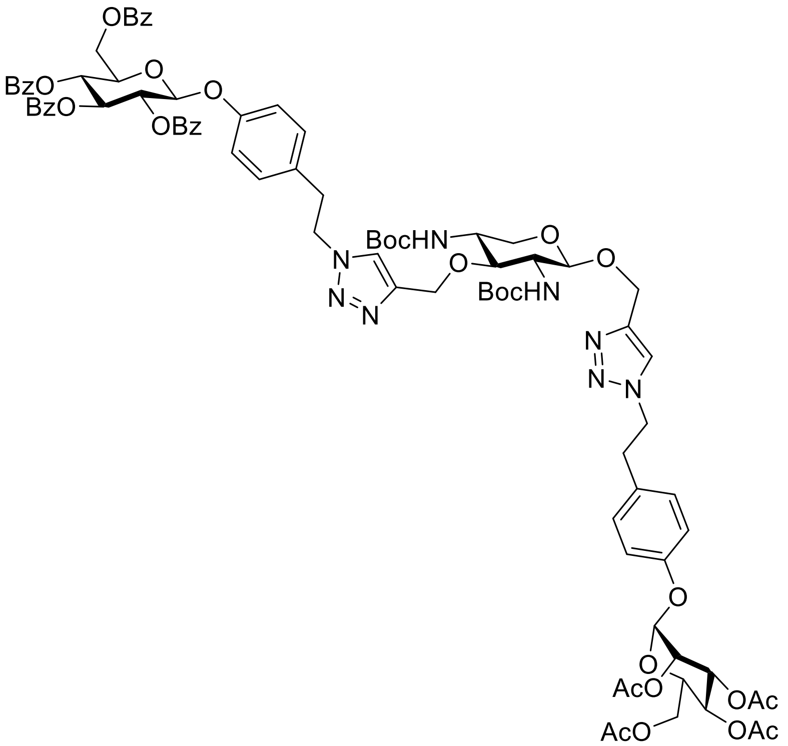

Under a nitrogen atmosphere, 4-(2-azidoethyl)phenyl 2,3,4,6-tetra-*O*-acetyl-α-D-mannopyranoside **14** [7] (137 mg, 277 µmol), propargyl xyloside **22** (135 mg, 116 µmol), sodium ascorbate (102 mg, 517 µmol) and anhydrous copper(II)sulfate (37.7 mg, 236 µmol) were stirred at room temperature. Degassed tetrahydrofuran (4.0 mL) was added followed by degassed water (1.0 mL). After stirring for 130 min the mixture was diluted with

dichloromethane (75 mL) and washed with ethylenediaminetetraacetic acid disodium salt dihydrate solution (15 mL, 0.01 M). The organic layer was separated and the aqueous layer was extracted with dichloromethane (10 mL). The combined organic layers were dried over $MgSO_4$, it was filtered and the residual loaded onto Celite®. The crude product was purified on silica gel (cyclohexane:ethyl acetate, 50:50, 3.7 column volumes followed by dichloromethane:MeOH 100:0 → 90:10) to obtain the title compound **23** as colourless foam (154 mg, 93.0 µmol, 80%).

$\boldsymbol{R}_\mathbf{F}$ (dichloromethane:methanol, 9:1) = 0.36.

$[\boldsymbol{\alpha}]_\mathbf{D}^{\mathbf{20}}$ = +15.789 (c = 0.23, $CHCl_3$).

IR (**ATR**): $\tilde{\nu}$ = 3348, 2973, 1732, 1511, 1220, 1067, 710 cm^{-1}.

ESI-HRMS: *m/z* = 1703.62238 $[C_{85}H_{94}N_8O_{27}+CHO_2]^-$ (calculated *m/z* = 1703.61995 $[C_{85}H_{94}N_8O_{27}+CHO_2]^-$).

^{1}H NMR (600 MHz, $CDCl_3$, 298 K): δ = 8.04-8.00 (m, 2H, OBz_{ortho}), 7.97-7.91 (m, 4H, 2 x OBz_{ortho}), 7.86-7.83 (m, 2H, OBz_{ortho}), 7.59-7.56 (m, 1H, OBz_{para}), 7.55-7.47 (m, 3H, 2 x OBz_{para}, C=C<u>H</u>$_{Triazole}$), 7.47-7.34 (m, 8H, OBz_{para}, 3 x OBz_{meta}, C=C<u>H</u>$_{Triazole}$), 7.32-7.28 (m, 2H, OBz_{meta}), 7.10-6.99 (m, 4H, C<u>H</u>$_{m\text{-Phenol at C-1}}$, C<u>H</u>$_{o\text{-Phenol at C-1}}$), 6.96-6.88 (m, 4H, C<u>H</u>$_{m\text{-Phenol at C-3}}$, C<u>H</u>$_{o\text{-Phenol at C-3}}$), 5.99 (t, $^3J_{2,3}$ = $^3J_{3,4}$ = 9.5 Hz, 1H, H-3_{Glc}), 5.79 (dd, $^3J_{2,3}$ = 9.6 Hz, $^3J_{1,2}$ = 7.7 Hz, 1H, H-2_{Glc}), 5.72 (t, $^3J_{3,4}$ = $^3J_{4,5}$ = 9.6 Hz, 1H, H-4_{Glc}), 5.55 (dd, $^3J_{3,4}$ = 10.0 Hz, $^3J_{2,3}$ = 3.5 Hz, 1H, H-3_{Man}), 5.48 (d, $^3J_{1,2}$ = 1.9 Hz, 1H, H-1_{Man}), 5.42 (dd, $^3J_{2,3}$ = 3.5 Hz, $^3J_{1,2}$ = 1.8 Hz, 1H, H-2_{Man}), 5.39 (d, $^3J_{1,2}$ = 7.7 Hz, 1H, H-1_{Glc}), 5.37 (t, $^3J_{3,4}$ = $^3J_{4,5}$ = 10.1 Hz, 1H, H-4_{Man}), 5.01-4.88 (m, 2H, 2 x N<u>H</u>), 4.86 (d, $^2J_{CHH',CHH'}$ = 12.7 Hz, 1H, N-C-C<u>H</u>$_{2\text{ at C-1}}$), 4.81 (d, $^2J_{CHH',CHH'}$ = 12.3 Hz, 1H, N-C-C<u>H</u>$_{2\text{ at C-3}}$), 4.75-4.64 (m, 3H, N-C-C<u>H</u>$_{2\text{ at C-3}}$, N-C-C<u>H</u>$_{2\text{ at C-1}}$, H-1_{Xylose}), 4.67 (dd, $^2J_{6a,6b}$ = 12.1 Hz, $^3J_{5,6a}$ = 3.1 Hz, 1H, H-$6a_{Glc}$), 4.55 (dd, $^2J_{6a,6b}$ = 12.1 Hz, $^3J_{5,6b}$ = 6.4 Hz, 1H, H-$6b_{Glc}$), 4.53-4.48 (m, 2H, C<u>H</u>$_2$-N), 4.47-4.40 (m, 2H, C<u>H</u>$_2$-N), 4.35 (ddd, $^3J_{4,5}$ = 9.7 Hz, $^3J_{5,6b}$ = 6.4 Hz, $^3J_{5,6a}$ = 3.1 Hz, 1H, H-5_{Glc}), 4.29-4.25 (m, 1H, H-$6a_{Man}$), 4.19-4.05 (m, 3H, H-$5a_{Xylose}$, H-5_{Man}, H-$6b_{Man}$), 3.87-3.75 (m, 1H, H-3_{Xylose}), 3.66-3.58 (m, 1H, H-4_{Xylose}), 3.57-3.50 (m, 1H, H-2_{Xylose}), 3.32-3.24 (m, 1H, H-$5b_{Xylose}$), 3.15 (t, $^3J_{CH2,CH2}$ = 7.6 Hz, 2H, C<u>H</u>$_2$-CH_2-N), 3.09 (t, $^3J_{CH2,CH2}$ = $^3J_{CH2,CH2}$ = 7.6 Hz, 2H, C<u>H</u>$_2$-CH_2-N), 2.19 (s, 3H, OAc$_{\text{ at C-2}}$), 2.05 (s, 3H, OAc$_{\text{ at C-4}}$), 2.034 (s, 3H, OAc), 2.030 (s, 3H, OAc), 1.42 (s, 9H, Boc), 1.40 (s, 9H, Boc) ppm.

^{13}C NMR (151 MHz, $CDCl_3$, 298 K): δ = 170.7 (C=$O_{\text{OAc at C-6}}$), 170.2 (C=$O_{\text{OAc at C-2}}$), 170.1 (C=$O_{\text{OAc at C-3}}$), 169.9 (C=$O_{\text{OAc at C-4}}$), 166.2 (C=$O_{\text{OBz at C-6}}$), 165.9 (C=$O_{\text{OBz at C-3}}$), 165.4 (C=$O_{\text{OBz at C-4}}$), 165.2 (C=$O_{\text{OBz at C-2}}$), 156.0 (<u>C</u>$_{ipso\text{-Phenol at C-3}}$), 155.4, 155.3, 154.9 (<u>C</u>$_{ipso\text{-Phenol at C-1}}$), 145.2 (N-<u>C</u>-$CH_{2\text{ at C-3}}$), 144.9 (N-<u>C</u>-$CH_{2\text{ at C-1}}$), 133.7 ($OBz_{para}$), 133.5 ($OBz_{para}$), 133.5 ($OBz_{para}$), 133.5 ($OBz_{para}$), 132.0 (<u>C</u>$_{p\text{-Phenol}}$), 131.8 (<u>C</u>$_{p\text{-Phenol}}$), 130.0, 129.98, 129.94, 129.92, 129.87, 129.7 (OBz_{ipso}), 129.2 (OBz_{ipso}), 128.9 (OBz_{ipso}), 128.8 (OBz_{ipso}), 128.62 (OBz_{meta}), 128.59 (2 x OBz_{meta}), 128.5 (OBz_{meta}), 123.2 (C=<u>C</u>H$_{Triazole}$), 123.0 (C=<u>C</u>H$_{Triazole}$), 117.7 (<u>C</u>$_{o\text{-Phenol at C-3}}$), 117.0 (<u>C</u>$_{o\text{-Phenol at C-1}}$), 99.9 (C-$1_{Xylose}$), 99.7 (C-$1_{Glc}$), 96.1 (C-$1_{Man}$), 76.8 (C-$3_{Xylose}$), 72.9 (C-$3_{Glc}$), 72.7 (C-$5_{Glc}$), 71.9 (C-$2_{Glc}$), 69.8 (C-$4_{Glc}$), 69.6 (C-$2_{Man}$), 69.3 (C-$5_{Man}$), 69.0 (C-$3_{Man}$), 66.1 (C-$4_{Man}$), 64.3 (N-C-<u>C</u>H$_{2\text{ at C-3}}$), 63.3 (C-$6_{Glc}$), 62.25 (C-$6_{Man}$), 62.2 (N-C-<u>C</u>H$_{2\text{ at C-1}}$), 62.0 (C-$5_{Xylose}$), 53.8 (C-$2_{Xylose}$), 51.7 (<u>C</u>H$_2$-N), 50.6 (C-$4_{Xylose}$), 36.1 (<u>C</u>H$_2$-$CH_2$-N), 28.5 (Boc), 21.0 (CH3$_{\text{ at C-2}}$), 20.8 (3 x CH3$_{\text{ at C-3, at C-4, at C-6}}$) ppm.

1.21 Glycoconjugate 25

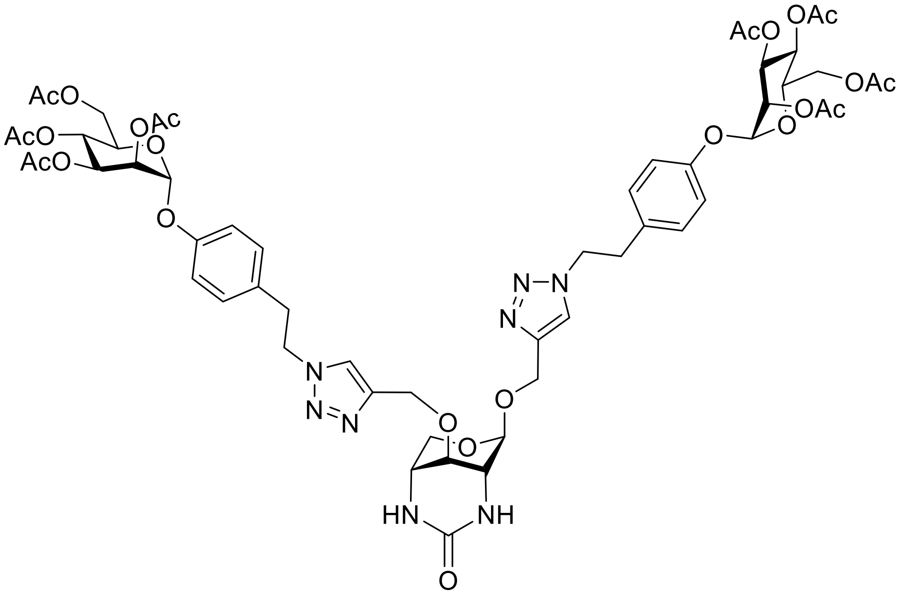

Under a nitrogen atmosphere, 4-(2-azidoethyl)phenyl 2,3,4,6-tetra-*O*-acetyl-α-D-mannopyranoside (**14**) [7] (65.3 mg, 132 µmol), propargyl xyloside **10** (19.6 mg, 78.3 µmol), sodium ascorbate (63.5 mg, 321 µmol) and anhydrous copper(II)sulfate (25.7 mg, 161 µmol) were stirred at room temperature. Degassed tetrahydrofuran (2.7 mL) was added followed by degassed water (680 µL). After 30 min additional mannopyranoside **14** [7] (29.9 mg, 60.6 µmol) dissolved in degassed tetrahydrofuran (1.0 mL) was added. After stirring for 14 h 40 min at room temperature the orange mixture was diluted with dichloromethane (to a total volume of 100 mL) and washed with ethylenediaminetetraacetic acid disodium salt dihydrate solution (20 mL, 0.01 M) followed by brine (3 x 20 mL). The organic layer was dried over $MgSO_4$, it was filtered and the residual loaded onto Celite®. The crude product was purified on silica gel (cyclohexane:ethyl acetate 50:50, then CH_2Cl_2:MeOH 100:0 → 90:10) to obtain the title compound **25** as colourless solid (63.3 mg, 51.2 µmol, 65%).

$\boldsymbol{R}_{\mathbf{F}}$ (dichloromethane:methanol, 9:1) = 0.40.

$[\boldsymbol{\alpha}]_{\mathbf{D}}^{\mathbf{20}}$ = +16.336 (c = 0.20, $CHCl_3$).

IR (**ATR**): $\tilde{\nu}$ = 2941, 1743, 1674, 1510, 1213, 1033, 983 cm^{-1}.

ESI-HRMS: *m/z* = 1275.39647 $[C_{56}H_{68}N_8O_{24}+K]^+$ (calculated *m/z* = 1275.39780 $[C_{56}H_{68}N_8O_{24}+K]^+$).

^{1}H NMR (600 MHz, $CDCl_3$, 298 K): δ = 7.39 (s, 1H, C=C$\underline{\text{H}}_{\text{Triazole at C-1}}$), 7.38 (s, 1H, C=C$\underline{\text{H}}_{\text{Triazole at C-3}}$), 7.09-6.95 (m, 8H, 4 x C$\underline{\text{H}}_{m\text{-Phenol}}$, 4 x C$\underline{\text{H}}_{o\text{-Phenol}}$), 5.55-5.51 (m, 2H, 2 x H-3_{Man}), 5.49 (d, $^3J_{1,2}$ = 1.9 Hz, 1H, H-1_{Man}), 5.48 (d, $^3J_{1,2}$ = 1.9 Hz, 1H, H-1_{Man}), 5.41-5.39 (m, 2H, 2 x H-2_{Man}), 5.40-5.35 (m, 2H, 2 x H-4_{Man}), 5.25-5.21 (m, 1H, N$\underline{\text{H}}_{\text{at C-4}}$), 5.18-5.14 (m, 1H, N$\underline{\text{H}}_{\text{at C-2}}$), 4.83 (d, $^2J_{\text{CH}\underline{\text{H}}'}$ = 11.5 Hz, 1H, N-C-C$\underline{\text{H}}$H'$_{\text{at C-1}}$), 4.77 (s, 1H, H-1_{Xylose}), 4.73 (d, $^2J_{\text{CH}\underline{\text{H}}'}$ = 11.5 Hz, 1H, N-C-C$\underline{\text{H}}$H'$_{\text{at C-3}}$), 4.59 (d, $^2J_{\text{C}\underline{\text{H}}\text{H}'}$ = 11.4 Hz, 1H, N-C-CH$\underline{\text{H}}$'$_{\text{at C-3}}$), 4.59-4.52 (m, 5H, 2 x C$\underline{\text{H}}_2$-N, N-C-CH$\underline{\text{H}}$'$_{\text{at C-1}}$), 4.29-4.24 (m, 2H, H-6a_{Man}, H-6a'$_{\text{Man}}$), 4.13-4.04 (m, 5H, H-$5\text{a}_{\text{Xylose}}$, H-$5_{\text{Man}}$, H-5'$_{\text{Man}}$, H-$6\text{b}_{\text{Man}}$, H-6b'$_{\text{Man}}$), 3.98 (t, $^3J_{2,3}$ = $^3J_{3,4}$ = 3.7 Hz, 1H, H-3_{Xylose}), 3.60-3.56 (m, 1H, H-2_{Xylose}), 3.39-3.32 (m, 2H, H-$5\text{b}_{\text{Xylose}}$, H-$4_{\text{Xylose}}$), 3.18 (t, $^3J_{\text{CH2,CH2}}$ = 7.3 Hz, 2H, C$\underline{\text{H}}_2$-CH_2-N), 3.15 (t, $^3J_{\text{CH2,CH2}}$ = 7.2 Hz, 2H, C$\underline{\text{H}}_2$-CH_2-N), 2.201 (s, 3H, OAc), 2.199 (s, 3H, OAc), 2.06 (s, 3H, OAc), 2.05 (s, 3H, OAc), 2.04 (s, 6H, 2 x OAc), 2.03 (s, 6H, 2 x OAc) ppm.

^{13}**C NMR** (151 MHz, $CDCl_3$, 298 K): δ = 170.7 (2 x $C{=}O_{\text{OAc at C-6}}$), 170.3 (2 x $C{=}O_{\text{OAc at C-2}}$), 170.22 ($C{=}O_{\text{OAc at C-3 or C-3'}}$), 170.18 ($C{=}O_{\text{OAc at C-3 or C-3'}}$), 169.9 (2 x $C{=}O_{\text{OAc at C-4}}$), 157.1 ($\underline{C}{=}O$), 154.9 ($\underline{C}_{\textit{ipso}\text{-Phenol}}$), 154.8 ($\underline{C}_{\textit{ipso}\text{-Phenol}}$), 144.4 ($\underline{C}{=}CH_{\text{Triazole at C-1}}$), 144.2 ($\underline{C}{=}CH_{\text{Triazole at C-3}}$), 131.8 (2 x $\underline{C}_{\textit{p}\text{-Phenol}}$), 130.13 ($\underline{C}_{\textit{m}\text{-Phenol}}$), 130.08 ($\underline{C}_{\textit{m}\text{-Phenol}}$), 123.8 ($C{=}\underline{C}H_{\text{Triazole at C-1}}$), 123.6 ($C{=}\underline{C}H_{\text{Triazole at C-3}}$), 117.1 ($\underline{C}_{\textit{o}\text{-Phenol}}$), 117.0 ($\underline{C}_{\textit{o}\text{-Phenol}}$), 100.2 ($C\text{-}1_{\text{Xylose}}$), 96.03 ($C\text{-}1_{\text{Man}}$), 95.99 ($C\text{-}1_{\text{Man}}$), 69.8 ($C\text{-}3_{\text{Xylose}}$), 69.6 (2 x $C\text{-}2_{\text{Man}}$), 69.3 (2 x $C\text{-}5_{\text{Man}}$), 69.0 (2 x $C\text{-}3_{\text{Man}}$), 66.0 (2 x $C\text{-}4_{\text{Man}}$), 62.5 ($N\text{-}C\text{-}\underline{C}H_{2\text{ at C-3}}$), 62.2 (2 x $C\text{-}6_{\text{Man}}$), 61.3 ($N\text{-}C\text{-}\underline{C}H_{2\text{ at C-1}}$), 60.6 ($C\text{-}5_{\text{Xylose}}$), 51.7 (2 x $\underline{C}H_2\text{-}N$), 48.4 ($C\text{-}4_{\text{Xylose}}$), 47.4 ($C\text{-}2_{\text{Xylose}}$), 36.0 (2 x $\underline{C}H_2\text{-}CH_2\text{-}N$), 21.1 (2 x $CH3_{\text{at C-2}}$), 20.8 (6 x CH3) ppm.

2 Conformational analysis

NMR spectra exhibiting broadened peaks are difficult to analyse, as key information such as coupling constants may be obscured, and reliable signal assignment becomes challenging. Not only 1D NMR but also 2D NMR spectra are affected making analysis complicated. Line broadening typically arises when molecular dynamics occur on the timescale of the NMR measurement. A prominent example are carbamate rotamers which have been observed and described before in *tert*-butyloxycarbonyl protected amino sugars [8]. The amino functions of the xylosides reported in this paper, except for the locked $^{1}C_{4}$ conformation, are *tert*-butyloxycarbonyl protected. The NMR spectra of these compounds show broadened signals of the central xyloside unit. The question of whether the line broadening is caused by conformational dynamics of the xyloside ring or by carbamate rotamers can be clarified based on the presence of sharp signals for the anomeric protons of compounds **4** and **5**. In the $^{4}C_{1}$ conformation, a coupling constant for the $^{3}J_{1,2}$ of 2-4 Hz is expected for the α-xyloside **4** and indeed, 3.8 Hz are observed. For the β-xyloside **5**, a larger $^{3}J_{1,2}$ coupling constant in the range of 6-9 Hz is expected and indeed, 7.1 Hz was observed, both being consistent with the $^{4}C_{1}$ conformation of the central xyloside ring. In the ^{1}H NMR spectrum of compound **7**, the anomeric proton signal overlapped with the carbamate signals, preventing determination of the coupling constant. Therefore, a variable temperature (VT) NMR experiment was performed with compound **7**, shown in Figure S1. The H-3 proton exhibits a coalescence temperature of approximately 277 K [9]. Above this temperature, the H-3 proton signal appears as a single broad signal. However, at 248 K the signal resolves into two relative sharp triplets. These two triplets, observed at 3.83 ppm and 3.75 ppm, are present in a ratio of 3:1 and have coupling constants of 7.2 Hz and 7.1 Hz, respectively (Figure S2). These values indicate that H-3 couples with two *trans*-diaxial vicinal protons, consistent with a $^{4}C_{1}$ conformation of the xyloside ring. In contrast, a $^{1}C_{4}$ conformation would result in *trans*-diequatorial vicinal couplings, which would exhibit significantly smaller coupling constants. The observed 3:1 ratio of the two signals is also consistent with previously reported data on *cis*-*trans* rotamers [10]. This ratio is strongly dependent on the solvent as well as on the substitution pattern of the scaffold as showcased in the literature for a glucosamine derivative [11]. Based on this analysis and on the absence of any inconsistencies in all other NMR spectra, the observed behaviour is attributed to carbamate rotamers and not to conformational dynamics of the xyloside ring.

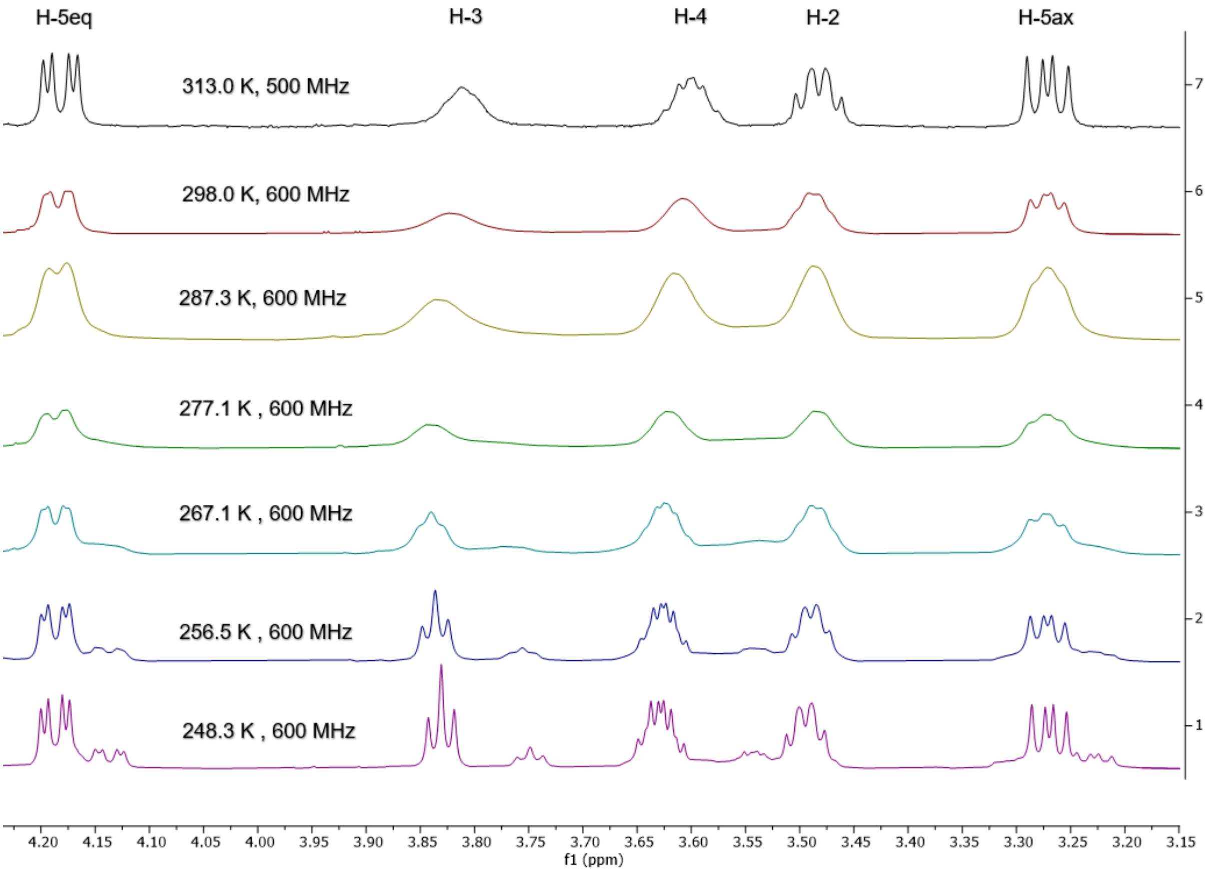

Figure S1 ^{1}H NMR spectra of compound **7** recorded at different temperatures.

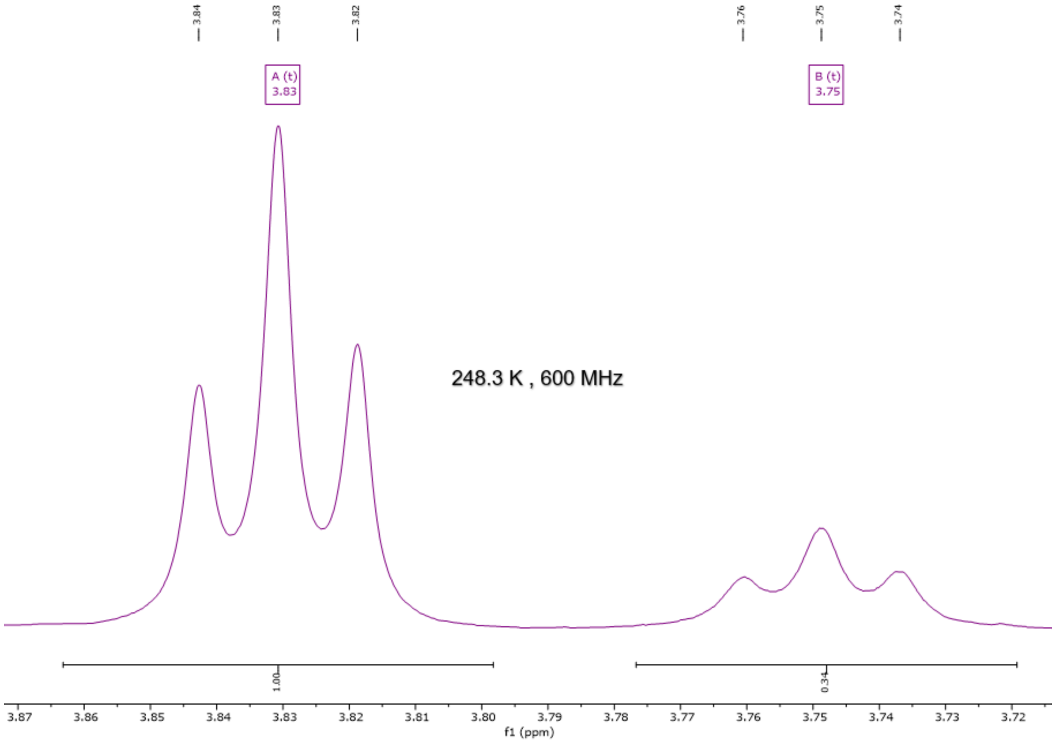

Figure S2 ^{1}H NMR spectra of compound **7** in the region where the H-3 proton is located.

3 Biological testing

Equipment

The equipment was sterilised in a biomedis 3870EN-C6-D autoclave before usage. To determine the optical density of the bacterial suspension, a Jenway Spectrophotometer Model 7305 was used. Washing of 96-well plates was performed on a HyroFlex microplate washer. Fluorescent readout of was performed on an Infinite M Nano+ plate reader.

Buffers and media

For biological assays, the following media and buffer systems were employed. All solutions were prepared using type 1 water from a Purelab® flex 3 system. The solutions were prepared by following procedures:

<u>LB medium</u>: Luria/Miller powder from Roth® (trypton (10.0 g), sodium chloride (10.0 g), yeast extract (5.00 g)) was dissolved in type 1 water (1.00 L). After autoclaving and cooling to ambient temperature, ampicillin (100 mg L^{-1}) and chloramphenicol (50.0 mg L^{-1}) were added under sterile conditions.

<u>PBS buffer (pH 7.2)</u>: 2 PBS tablets (gibco™) were dissolved in type 1 water (1.00 L) to afford a buffer solution of pH 7.2; (buffer composition: sodium phosphate 10 mM, potassium chloride 2.68 mM, sodium chloride 140 mM).

<u>PBST buffer</u>: Tween 20 (0.05% v/v) was added to PBS buffer and mixed thoroughly.

<u>Carbonate buffer (pH 9.6)</u>: Sodium carbonate (1.59 g) and sodium hydrogen carbonate (2.52 g) were dissolved in type 1 water (1.00 L) to afford a buffer solution of pH 9.6.

Bacteria

For the binding assays, the GFP-expressing *Escherichia coli* strain PKL1162, generated in the laboratory of Per Klemm, was employed [12]. Strain PKL1162 was constructed by introduction of the plasmid pPKL1174 into the parental strain SAR18 [13]. The plasmid pPKL1174 harbours the *fim* gene cluster responsible for the expression of type 1 fimbriae, whereas SAR18 carries a chromosomally integrated *gfp* gene under the control of a constitutive promoter [13]. Consequently, the resulting strain PKL1162 exclusively expresses type 1 fimbriae and a green fluorescent protein (GFP), thereby enabling fluorescence-based detection in binding assays.

Adhesion inhibition assay

<u>Functionalization of microtitre plates</u>: A previously reported assay [12] was adapted as follows: Black 96-well microtitre plates (Nunc MaxiSorp 96-well plate) were incubated at 37 °C and 170 rpm of shaking over night with a solution of mannan derived from *Saccharomyces cerevisiae* (1.2 mg mL^{-1} in carbonate buffer, 120 µL per well). Subsequently, the wells were washed with PBST buffer (3 × 400 µL per well) and blocked with poly(vinyl alcohol) (PVA, 1% w/v in PBS buffer, 120 µL per well) at 37 °C and 100 rpm for 2 h. After blocking, the plates were washed again with PBST buffer (3 × 400 µL per well) prior to further use.

Bacterial cultivation: *Escherichia coli* (PKL1162) was cultivated overnight in LB medium + AMP + CAM (20 mL, 100 mg ampicillin, 50 mg chloramphenicol/L) at 37 °C with shaking at 170 rpm. Cells were harvested by centrifugation at 5000 rpm for 10 min, washed twice with PBS buffer, and resuspended to a final concentration of 2 mg/mL for subsequent binding assays.

Assay: 10 steps 1:2 serial dilutions of the inhibitors (in PBS buffer + 5% v/v DMSO, 50 µL per well) were prepared in the 96-well plates. The bacterial suspension (50 µL per well) was added, and the plates were incubated at 100 rpm and 37 °C for 45 min. After incubation, the wells were washed with PBS buffer (3 × 400 µL) to remove unbound bacteria. The wells were then filled with PBS buffer + 5% DMSO (100 µL/well), and fluorescence intensity was recorded (λ_{ex} = 485 nm, λ_{em} = 535 nm). On each plate, the standard inhibitor methyl α-D-mannopyranoside (MeMan) was included in triplicate as a reference, while the glycans were tested in duplicate or triplicate.

Inhibition curves obtained from adhesion–inhibition assays with *E. coli*

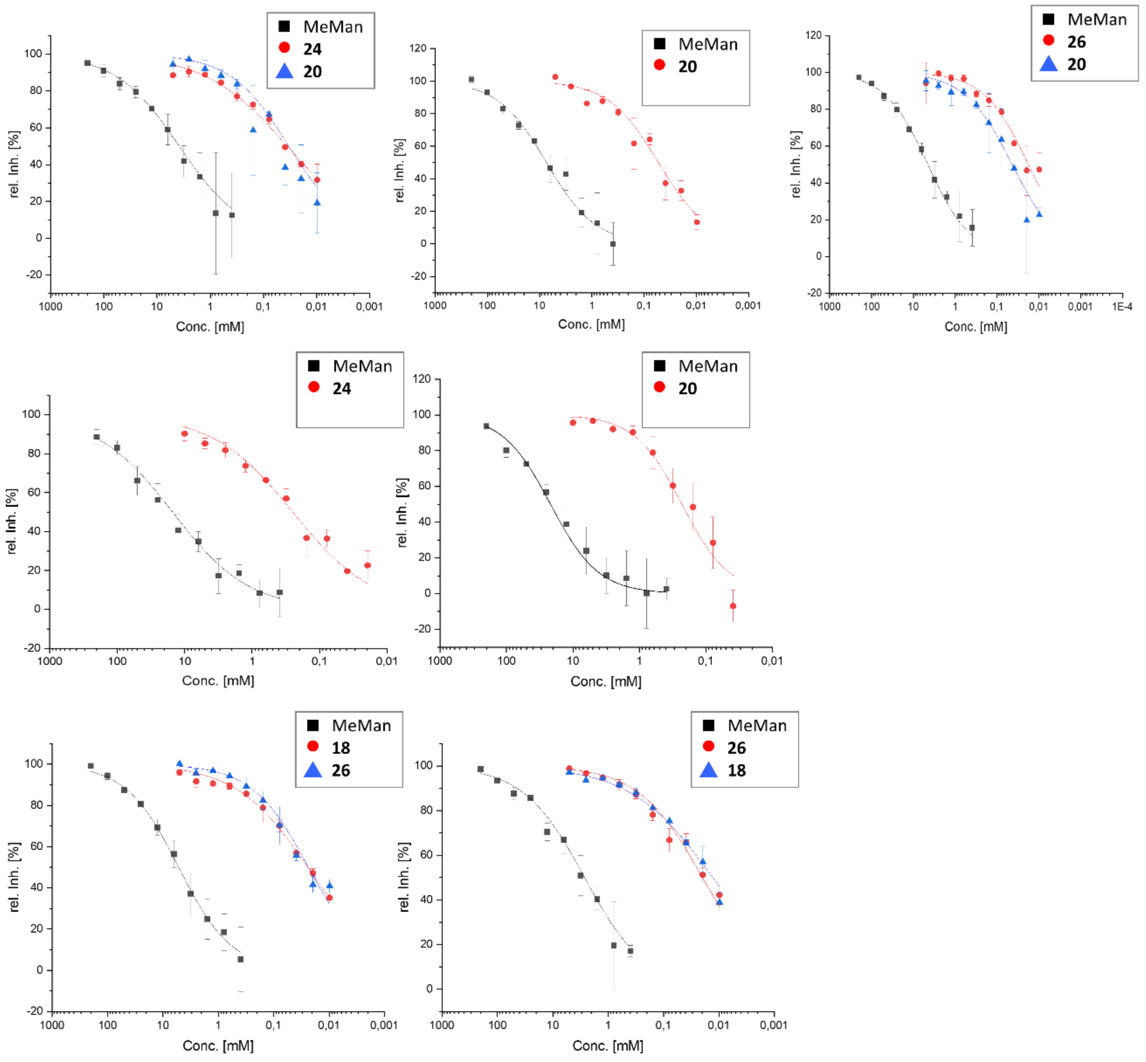

Figure S3 Representative inhibition curves obtained from the evaluation of glycoclusters **18**, **20**, **24** and **26** as inhibitors of type 1 fimbriae-mediated bacterial adhesion to mannan. Methyl α-D-mannopyranoside (MeMan) was included as a reference inhibitor on each plate. Sigmoidal concentration–response curves were obtained by nonlinear regression analysis. Error bars represent standard deviations of triplicate or duplicate determinations performed on a single plate.

IC_{50} values were determined by plotting fluorescence intensity versus inhibitor concentration and sigmoidal fitting of the data.

Determination of errors

Errors in RIP values were determined by error propagation with the following formula:

$$\Delta \mathrm{RIP} = \left|\left(\frac{1}{\mathrm{IC(glycan)}}\right) \cdot \Delta \mathrm{IC_{50}(MeMan)}\right| + \left|-\left(\frac{\mathrm{IC_{50}(MeMan)}}{\mathrm{IC_{50}(glycan)}^2}\right) \cdot \Delta \mathrm{IC_{50}(glycan)}\right|$$

Average RIP values of two independent experiments (A, B) are given with error propagation using the following formula:

$$\Delta \mathrm{Mean\ RIP} = \frac{1}{2} \cdot \sqrt{(\Delta \mathrm{RIP_A})^2 + (\Delta \mathrm{RIP_B})^2}$$

Average RIP values of three independent experiments (A, B, C) are given with error propagation using the following formula:

$$\Delta \mathrm{Mean\ RIP} = \frac{1}{3} \cdot \sqrt{(\Delta \mathrm{RIP_A})^2 + (\Delta \mathrm{RIP_B})^2 + (\Delta \mathrm{RIP_C})^2}$$

4 NMR Spectra

The depicted NMR spectra were recorded on an AVANCE 600 spectrometer.

4.1 Compound S2

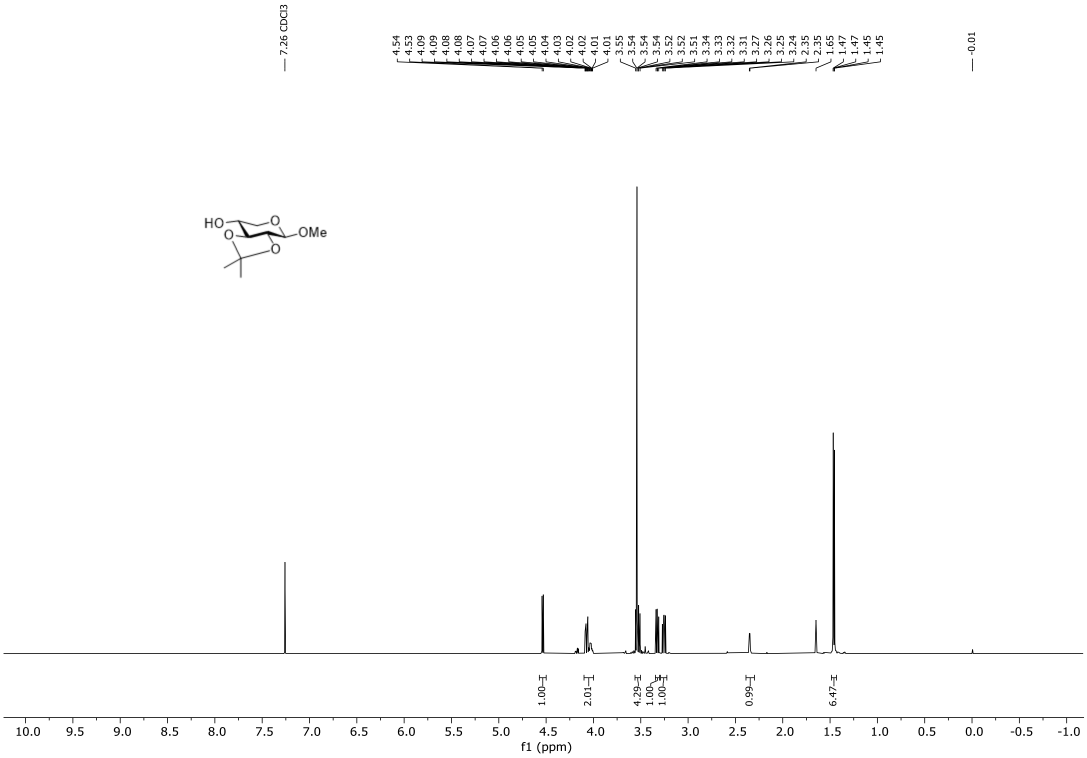

Figure S4 ^{1}H NMR spectrum of compound **S2** (600 MHz, $CDCl_3$, 298 K).

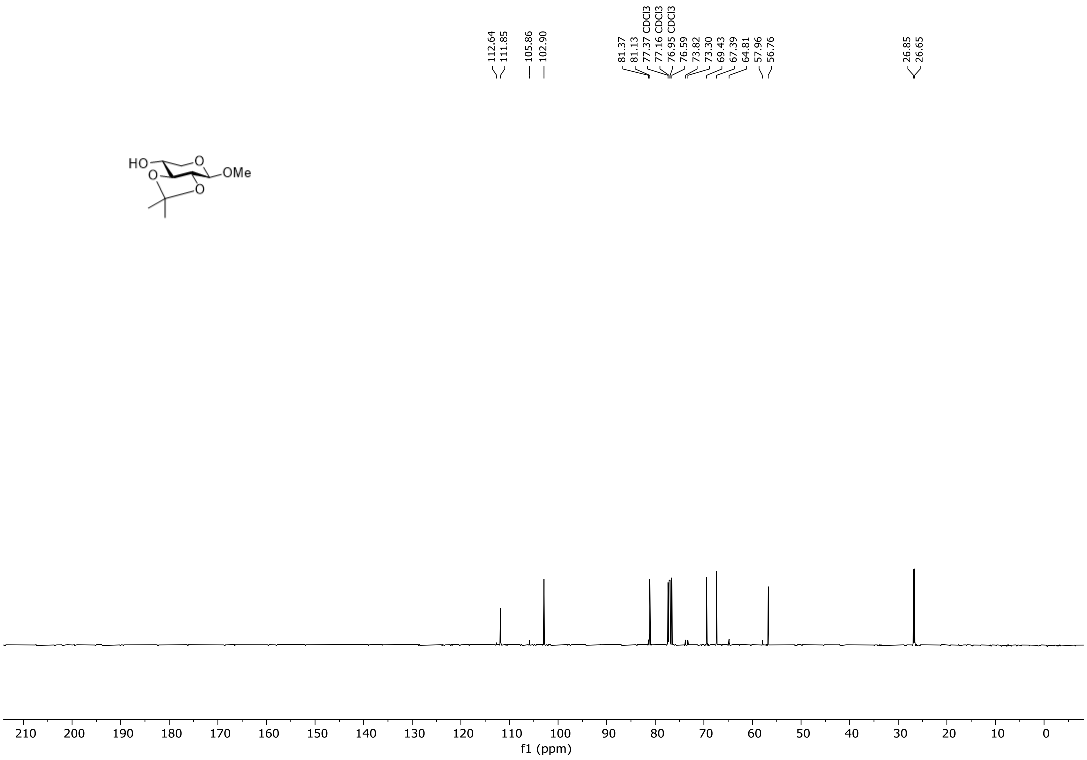

Figure S5 ^{13}C NMR spectrum of compound **S2** (151 MHz, $CDCl_3$, 298 K).

4.2 Compound S4

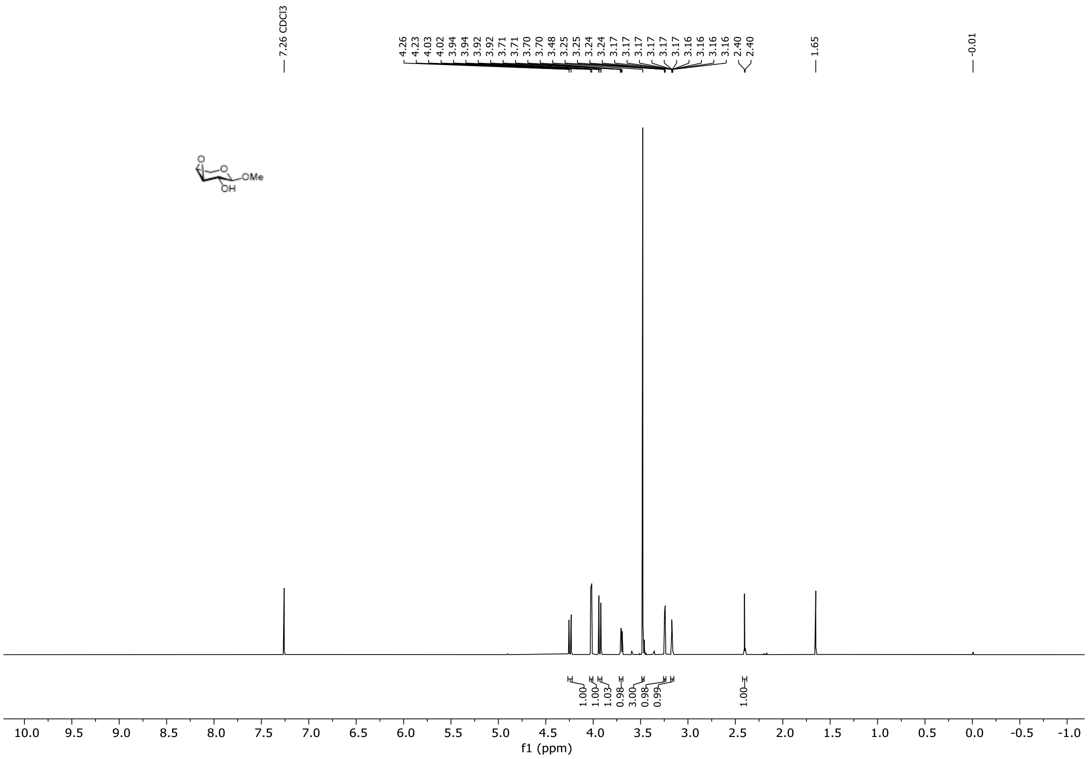

Figure S6 ^{1}H NMR spectrum of compound **S4** (600 MHz, $CDCl_3$, 298 K).

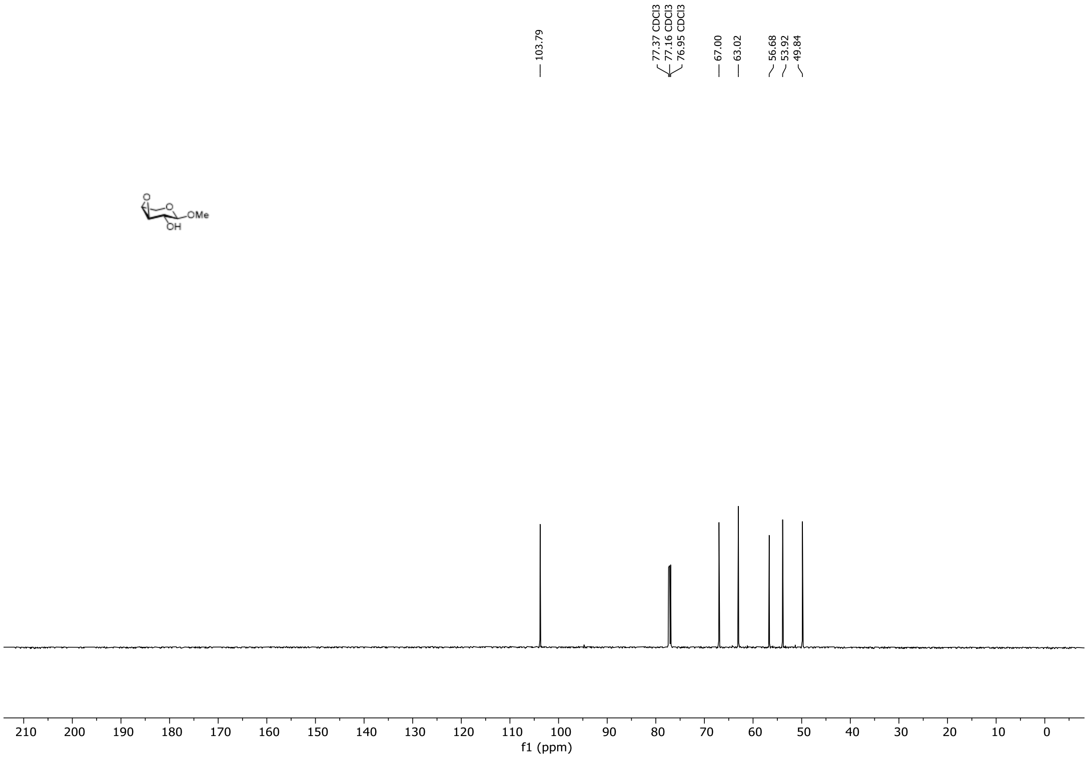

Figure S7 ^{13}C NMR spectrum of compound **S4** (151 MHz, $CDCl_3$, 298 K).

4.3 Compound S5

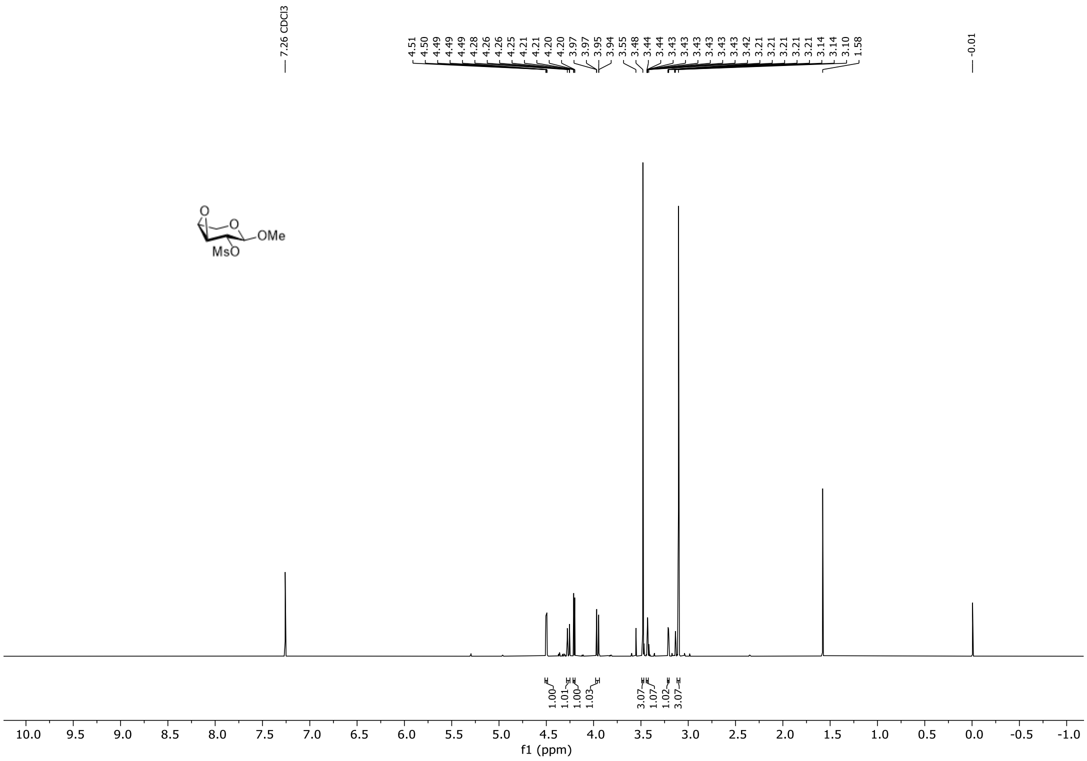

Figure S8 ^{1}H NMR spectrum of compound **S5** (600 MHz, $CDCl_3$, 298 K).

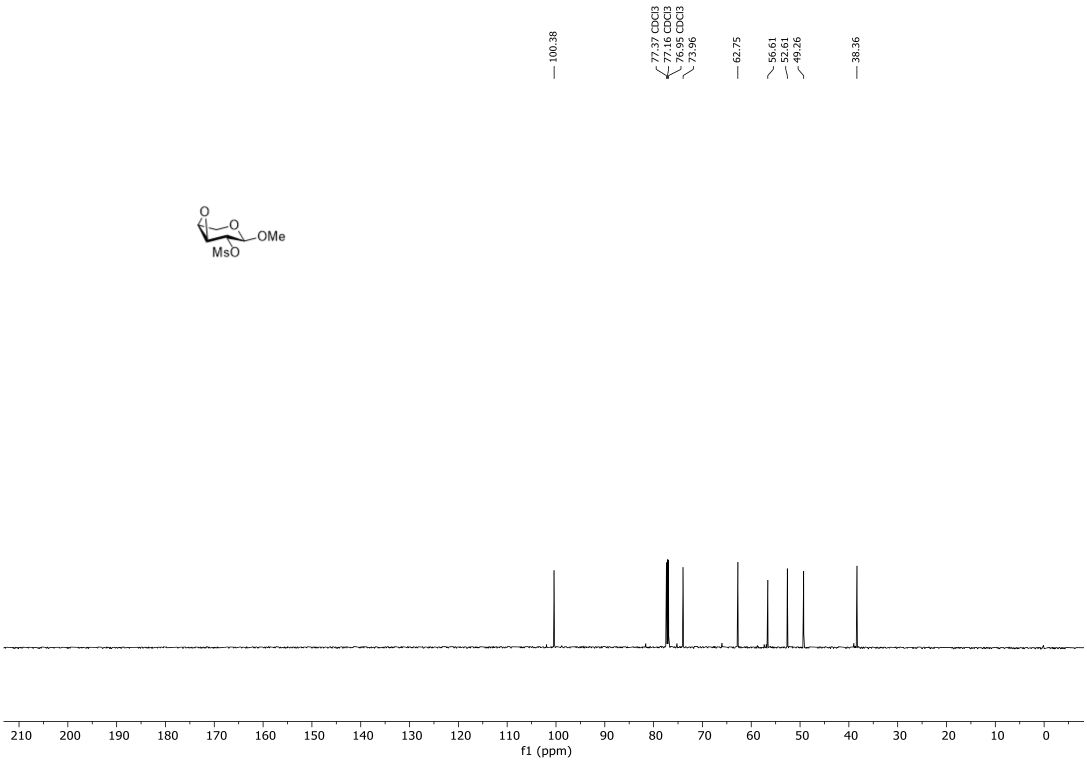

Figure S9 ^{13}C NMR spectrum of compound **S5** (151 MHz, $CDCl_3$, 298 K).

4.4 Compound S6

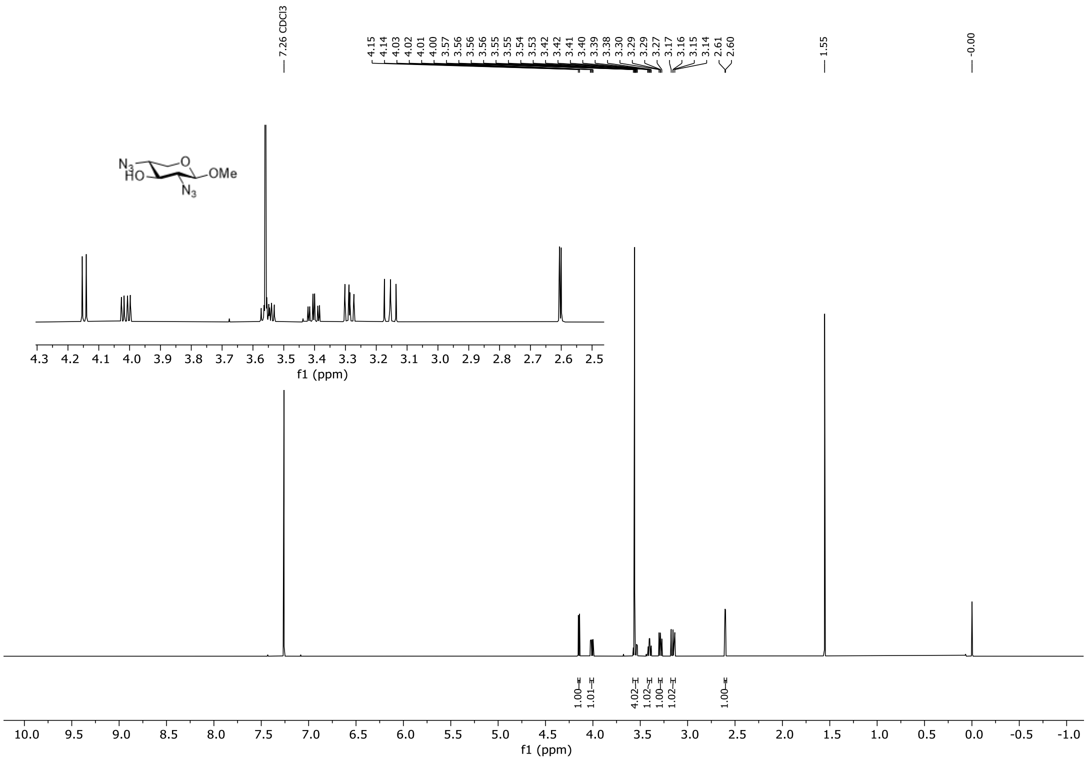

Figure S10 ^{1}H NMR spectrum of compound **S6** (600 MHz, $CDCl_3$, 298 K).

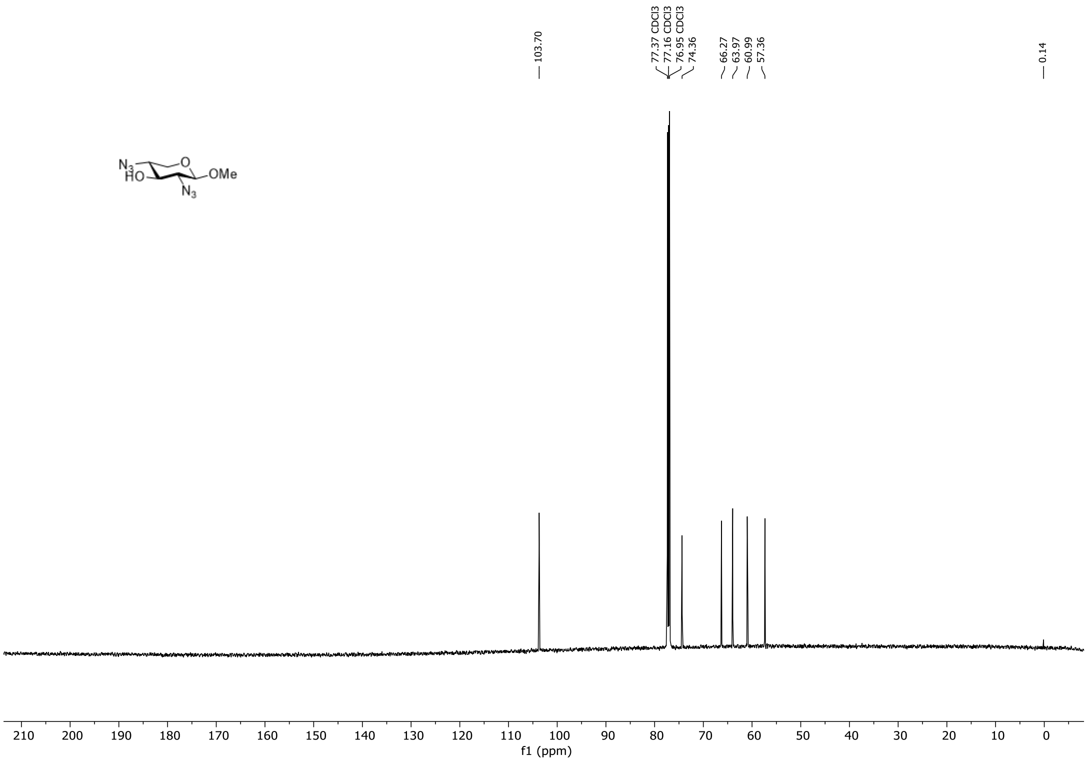

Figure S11 ^{13}C NMR spectrum of compound **S6** (151 MHz, $CDCl_3$, 298 K).

4.5 Compound S7

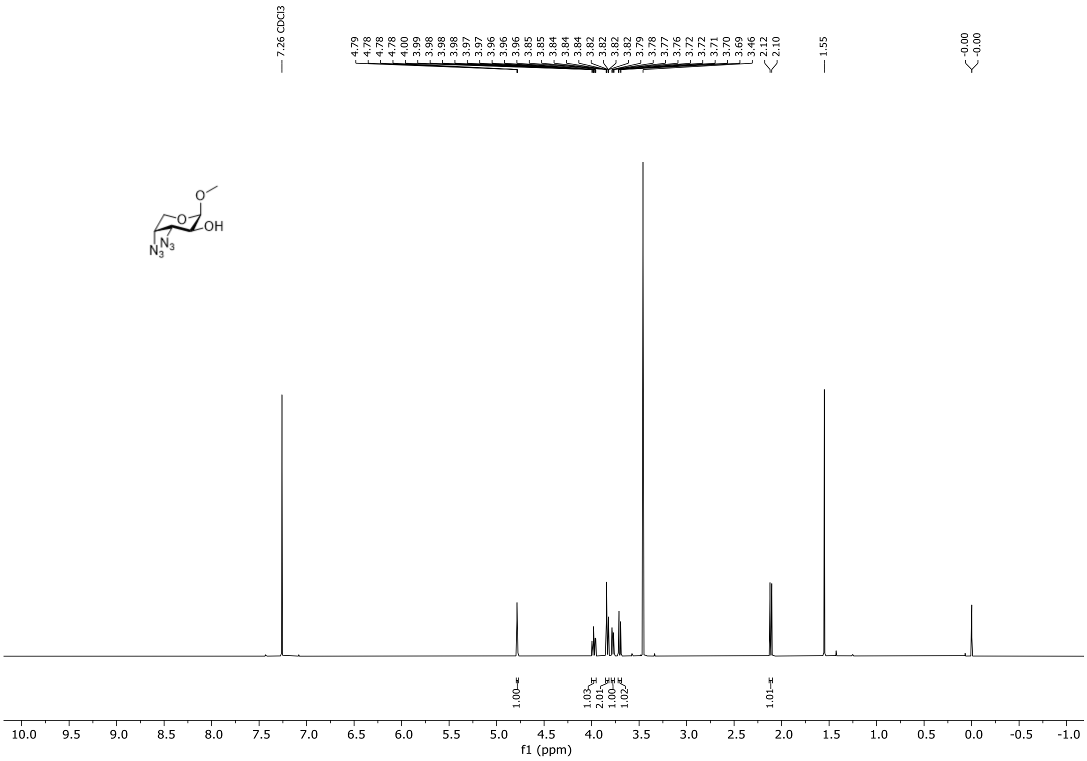

Figure S12 ^{1}H NMR spectrum of compound **S7** (600 MHz, $CDCl_3$, 298 K).

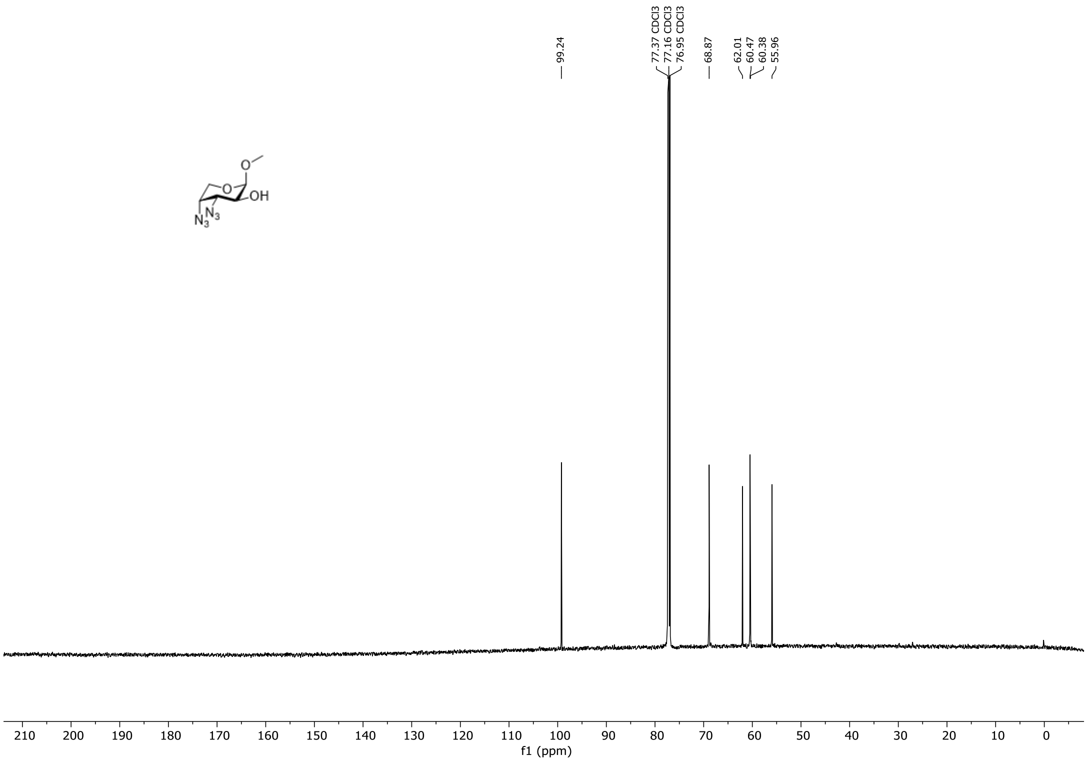

Figure S13 ^{13}C NMR spectrum of compound **S7** (151 MHz, $CDCl_3$, 298 K).

4.6 Compound 2

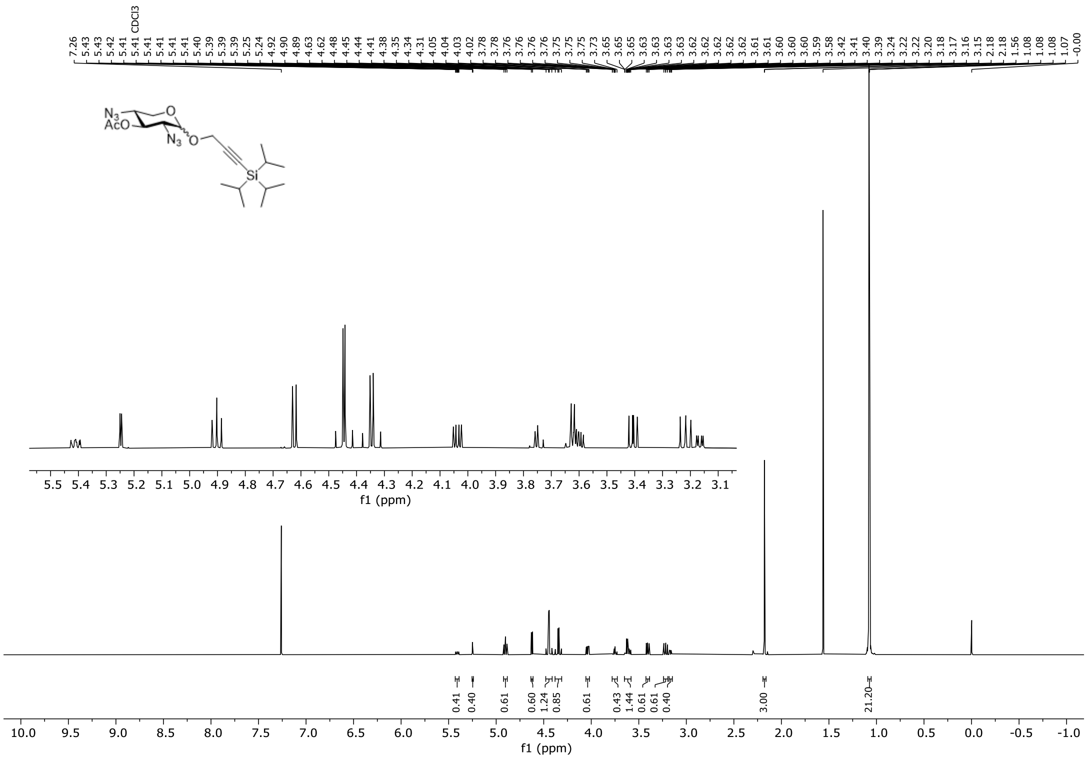

Figure S14 ^{1}H NMR spectrum of compound **2** (600 MHz, $CDCl_3$, 298 K).

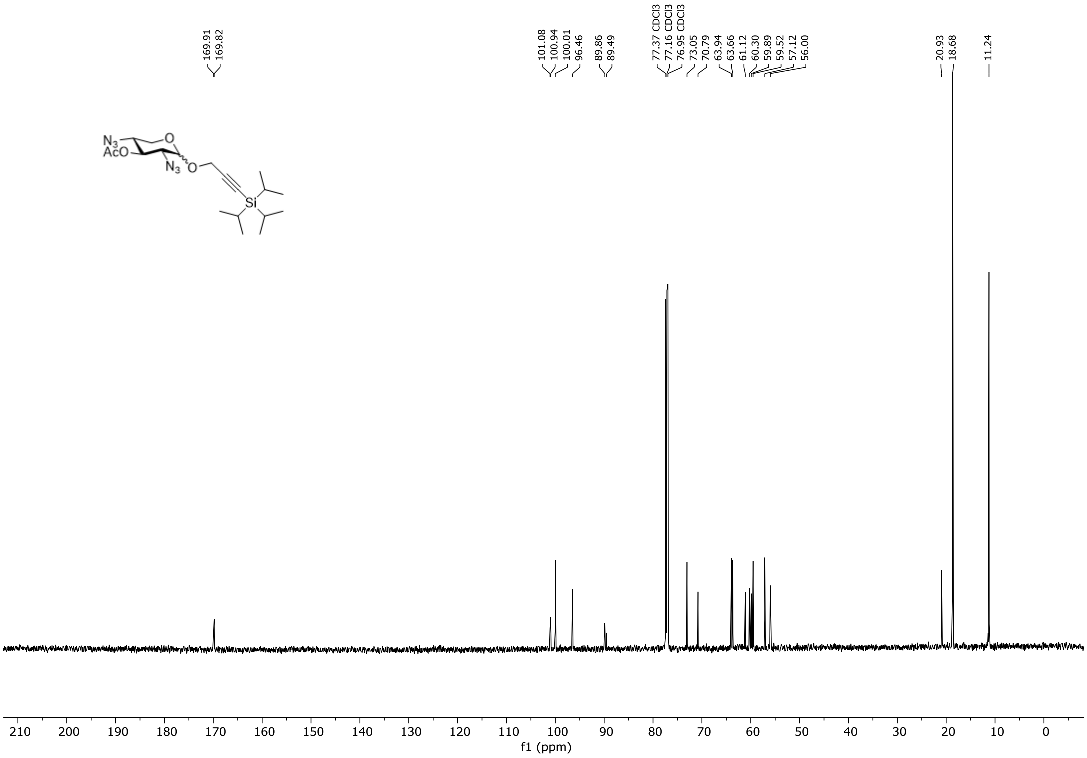

Figure S15 ^{13}C NMR spectrum of compound **2** (151 MHz, $CDCl_3$, 298 K).

4.7 Compound 3

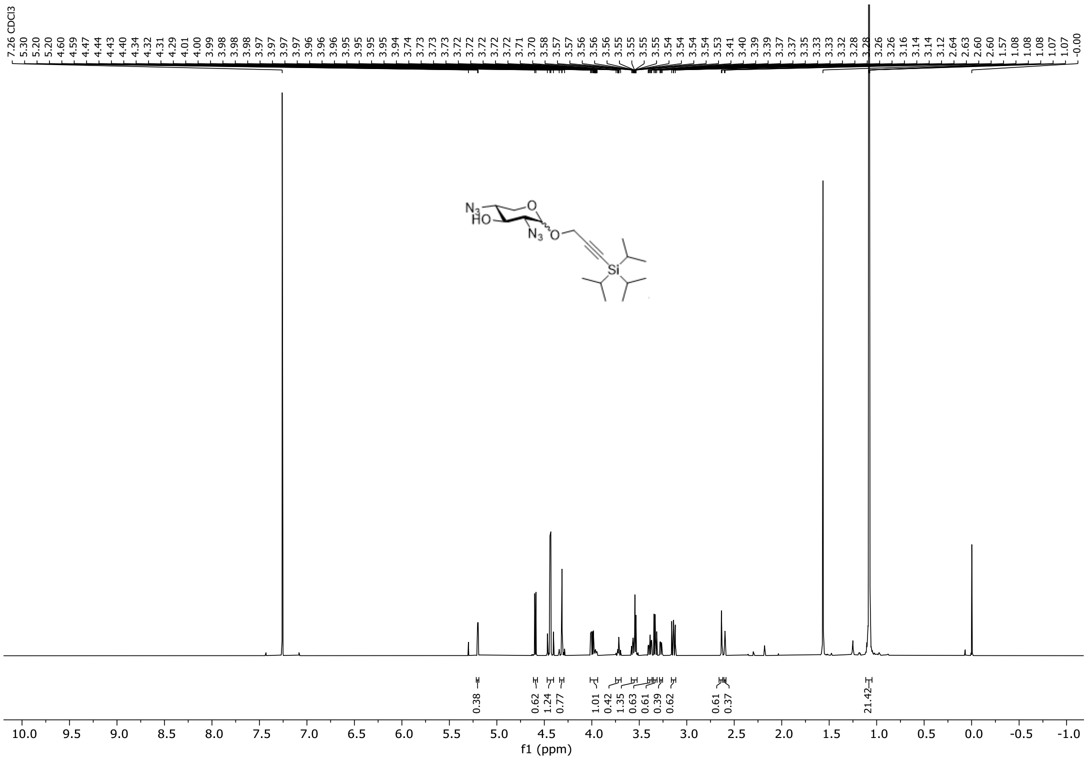

Figure S16 ^{1}H NMR spectrum of compound **3** (600 MHz, $CDCl_3$, 298 K).

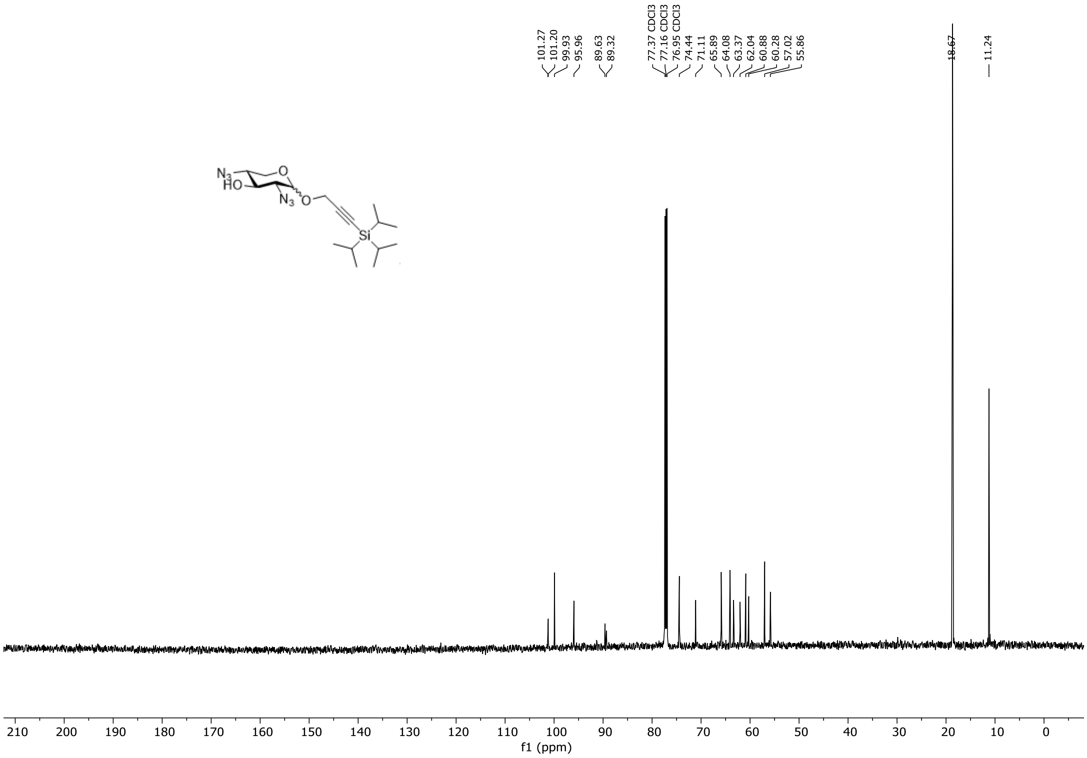

Figure S17 S23 ^{13}C NMR spectrum of compound **3** (151 MHz, $CDCl_3$, 298 K).

4.8 Compound 4

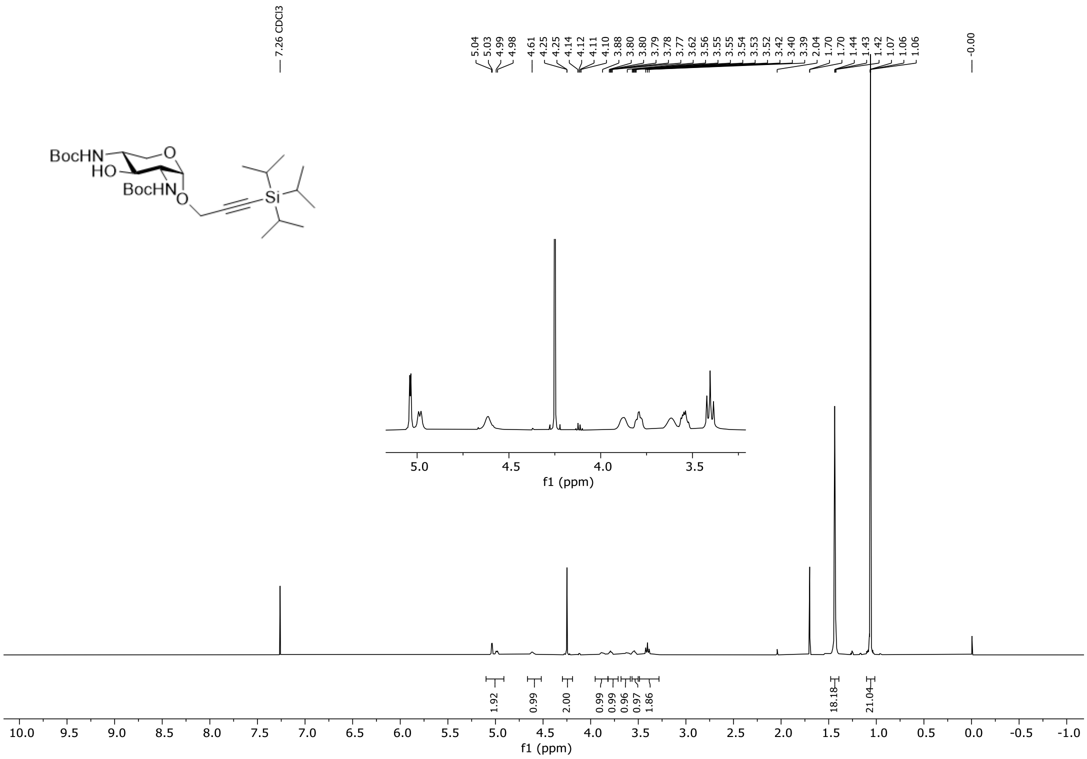

Figure S18 ^{1}H NMR spectrum of compound **4** (600 MHz, $CDCl_3$, 298 K).

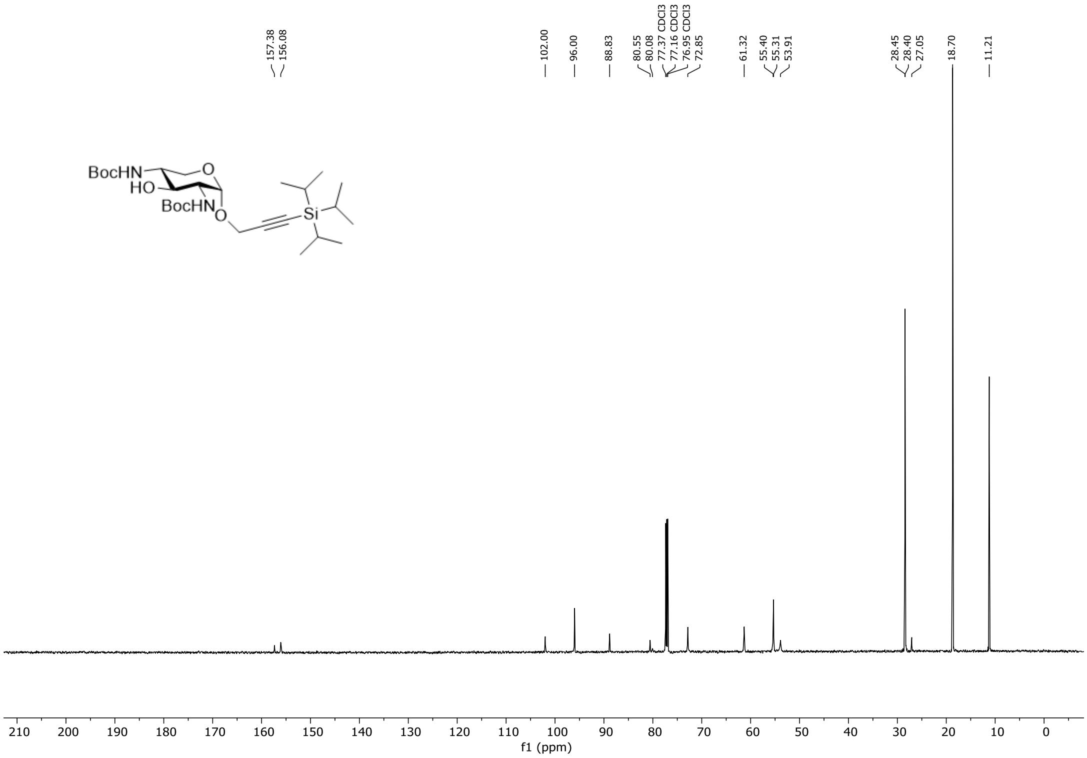

Figure S19 ^{13}C NMR spectrum of compound **4** (151 MHz, $CDCl_3$, 298 K).

4.9 Compound 5

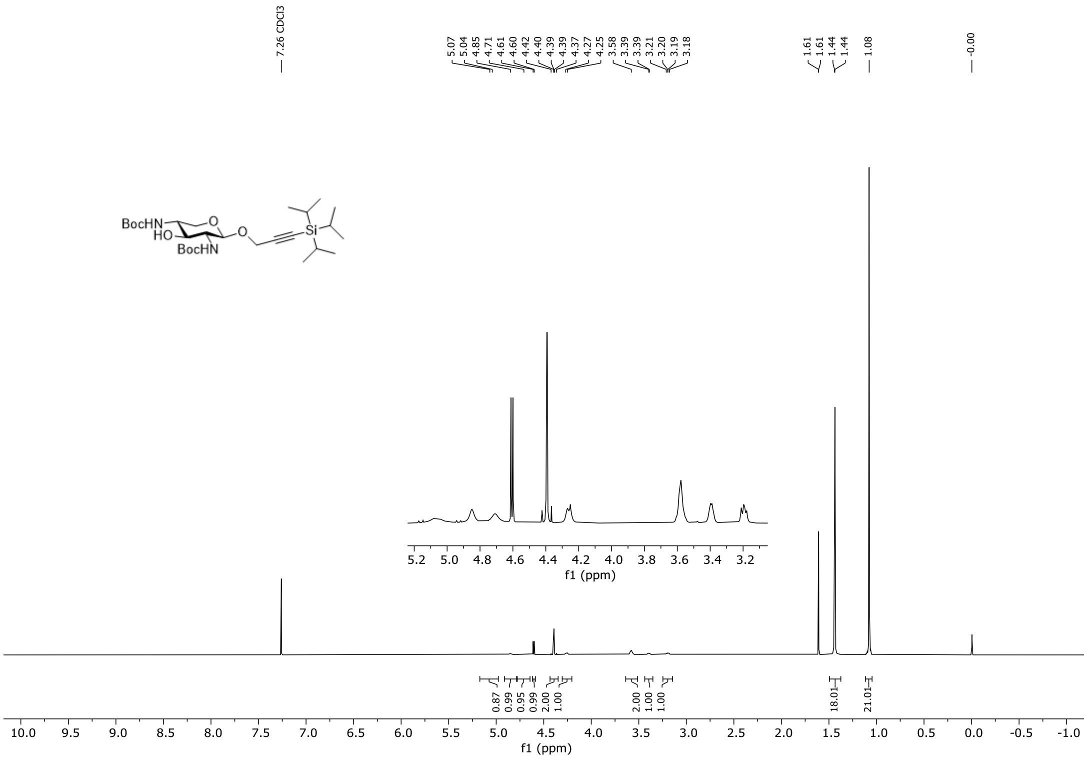

Figure S20 ¹H NMR spectrum of compound **5** (600 MHz, $CDCl_3$, 298 K).

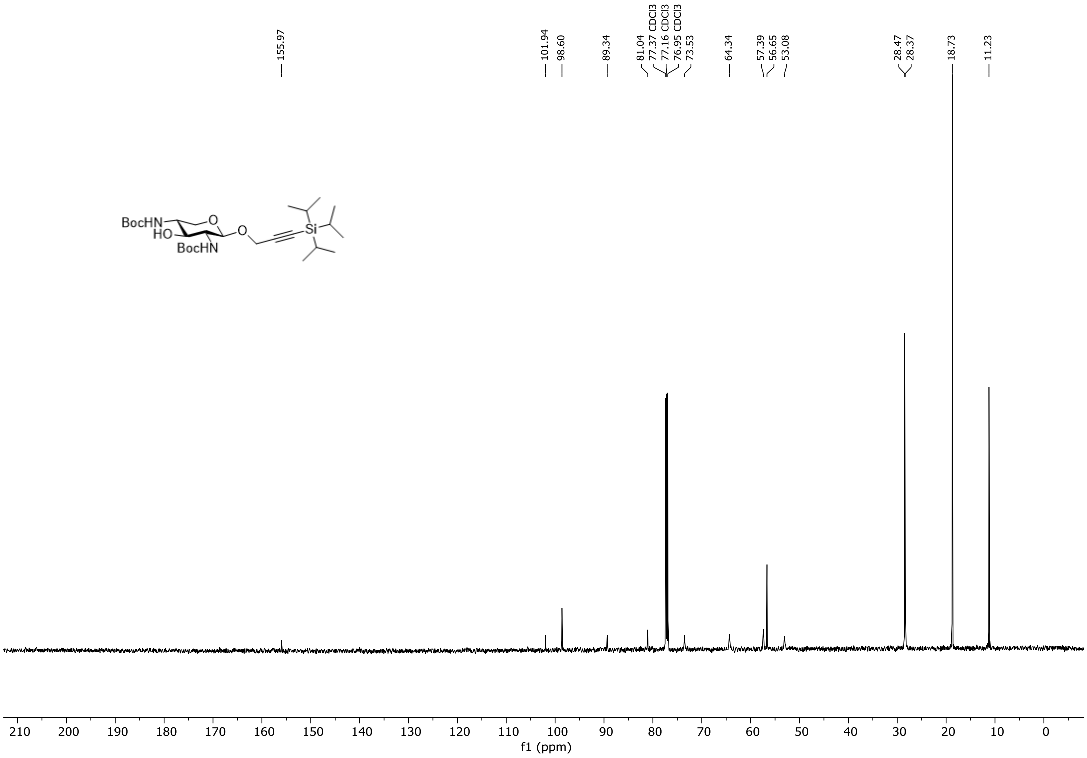

Figure S21 ¹³C NMR spectrum of compound **5** (151 MHz, $CDCl_3$, 298 K).

4.10 Compound 6

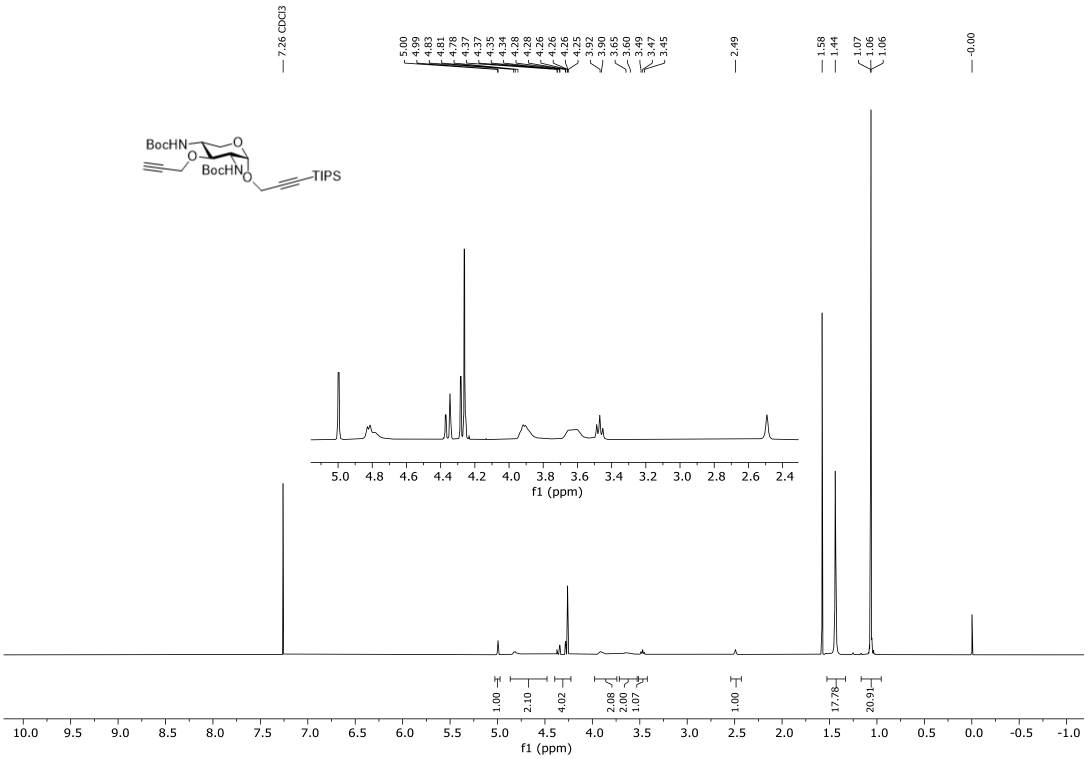

Figure S22 ^{1}H NMR spectrum of compound **6** (600 MHz, $CDCl_3$, 298 K).

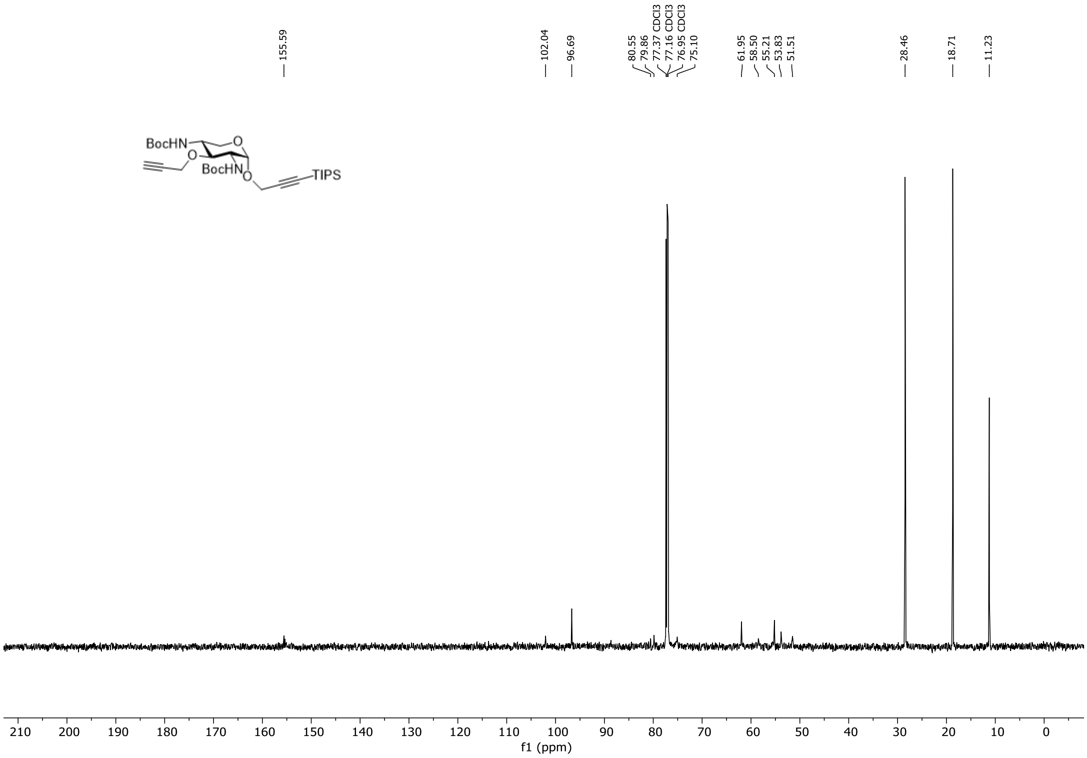

Figure S23 ^{13}C NMR spectrum of compound **6** (151 MHz, $CDCl_3$, 298 K).

4.11 Compound 7

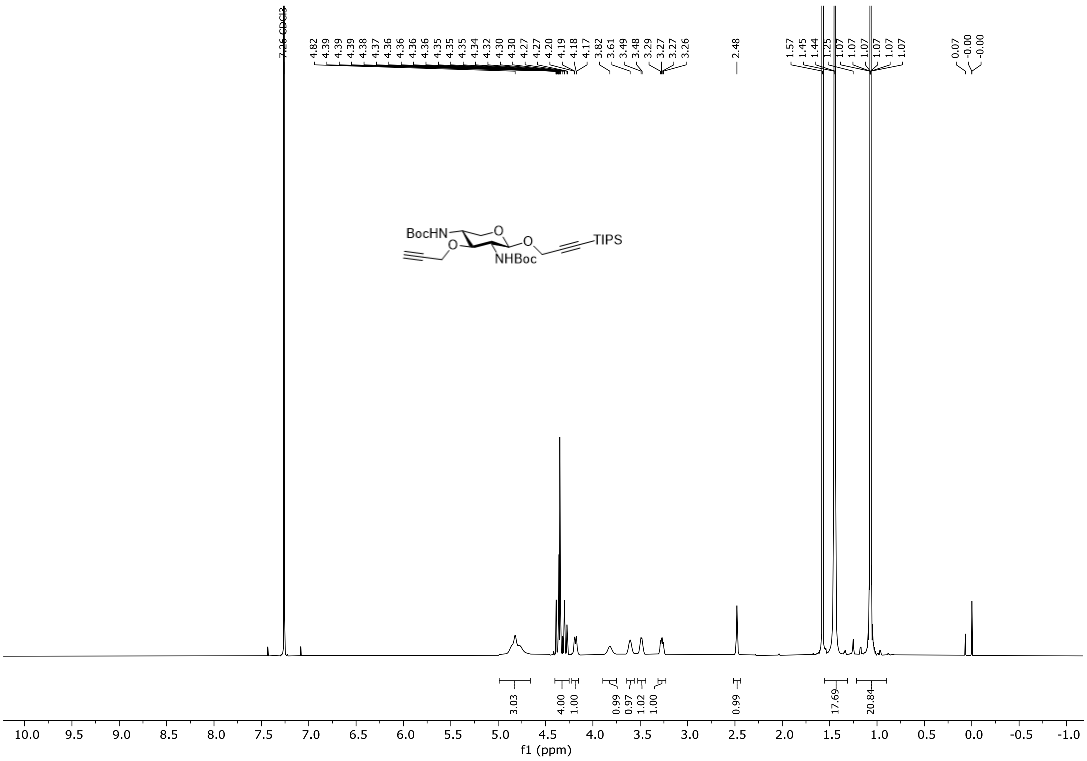

Figure S24 ^{1}H NMR spectrum of compound **7** (600 MHz, $CDCl_3$, 298 K).

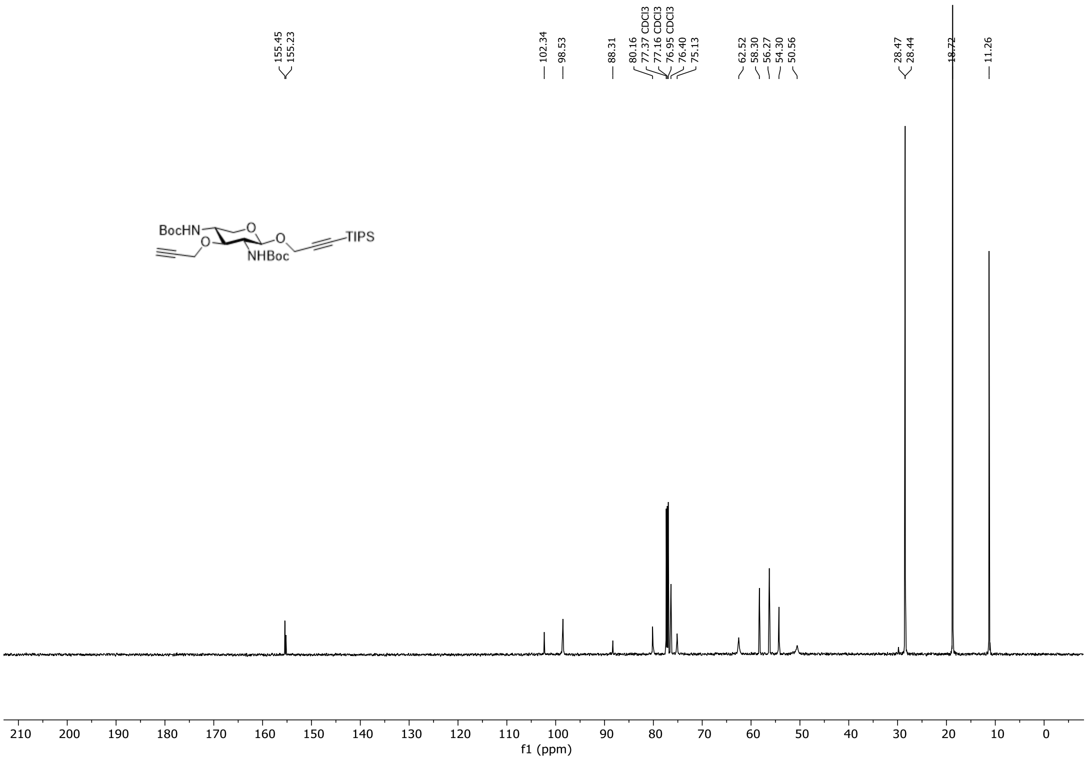

Figure S25 ^{13}C NMR spectrum of compound **7** (151 MHz, $CDCl_3$, 298 K).

4.12 Compound 8

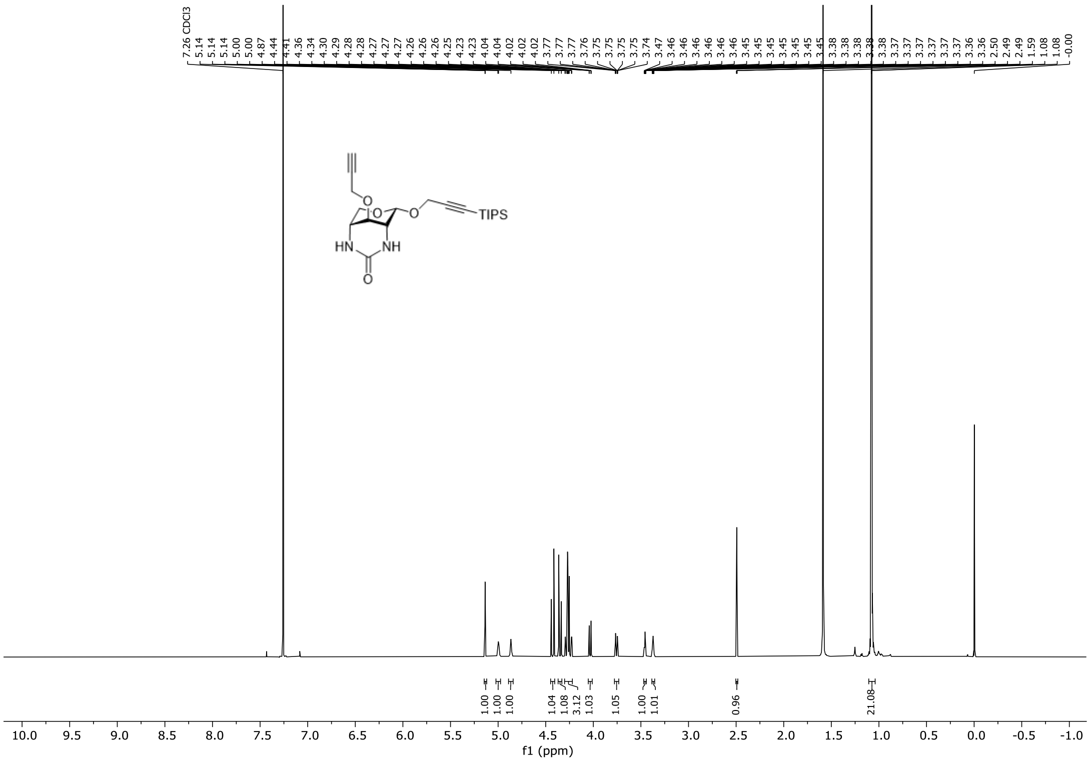

Figure S26 ^{1}H NMR spectrum of compound **8** (600 MHz, $CDCl_3$, 298 K).

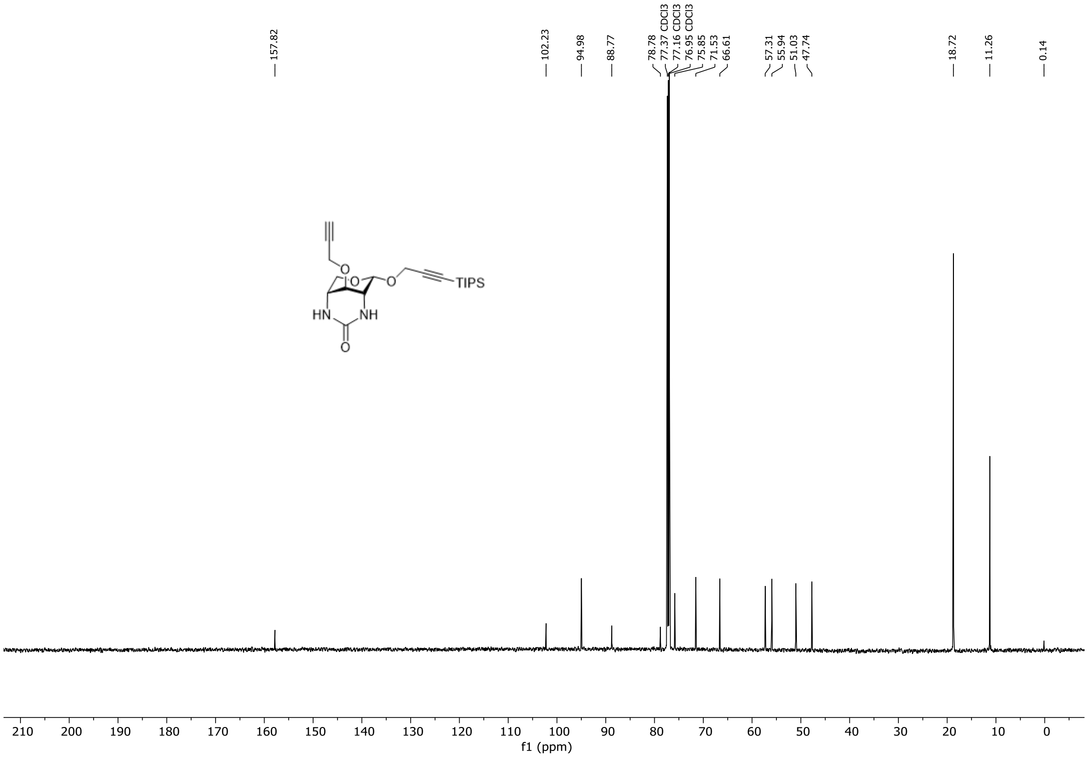

Figure S27 ^{13}C NMR spectrum of compound **8** (151 MHz, $CDCl_3$, 298 K).

4.13 Compound 9

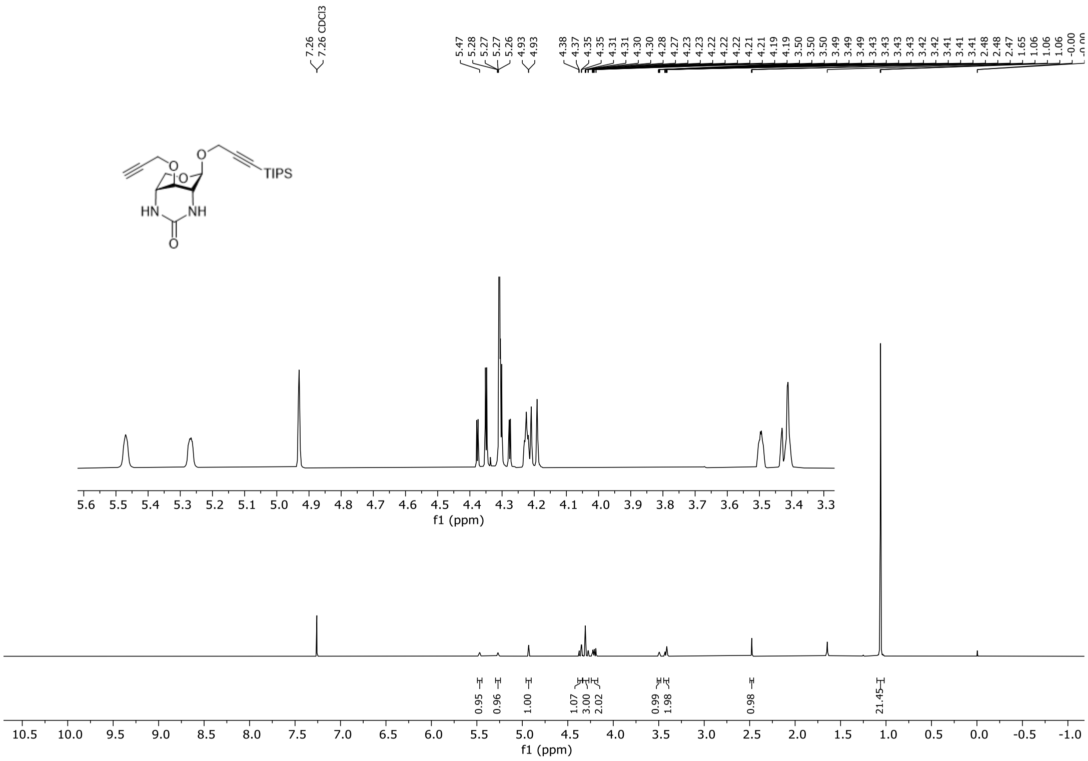

Figure S28 ^{1}H NMR spectrum of compound **9** (600 MHz, $CDCl_3$, 298 K).

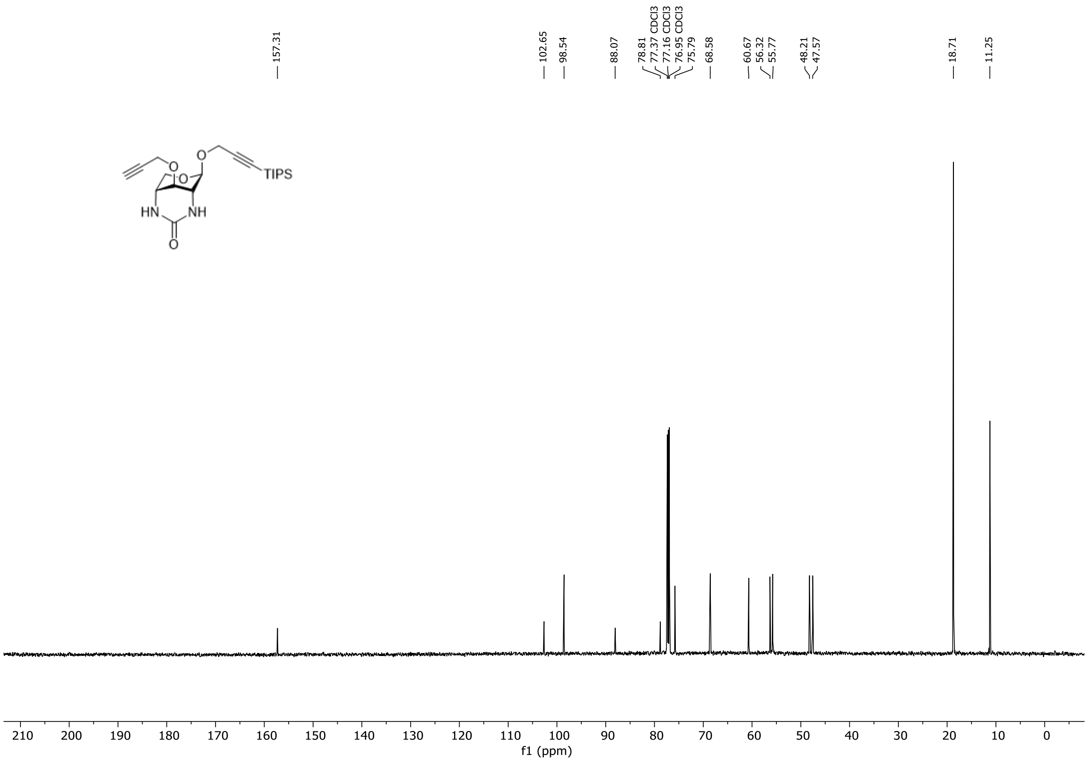

Figure S29 ^{13}C NMR spectrum of compound **9** (151 MHz, $CDCl_3$, 298 K).

4.14 Compound 10

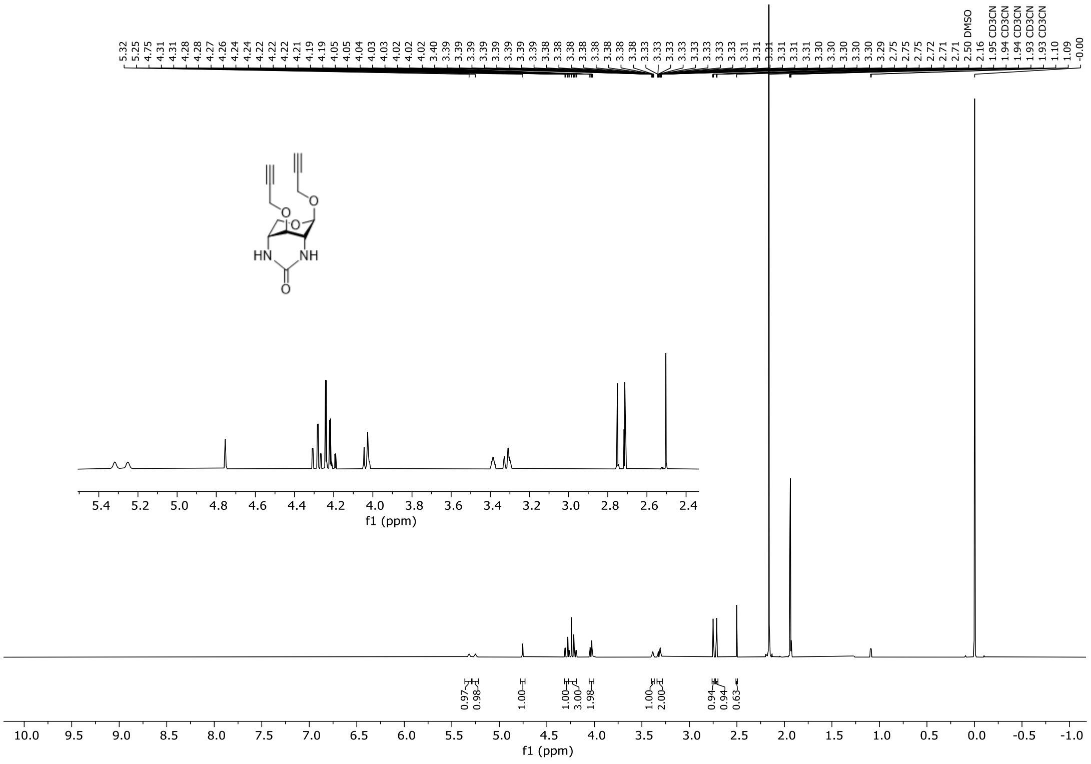

Figure S30 ^{1}H NMR spectrum of compound **10** (600 MHz, CD_3CN, 298 K).

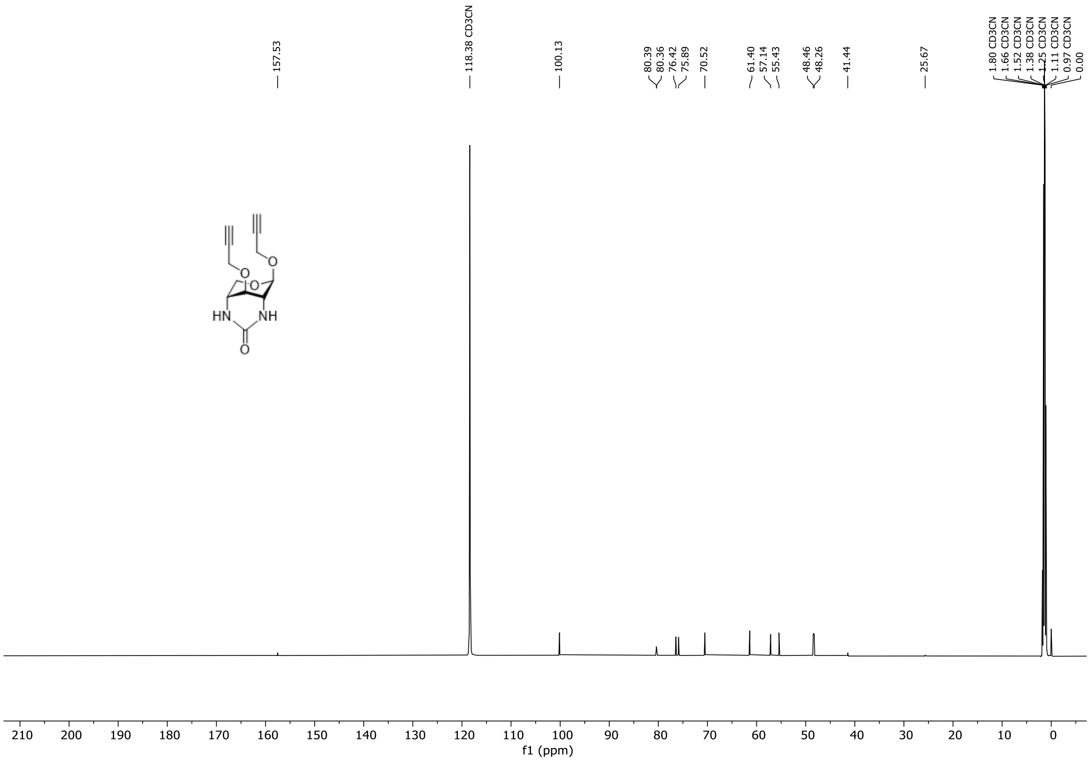

Figure S31 ^{13}C NMR spectrum of compound **10** (151 MHz, CD_3CN, 298 K).

4.15 Compound 15

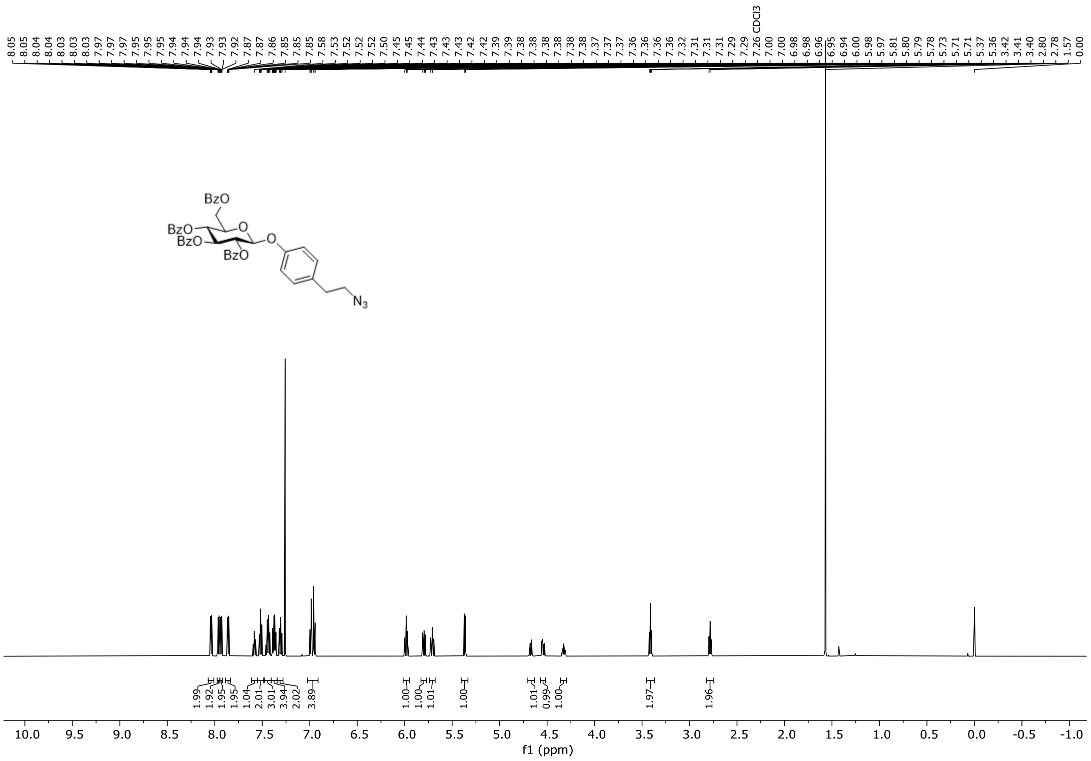

Figure S32 ^{1}H NMR spectrum of compound **15** (600 MHz, $CDCl_3$, 298 K).

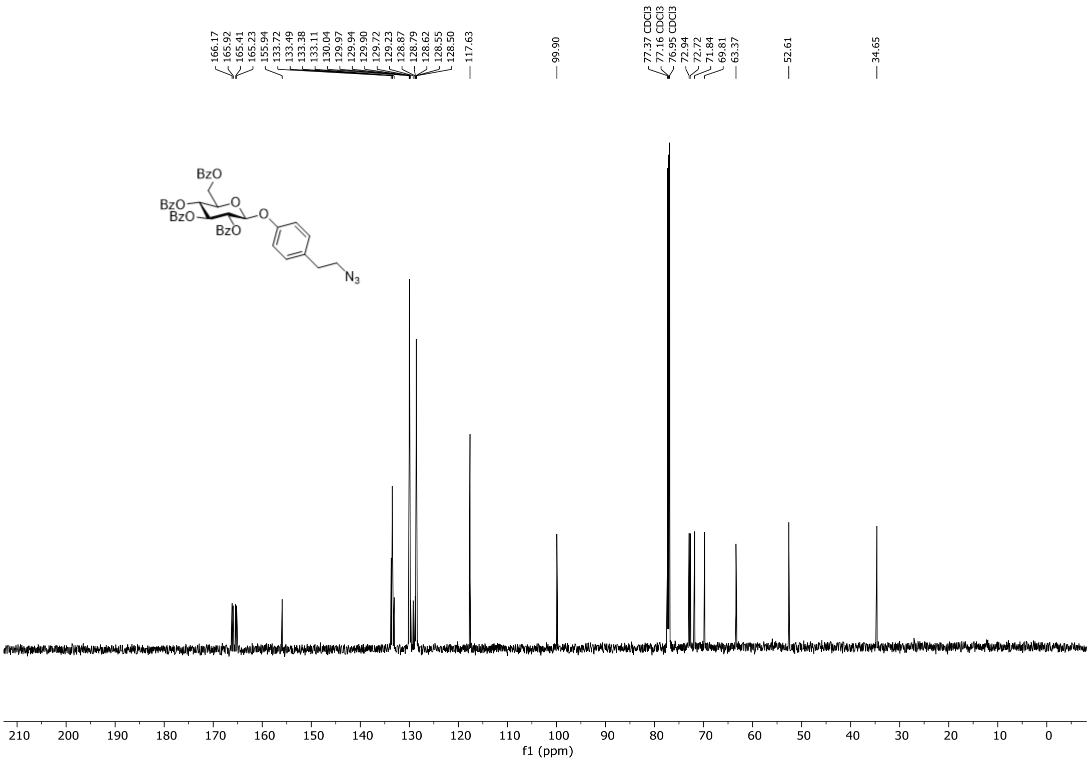

Figure S33 ^{13}C NMR spectrum of compound **15** (151 MHz, $CDCl_3$, 298 K).

4.16 Glycoconjugate 16

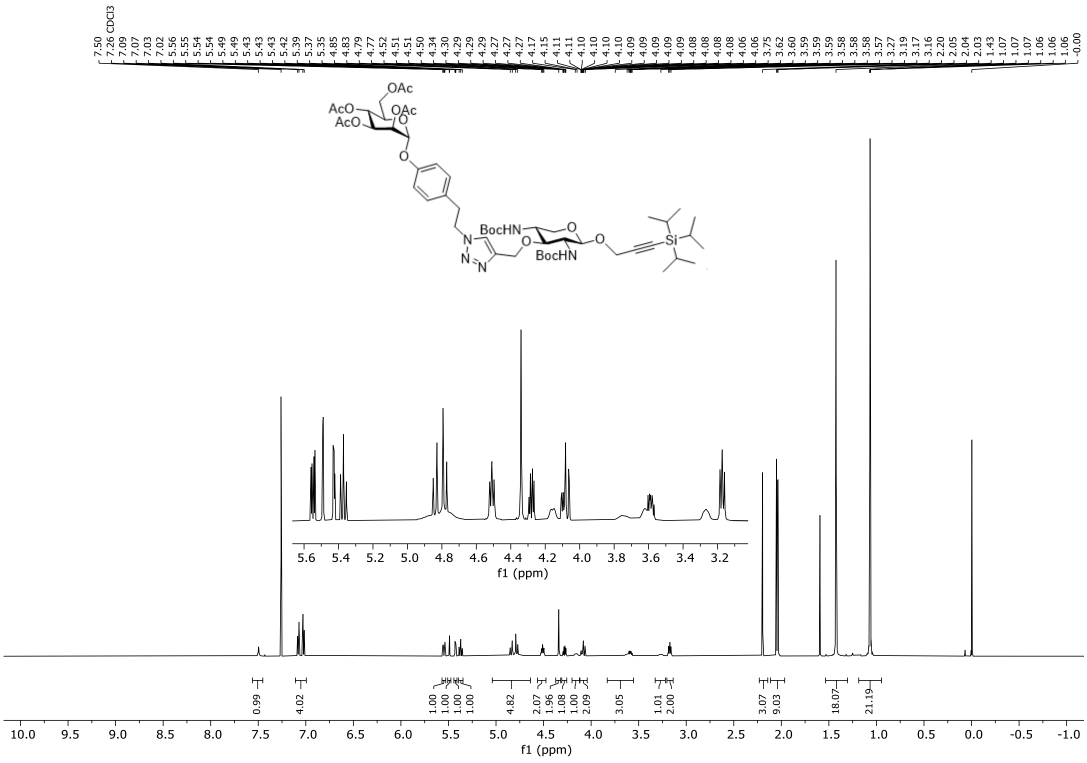

Figure S34 ^{1}H NMR spectrum of compound **16** (600 MHz, $CDCl_3$, 298 K).

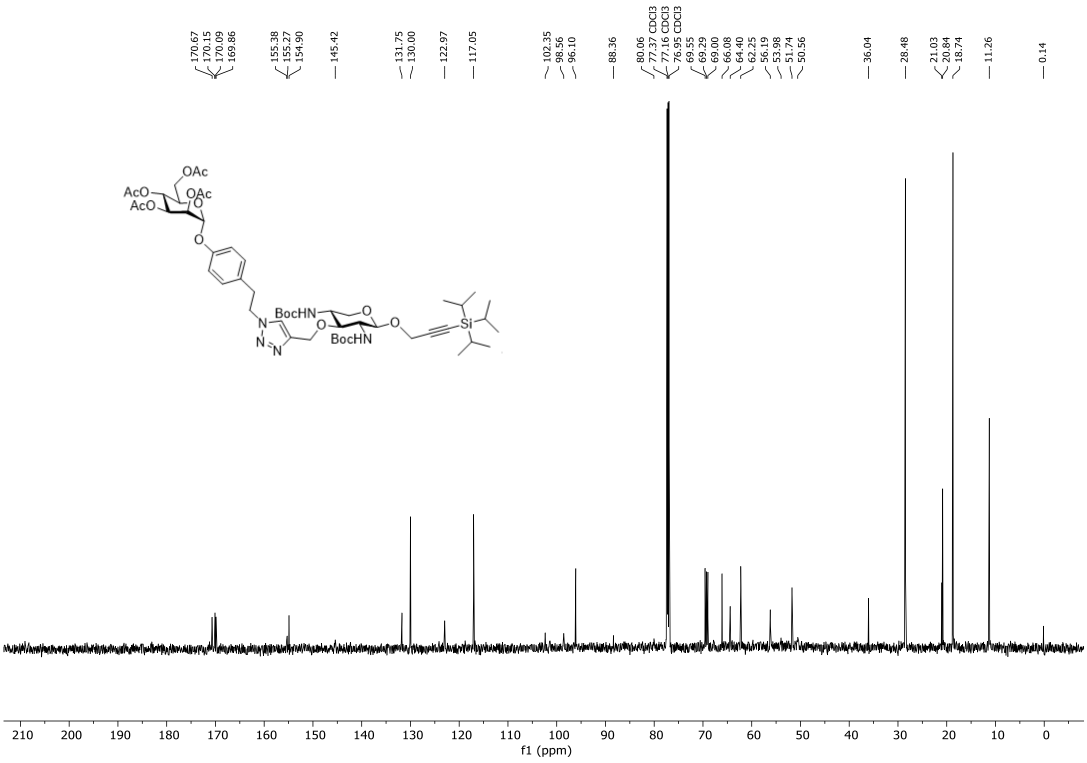

Figure S35 ^{13}C NMR spectrum of compound **16** (151 MHz, $CDCl_3$, 298 K).

4.17 Glycoconjugate 17

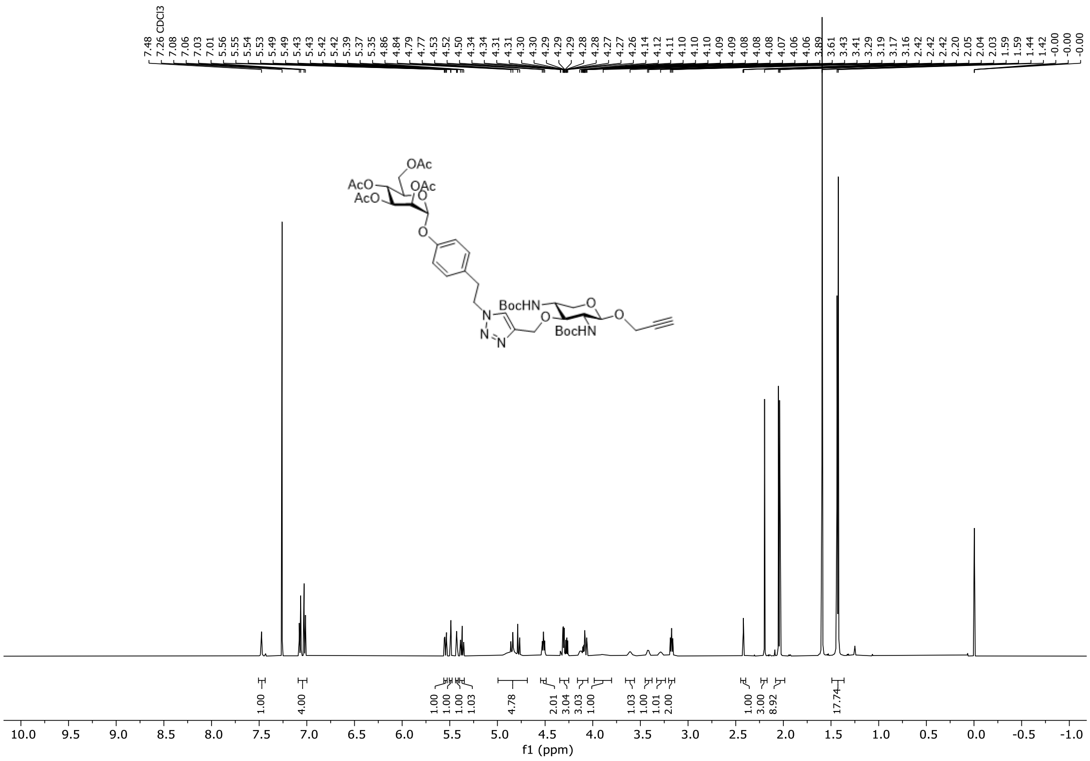

Figure S36 ^{1}H NMR spectrum of compound **17** (600 MHz, $CDCl_3$, 298 K).

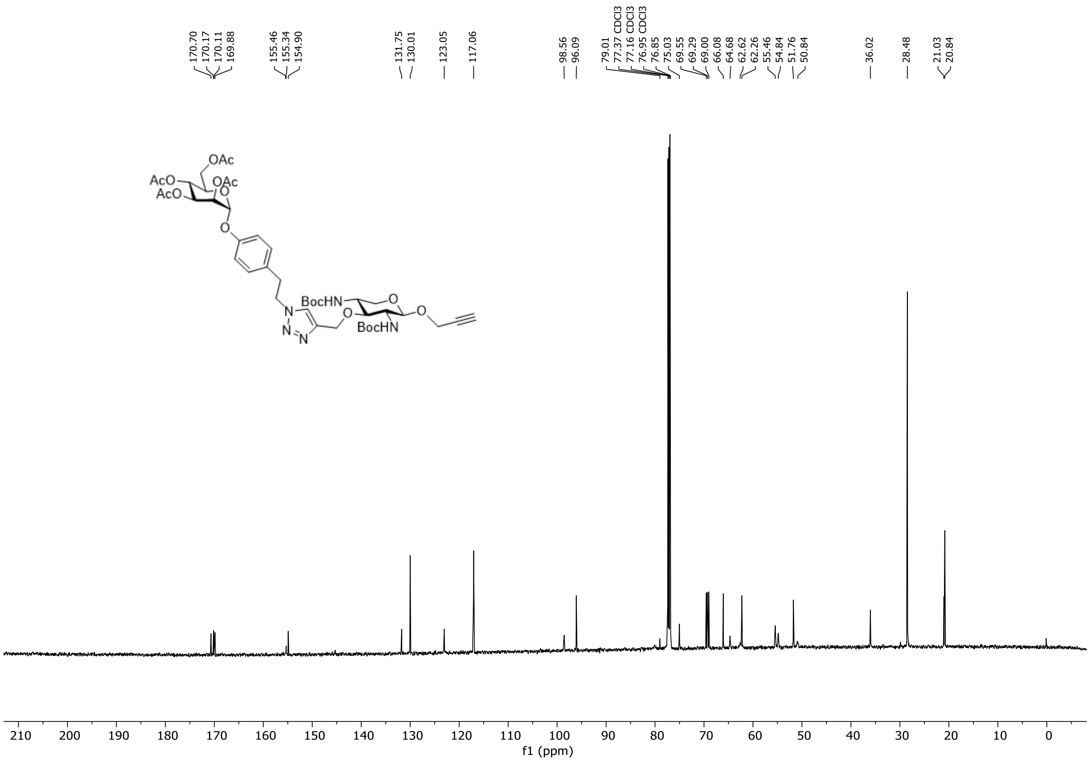

Figure S37 ^{13}C NMR spectrum of compound **17** (151 MHz, $CDCl_3$, 298 K).

4.18 Glycoconjugate 18

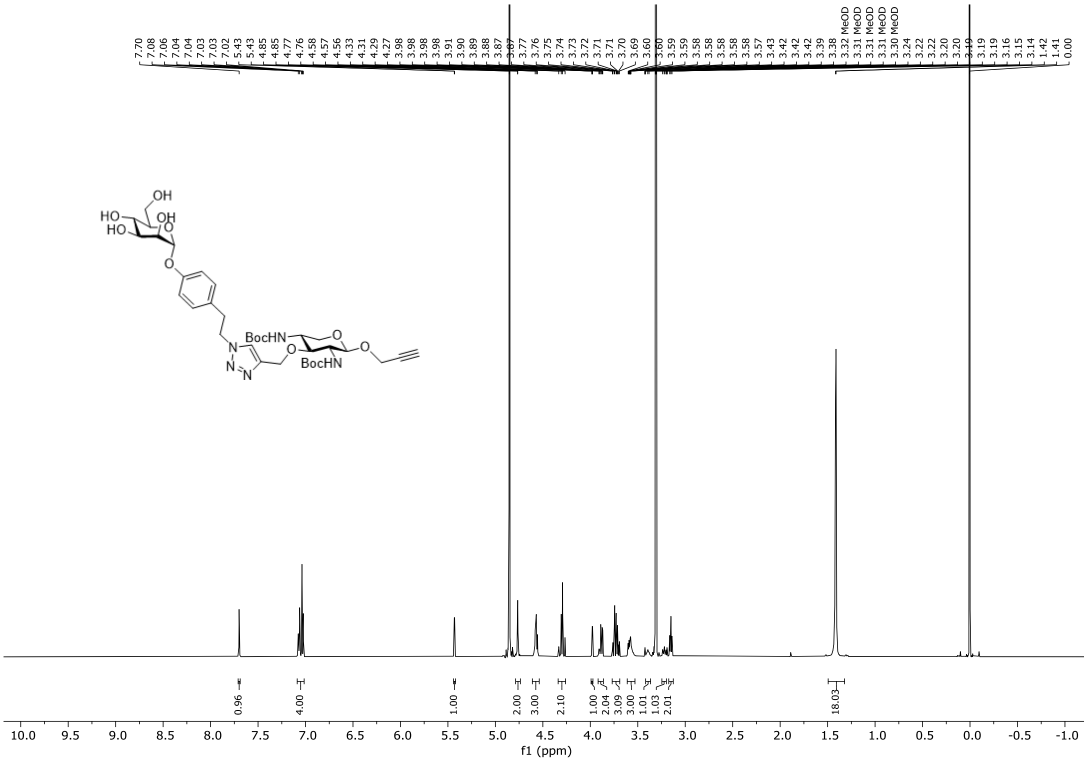

Figure S38 ^{1}H NMR spectrum of compound **18** (600 MHz, CD_3OD, 298 K).

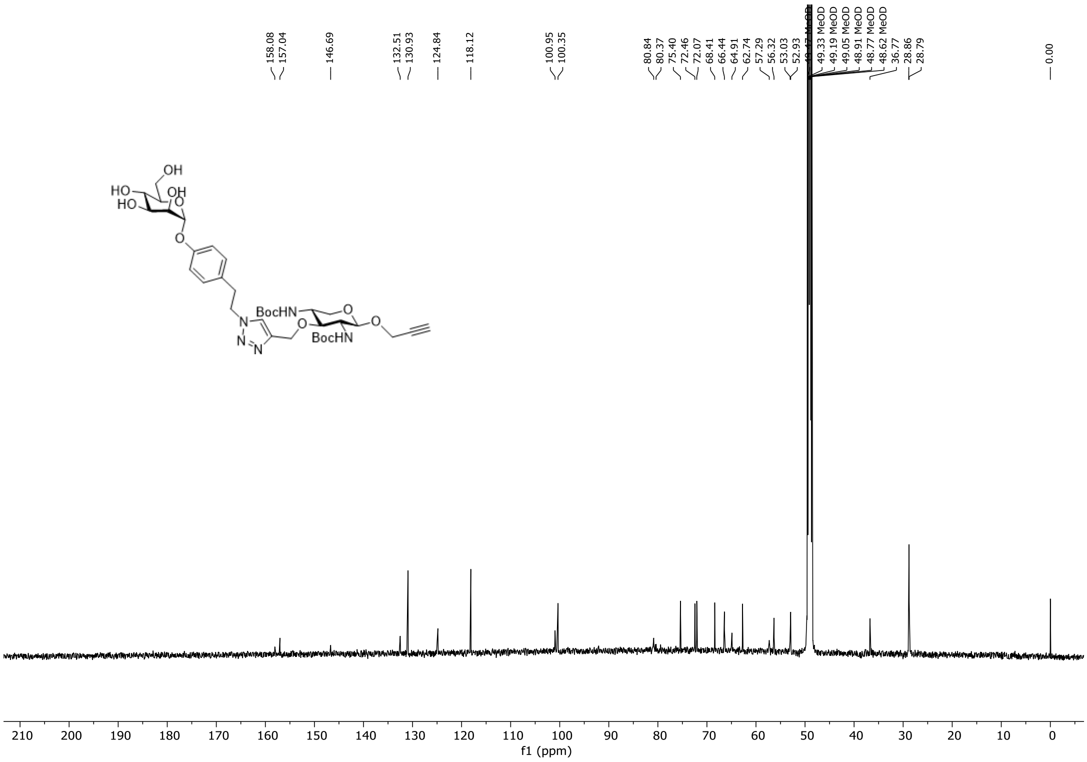

Figure S39 ^{13}C NMR spectrum of compound **18** (151 MHz, CD_3OD, 298 K).

4.19 Glycoconjugate 19

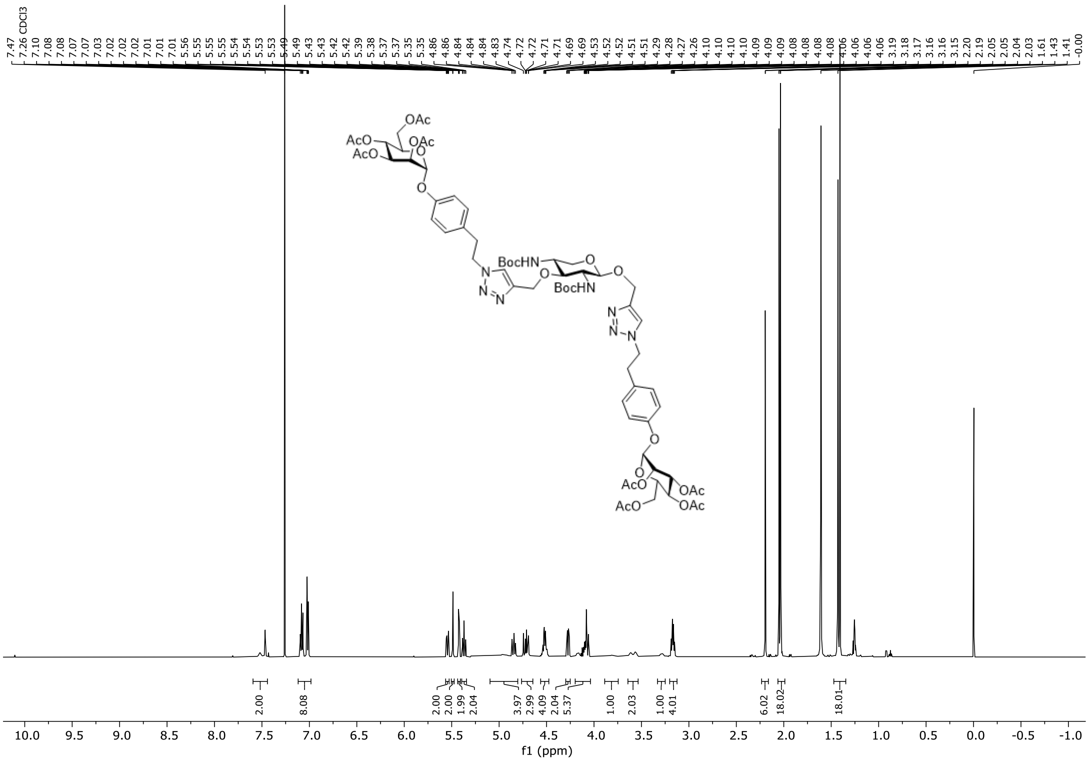

Figure S40 ^{1}H NMR spectrum of compound **19** (600 MHz, $CDCl_3$, 298 K).

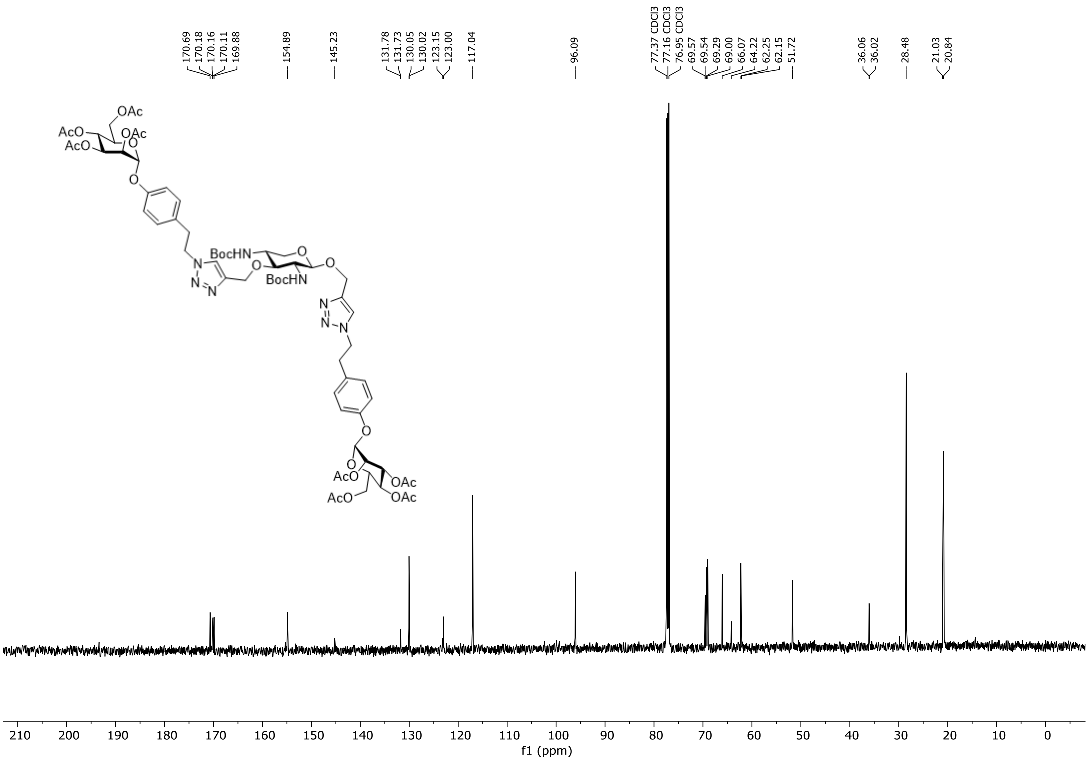

Figure S41 ^{13}C NMR spectrum of compound **19** (151 MHz, $CDCl_3$, 298 K).

4.20 Glycoconjugate 20

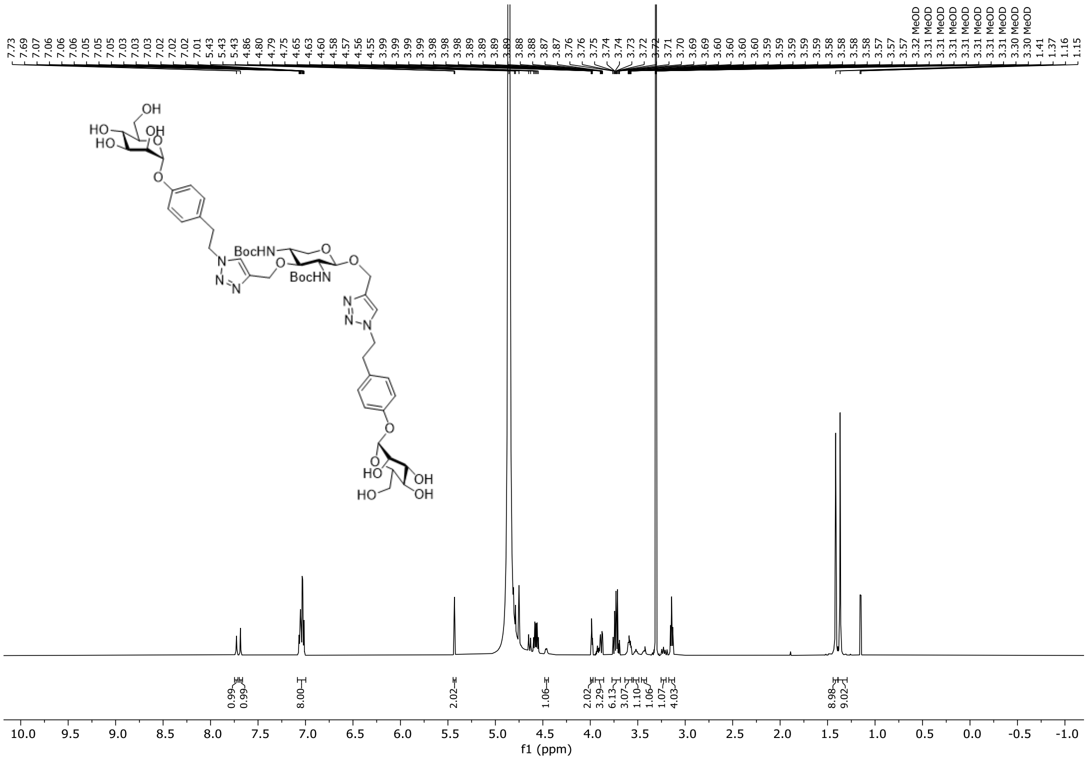

Figure S42 ^{1}H NMR spectrum of compound **20** (600 MHz, CD_3OD, 298 K).

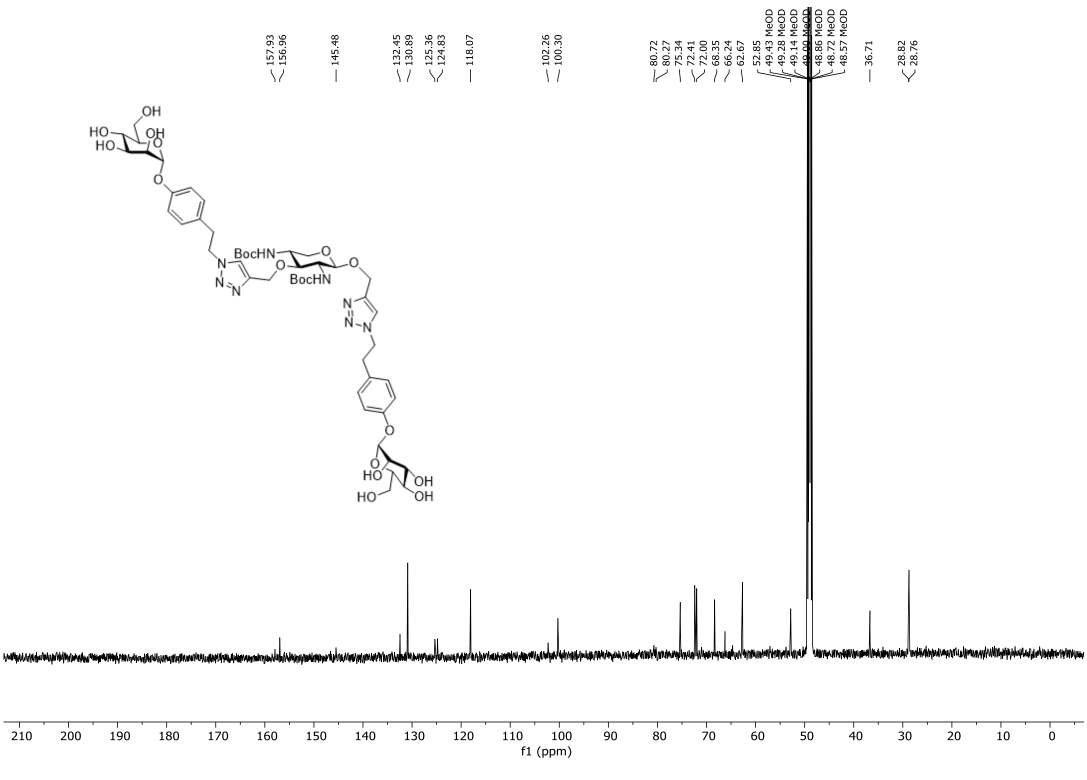

Figure S43 ^{13}C NMR spectrum of compound **20** (151 MHz, CD_3OD, 298 K).

4.21 Glycoconjugate 21

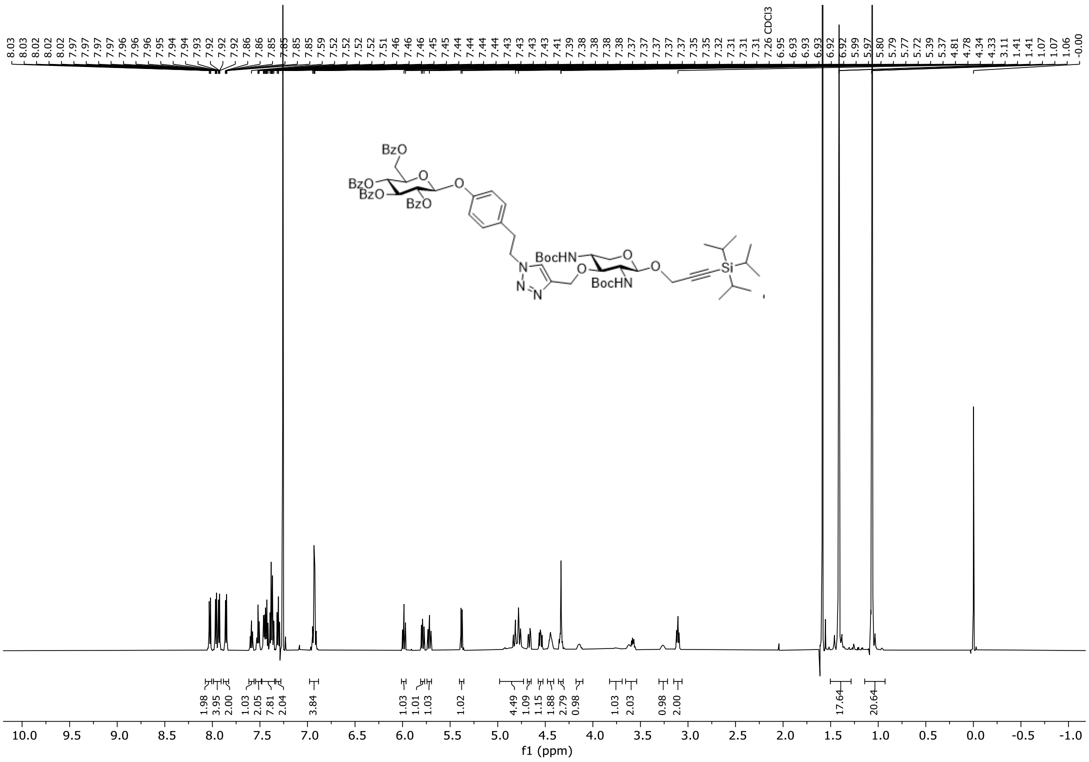

Figure S44 ^{1}H NMR spectrum of compound **21** (600 MHz, $CDCl_3$, 298 K).

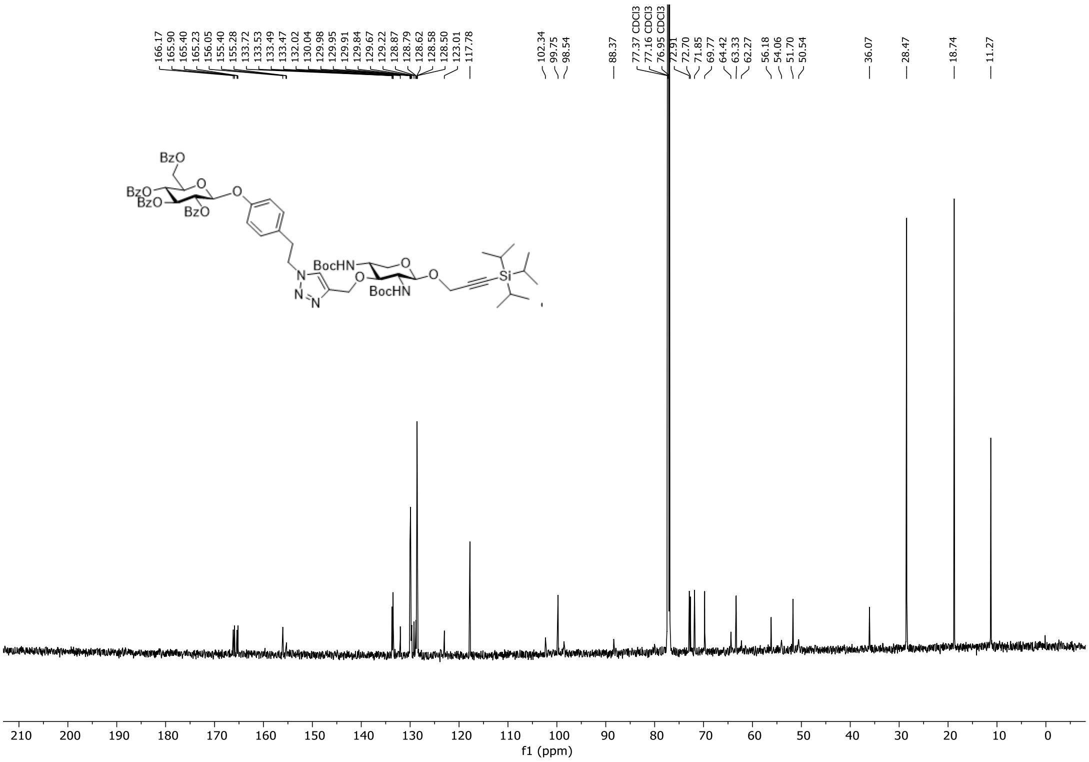

Figure S45 ^{13}C NMR spectrum of compound **21** (151 MHz, $CDCl_3$, 298 K).

4.22 Glycoconjugate 22

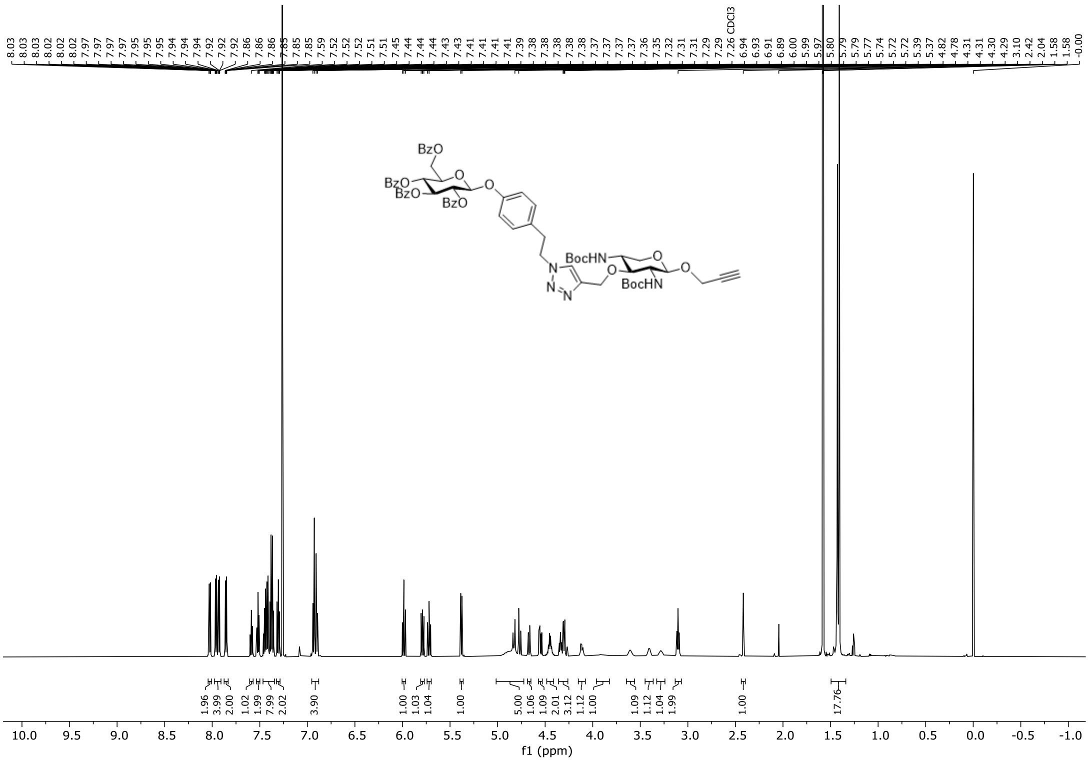

Figure S46 ^{1}H NMR spectrum of compound **22** (600 MHz, $CDCl_3$, 298 K).

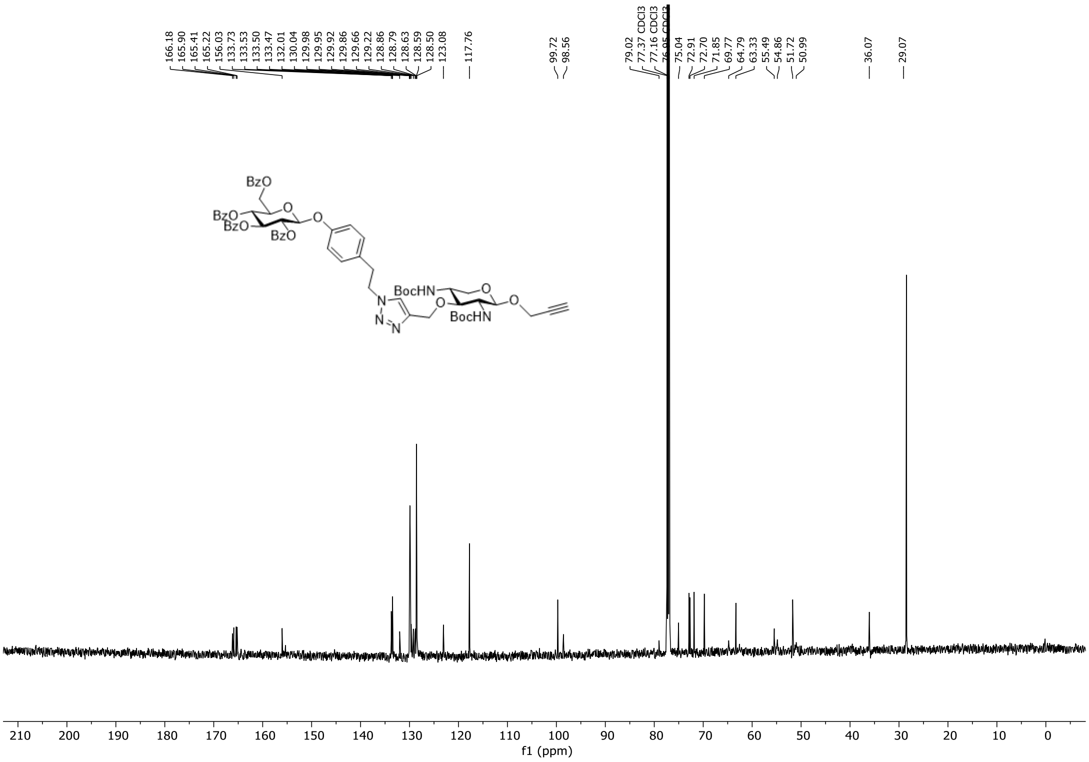

Figure S47 ^{13}C NMR spectrum of compound **22** (151 MHz, $CDCl_3$, 298 K).

4.23 Glycoconjugate 23

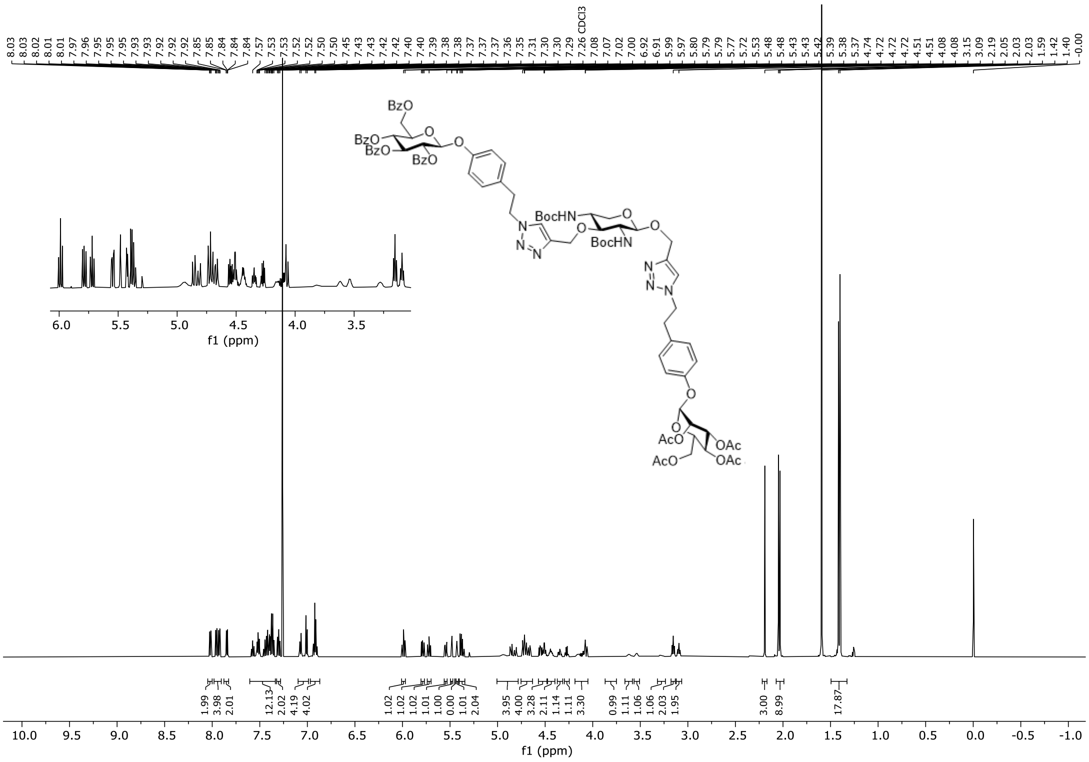

Figure S48 ^{1}H NMR spectrum of compound **23** (600 MHz, $CDCl_3$, 298 K).

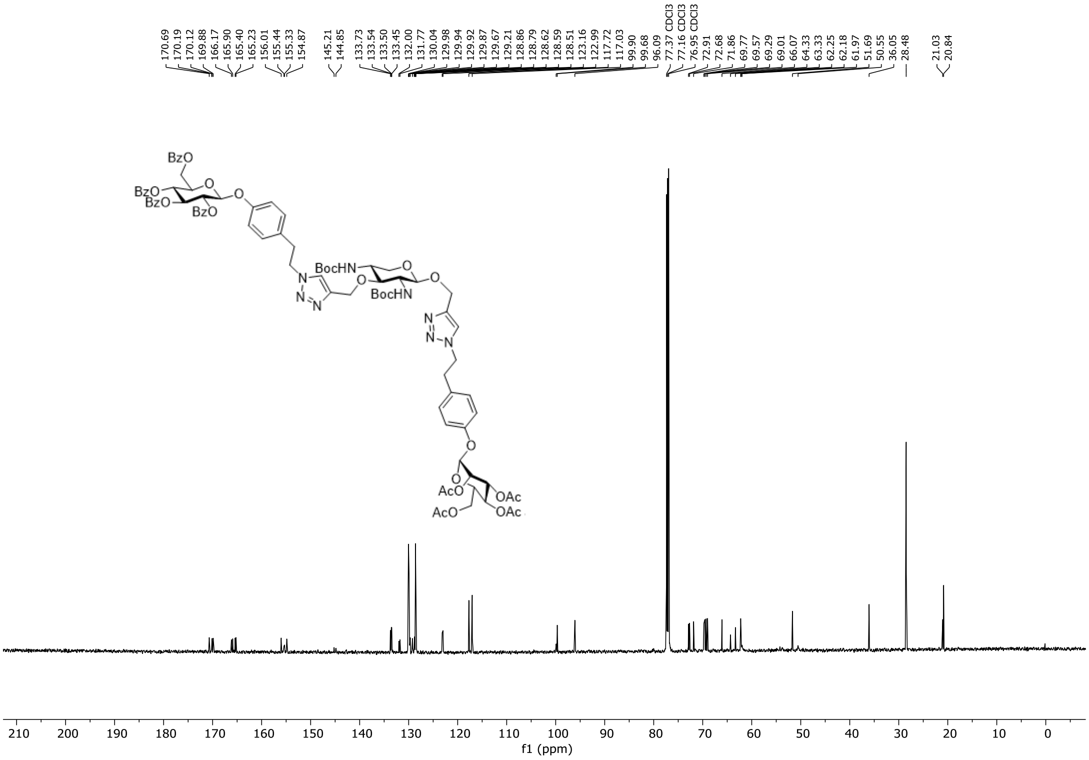

Figure S49 ^{13}C NMR spectrum of compound **23** (151 MHz, $CDCl_3$, 298 K).

4.24 Glycoconjugate 24

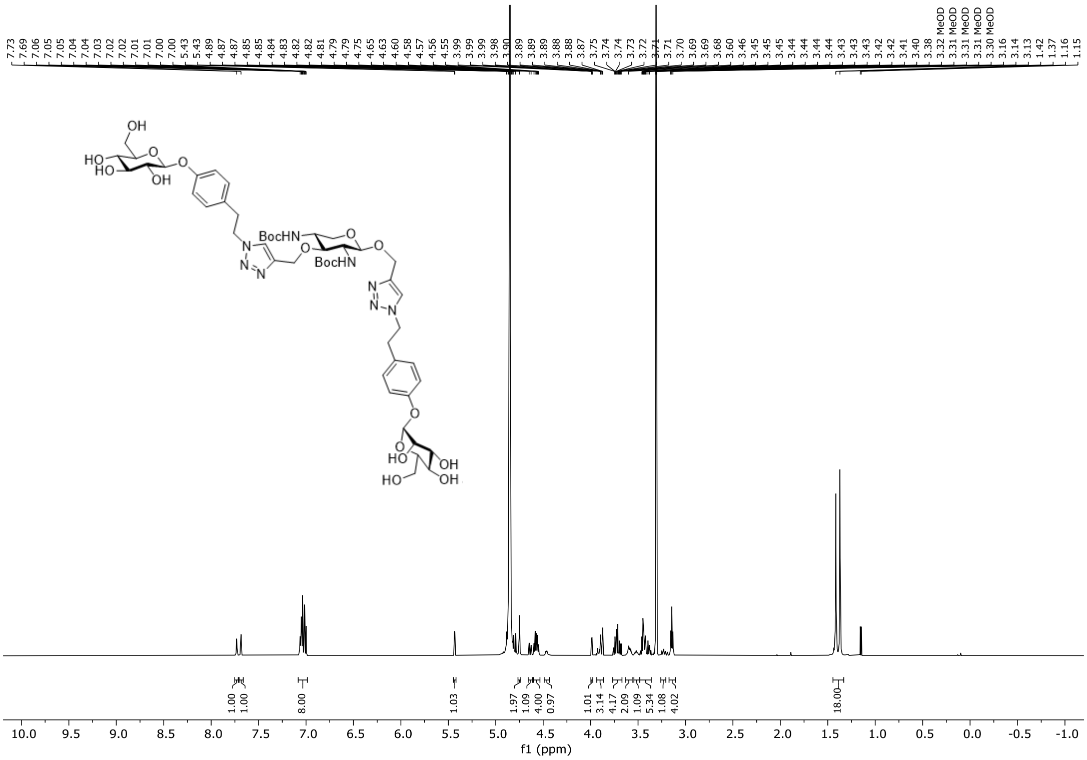

Figure S50 ^{1}H NMR spectrum of compound **24** (600 MHz, CD_3OD, 298 K).

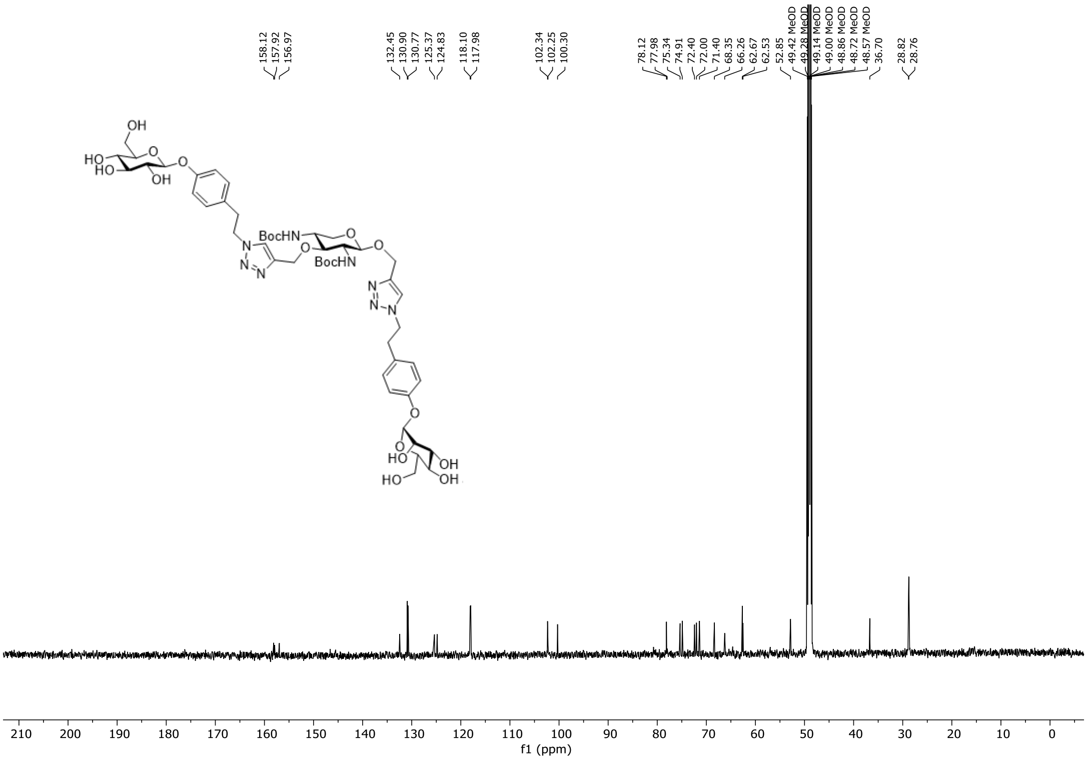

Figure S51 ^{13}C NMR spectrum of compound **24** (151 MHz, CD_3OD, 298 K).

4.25 Glycoconjugate 25

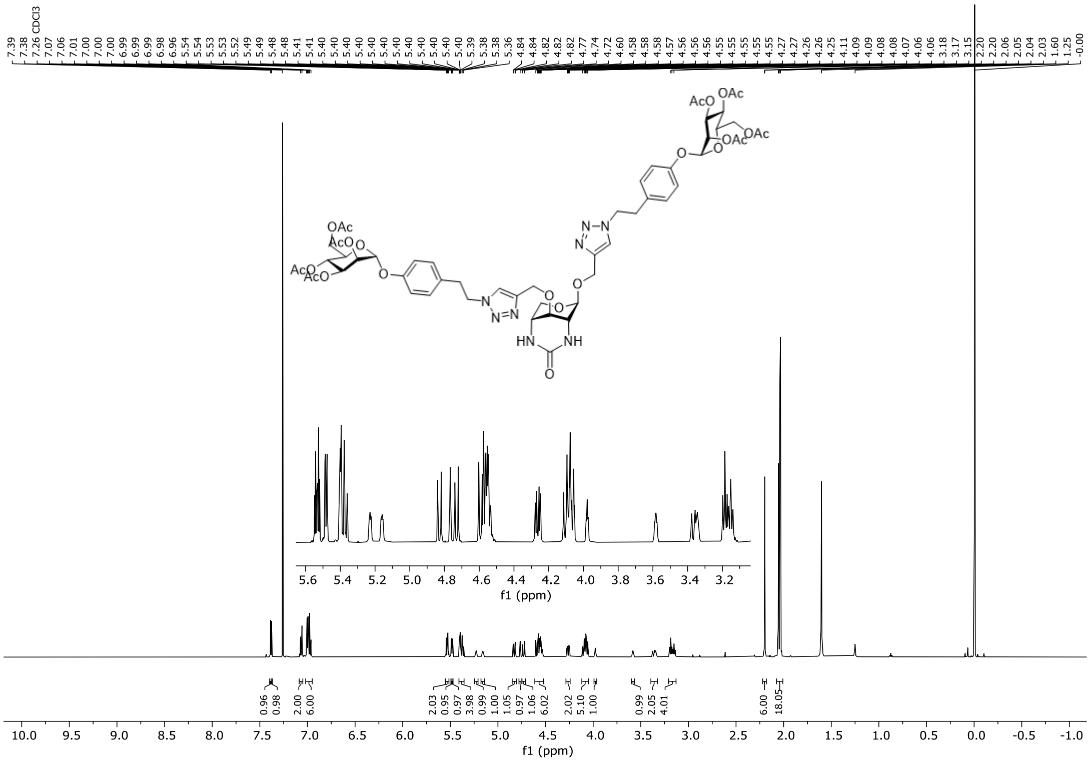

Figure S52 ^{1}H NMR spectrum of compound **25** (600 MHz, $CDCl_3$, 298 K).

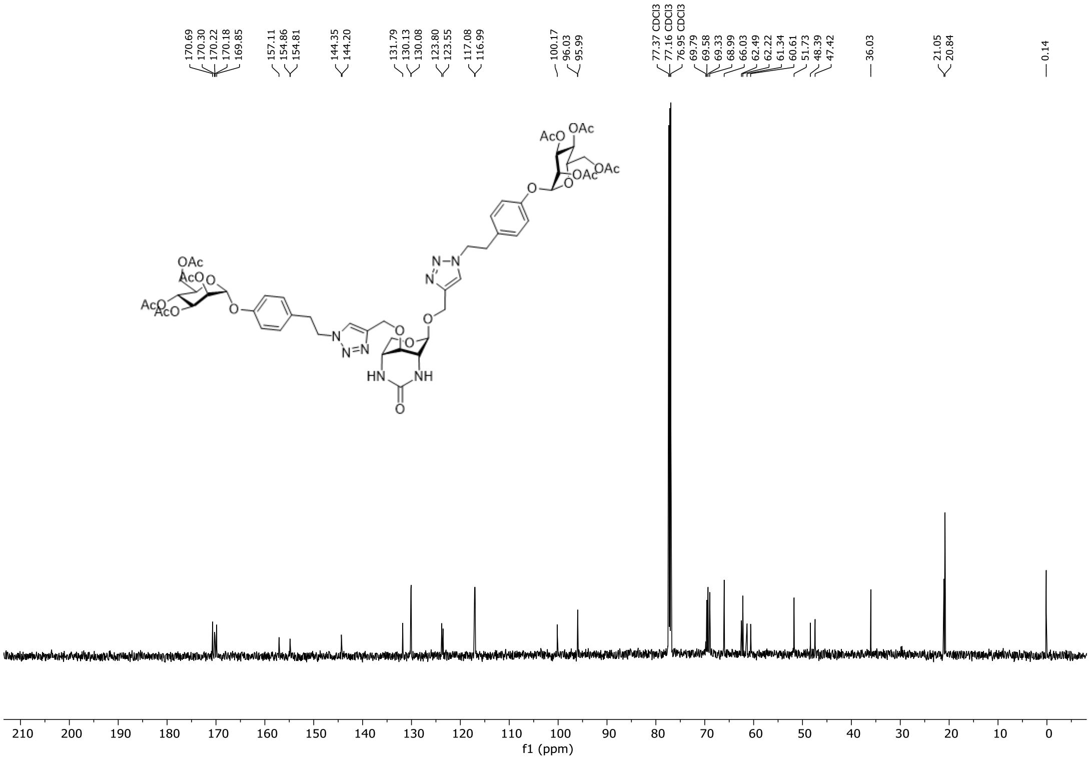

Figure S53 ^{13}C NMR spectrum of compound **25** (151 MHz, $CDCl_3$, 298 K).

4.26 Glycoconjugate 26

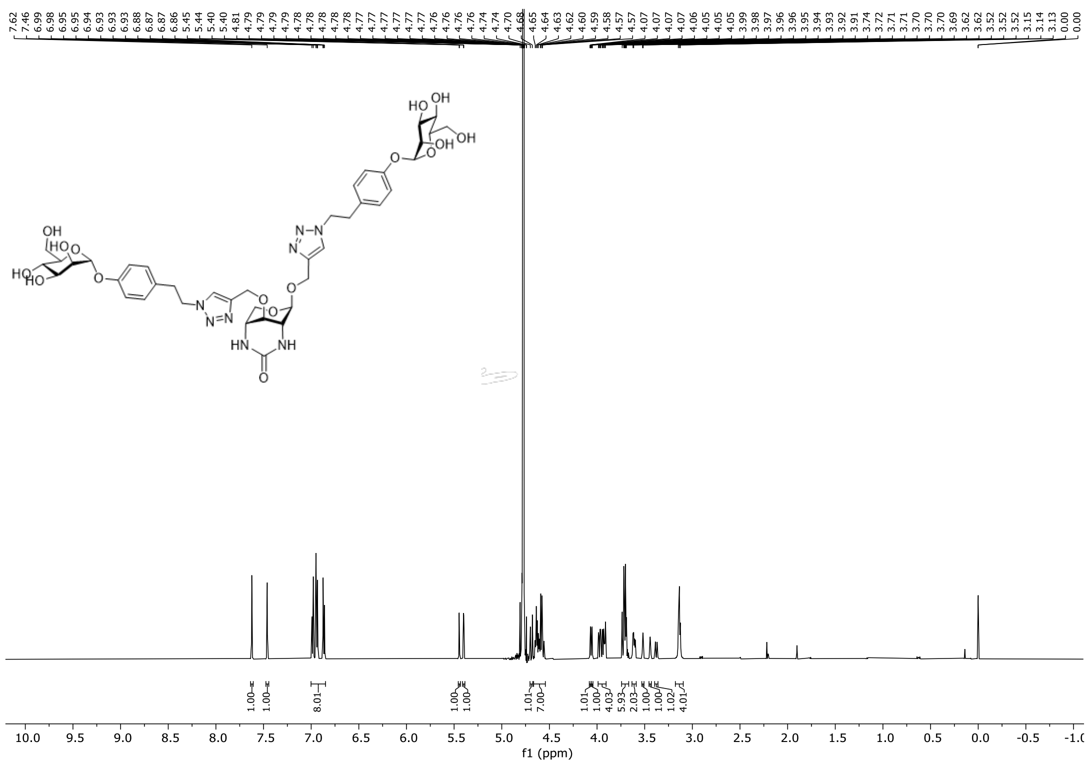

Figure S54 ^{1}H NMR spectrum of compound **26** (600 MHz, D_2O, 298 K).

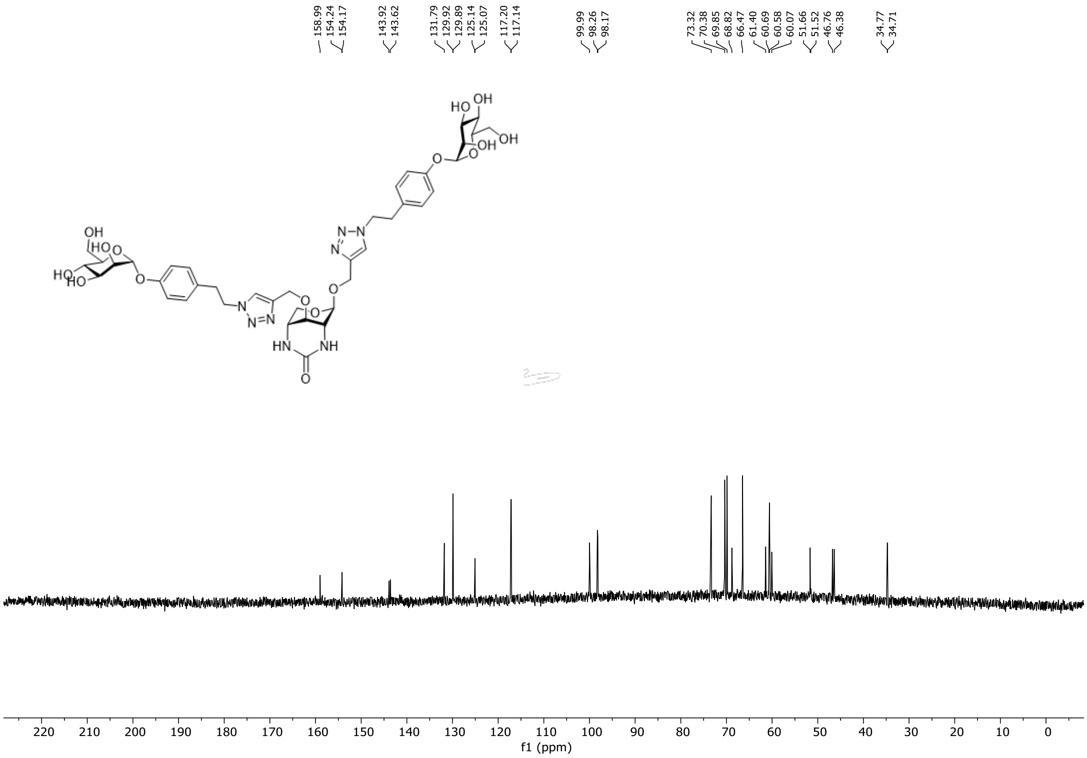

Figure S55 ^{13}C NMR spectrum of compound **26** (151 MHz, D_2O, 298 K).

5 References

1. Yuasa, H.; Hashimoto, H. Bending Trisaccharides by a Chelation-Induced Ring Flip of a Hinge-Like Monosaccharide Unit. *J. Am. Chem. Soc.* **1999**, *121*, 5089–5090, doi:10.1021/ja984062p.
2. Helm, R.F.; Ralph, J.; Anderson, L. Regioselective Protection Strategies for d-Xylopyranosides. *J. Org. Chem.* **1991**, *56*, 7015–7021, doi:10.1021/jo00025a013.
3. Underlin, E.N.; Böhm, M.; Madsen, R. Synthesis of Arabinoxylan Oligosaccharides by Preactivation-Based Iterative Glycosylations. *J. Org. Chem.* **2019**, *84*, 16036–16054, doi:10.1021/acs.joc.9b02529.
4. Vaz, B.; Otero, L.; Álvarez, R.; de Lera, Á.R. Total Synthesis of Enantiopure Pyrrhoxanthin: Alternative Methods for the Stereoselective Preparation of 4-Alkylidenebutenolides. *Chem. Eur. J.* **2013**, *19*, 13065–13074, doi:10.1002/chem.201301873.
5. Makarem, A.; Klika, K.D.; Litau, G.; Remde, Y.; Kopka, K. HBED-NN: A Bifunctional Chelator for Constructing Radiopharmaceuticals. *J. Org. Chem.* **2019**, *84*, 7501–7508, doi:10.1021/acs.joc.9b00832.
6. Mondal, M.; Unver, M.Y.; Pal, A.; Bakker, M.; Berrier, S.P.; Hirsch, A.K.H. Fragment-Based Drug Design Facilitated by Protein-Templated Click Chemistry: Fragment Linking and Optimization of Inhibitors of the Aspartic Protease Endothiapepsin. *Chem. Eur. J.* **2016**, *22*, 14826–14830, doi:10.1002/chem.201603001.
7. Igde, S.; Röblitz, S.; Müller, A.; Kolbe, K.; Boden, S.; Fessele, C.; Lindhorst, T.K.; Weber, M.; Hartmann, L. Linear Precision Glycomacromolecules with Varying Interligand Spacing and Linker Functionalities Binding to Concanavalin A and the Bacterial Lectin FimH. *Macromol. Biosci.* **2017**, *17,* 1700198, doi:10.1002/mabi.201700198.
8. Vasquez, O.; Alibrandi, A.; Bennett, C.S. De Novo Synthetic Approach to 2,4-Diamino-2,4,6-trideoxyhexoses (DATDH): Bacterial and Rare Deoxy-Amino Sugars. *Org. Lett.* **2023**, *25*, 7873–7877, doi:10.1021/acs.orglett.3c03106.
9. Huggins, M.T.; Kesharwani, T.; Buttrick, J.; Nicholson, C. Variable Temperature NMR Experiment Studying Restricted Bond Rotation. *J. Chem. Educ.* **2020**, *97*, 1425–1429, doi:10.1021/acs.jchemed.0c00057.
10. Bragg, R.A.; Clayden, J.; Morris, G.A.; Pink, J.H. Stereodynamics of Bond Rotation in Tertiary Aromatic Amides. *Chem. Eur. J.* **2002**, *8*, 1279–1289, doi:10.1002/1521-3765(20020315)8:6<1279:AID-CHEM1279>3.0.CO;2-7.
11. Owens, N.W.; Braun, C.; Schweizer, F. Tuning of the Prolyl trans/cis-Amide Rotamer Population by Use of C-Glucosylproline Hybrids. *J. Org. Chem.* **2007**, *72*, 4635–4643, doi:10.1021/jo0700833.
12. Hartmann, M.; Horst, A.K.; Klemm, P.; Lindhorst, T.K. A kit for the investigation of live Escherichia coli cell adhesion to glycosylated surfaces. *Chem. Commun.* **2010**, *46*, 330–332, doi:10.1039/b922525k.
13. Reisner, A.; Haagensen, J.A.J.; Schembri, M.A.; Zechner, E.L.; Molin, S. Development and maturation of Escherichia coli K-12 biofilms. *Mol. Microbiol.* **2003**, *48*, 933–946, doi:10.1046/j.1365-2958.2003.03490.x.
